# Supplementary material for: PAPPI: Personalized analysis of plantar pressure images using statistical modelling and parametric mapping
Source: PLoS One. 2020 Feb 27;15(2):e0229685. doi: 10.1371/journal.pone.0229685 (PMC7046232; doi:10.1371/journal.pone.0229685)
Supplement: S1 File — Plantar pressure predictions, image registration results, and statistical parametric maps (thresholded and non-thresholded) for each of the 69 hallux valgus cases in this study. (PDF) [file pone.0229685.s001.pdf]

Registration Results, Patient 1 (left foot)

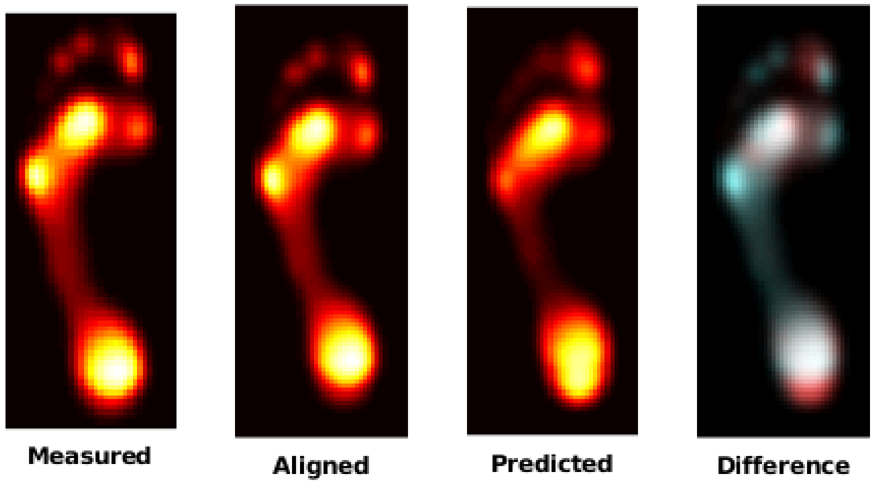

Statistical Results, Patient 1 (left foot)

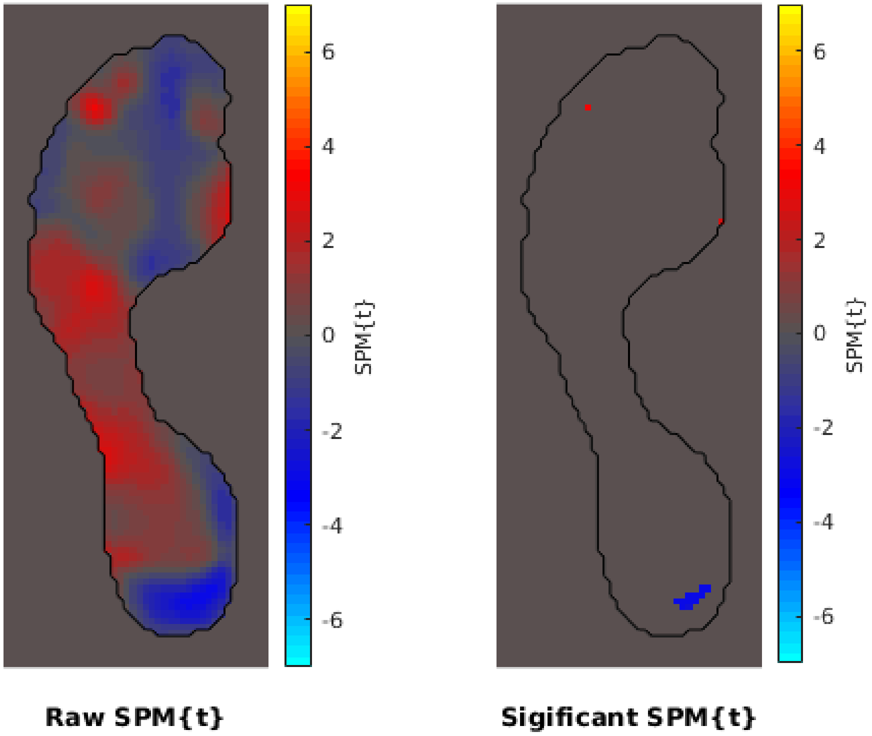

Registration Results, Patient 1 (right foot)

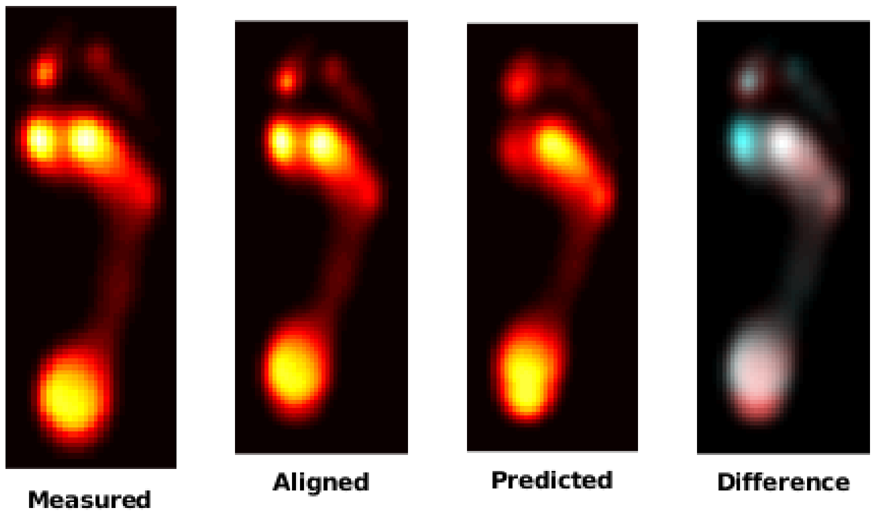

Statistical Results, Patient 1 (right foot)

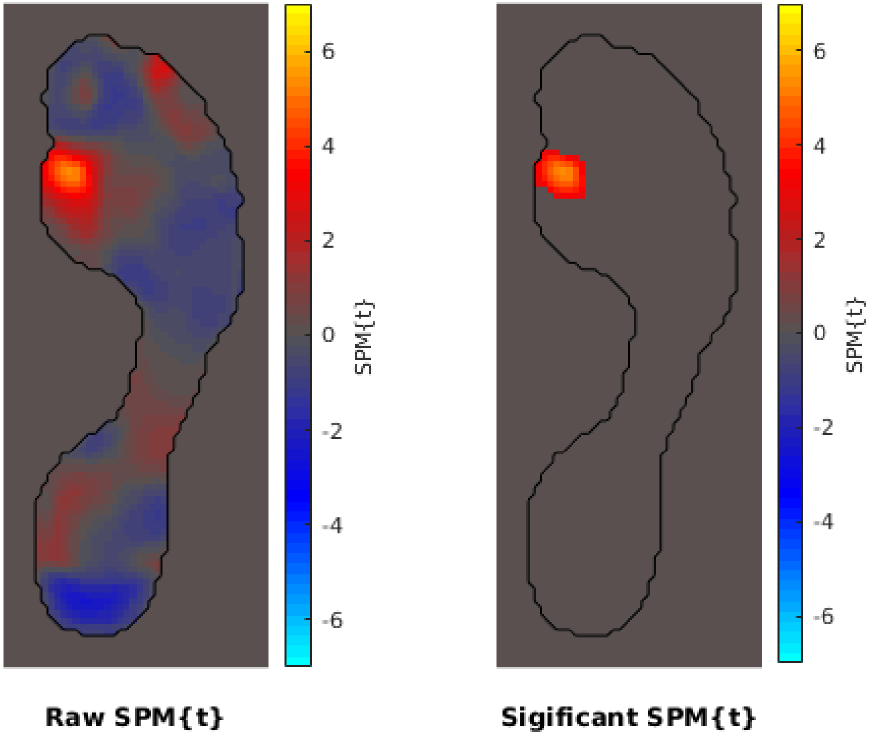

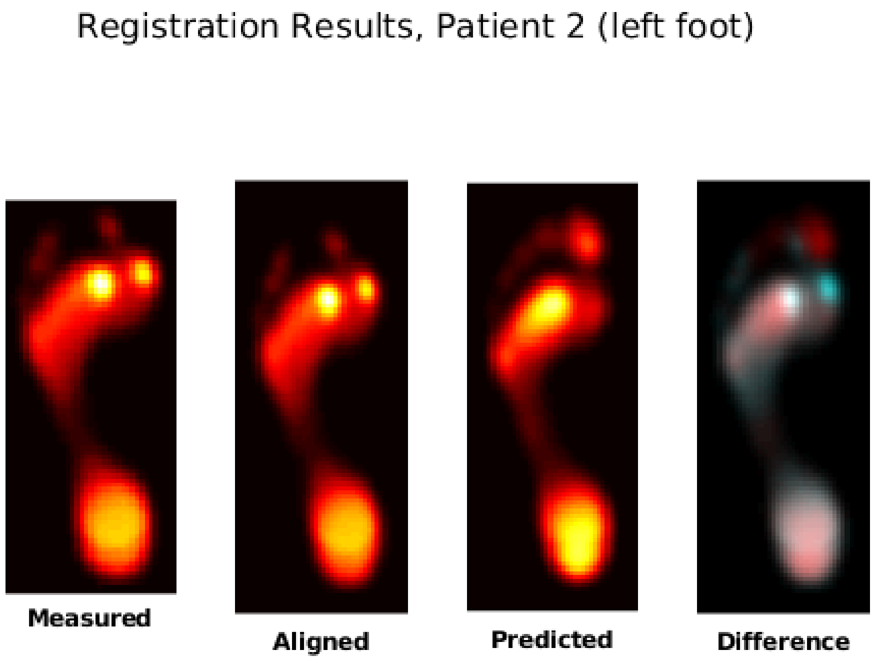
Registration Results, Patient 2 (left foot)
Measured
Aligned
Predicted
Difference

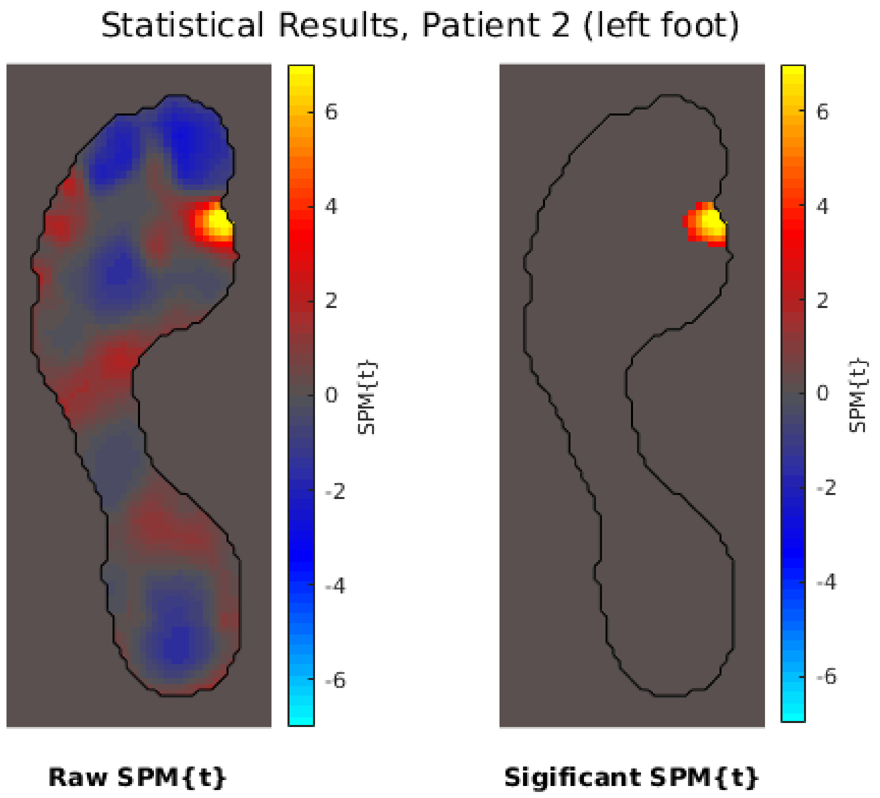
Statistical Results, Patient 2 (left foot)
6
4
2
0
-2
-4
-6
SPM{t}
Raw SPM{t}
Sigificant SPM{t}

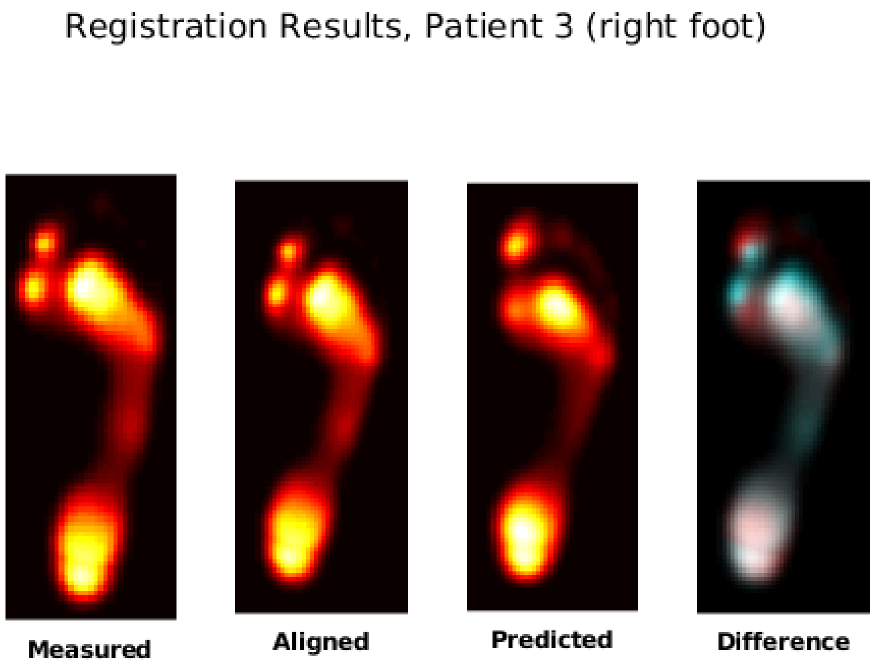
Registration Results, Patient 3 (right foot)
Measured
Aligned
Predicted
Difference

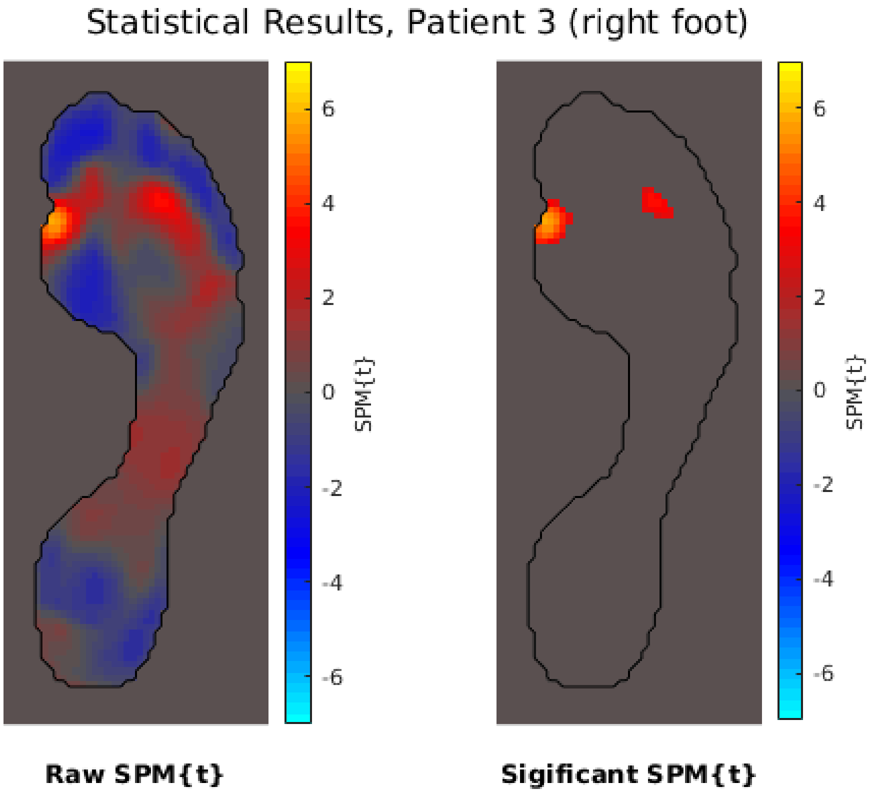
Statistical Results, Patient 3 (right foot)
6
4
2
0
-2
-4
-6
SPM{t}
Raw SPM{t}
Sigificant SPM{t}

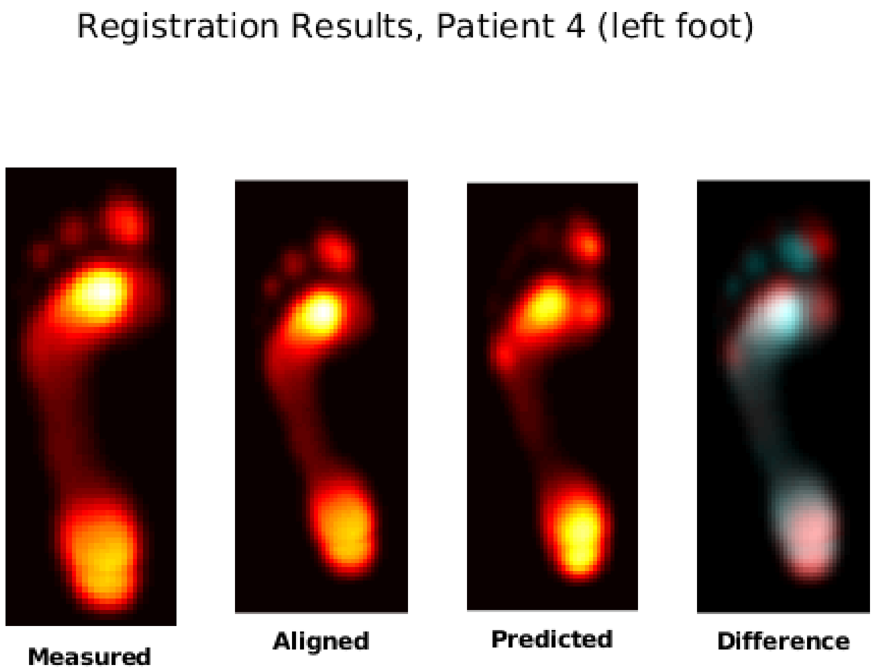
Registration Results, Patient 4 (left foot)
Measured
Aligned
Predicted
Difference

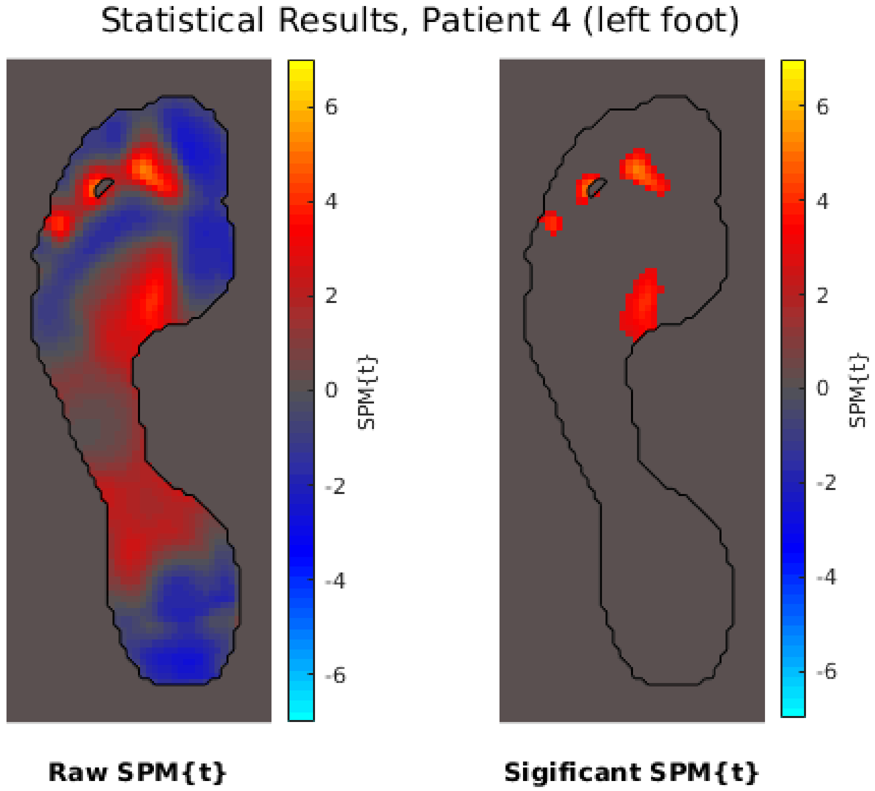
Statistical Results, Patient 4 (left foot)
6
4
2
0
-2
-4
-6
SPM{t}
Raw SPM{t}
Sigificant SPM{t}

Registration Results, Patient 4 (right foot)

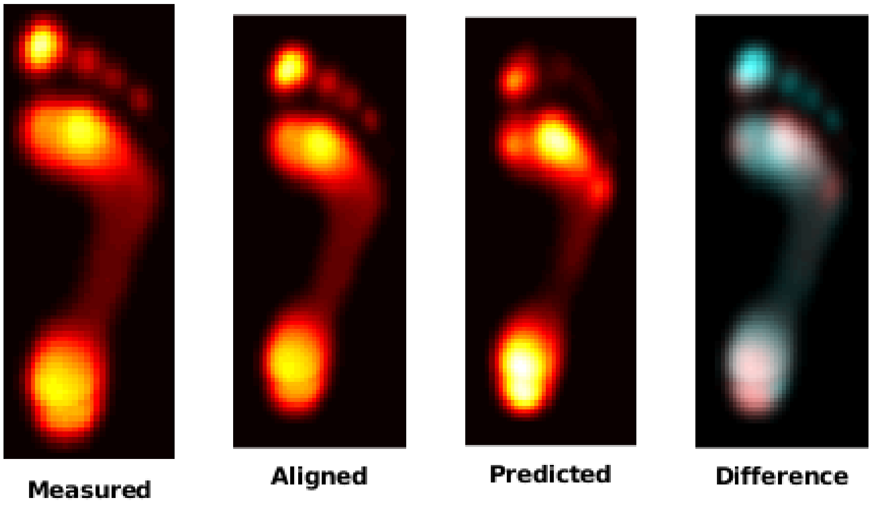

Statistical Results, Patient 4 (right foot)

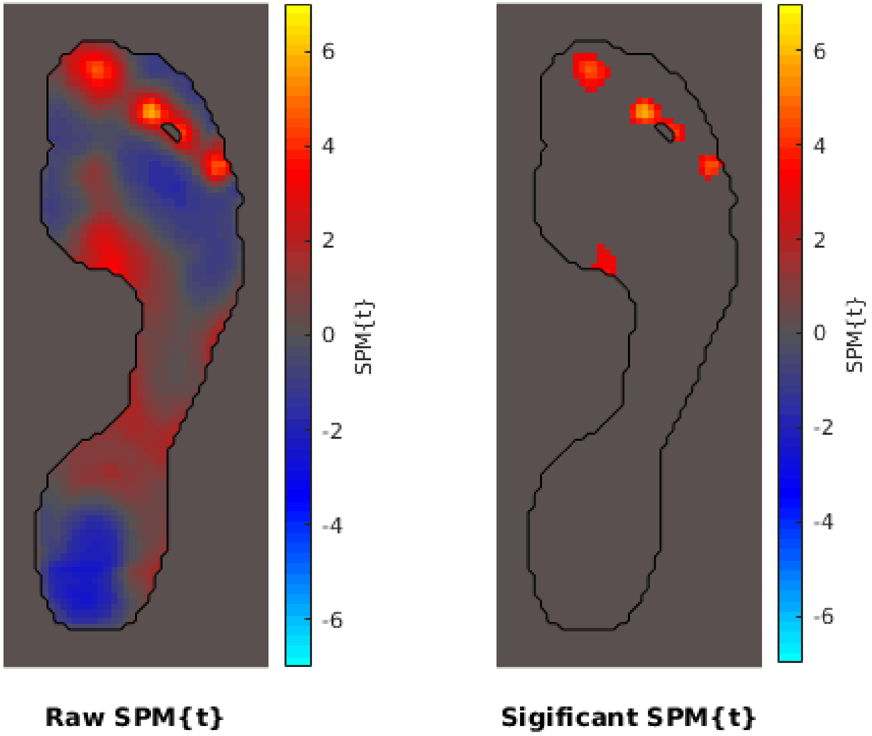

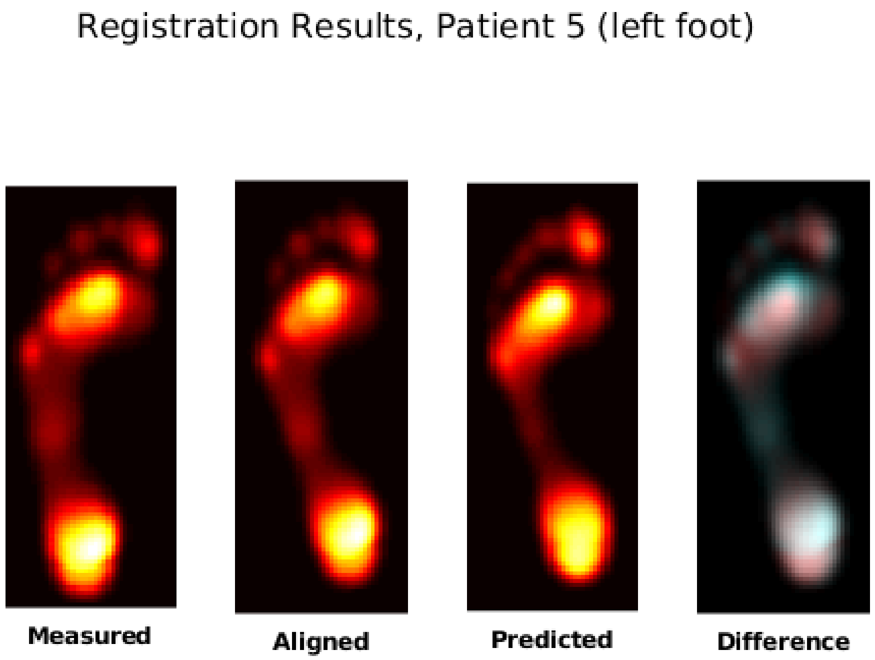
Registration Results, Patient 5 (left foot)
Measured
Aligned
Predicted
Difference

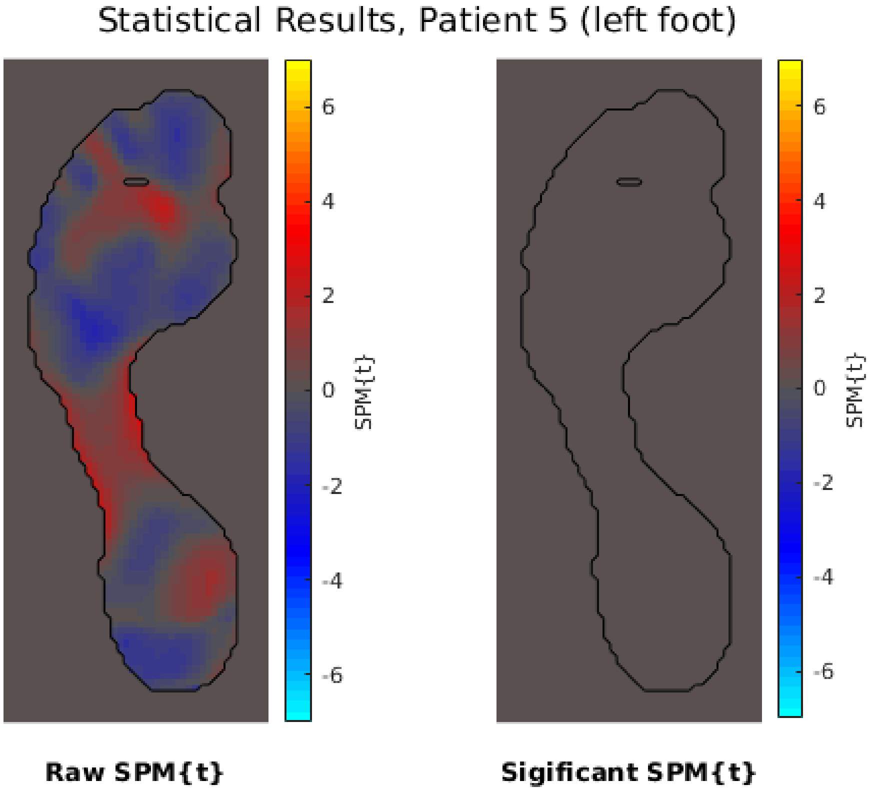
Statistical Results, Patient 5 (left foot)
6
4
2
0
-2
-4
-6
SPM{t}
Raw SPM{t}
Sigificant SPM{t}

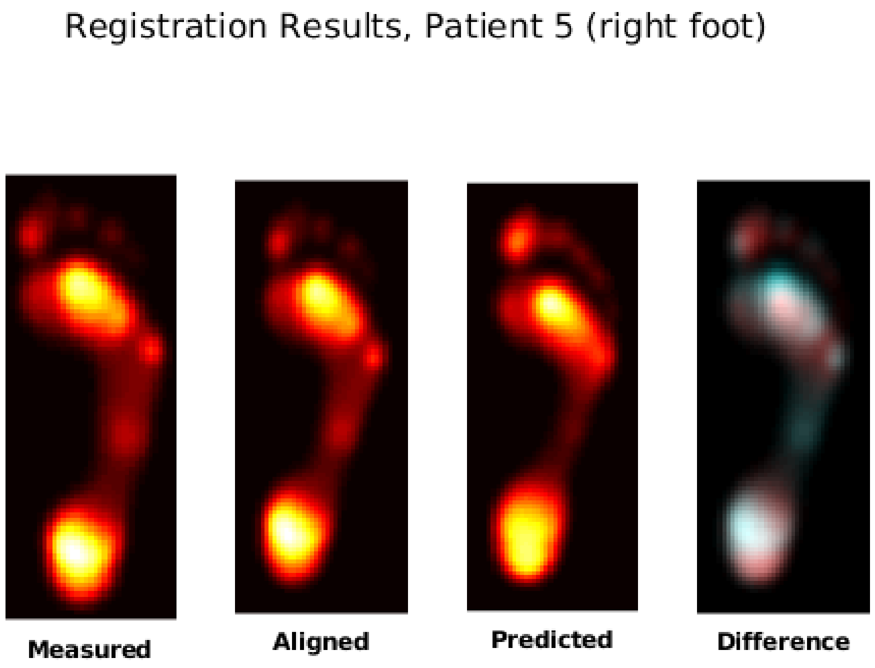
Registration Results, Patient 5 (right foot)
Measured
Aligned
Predicted
Difference

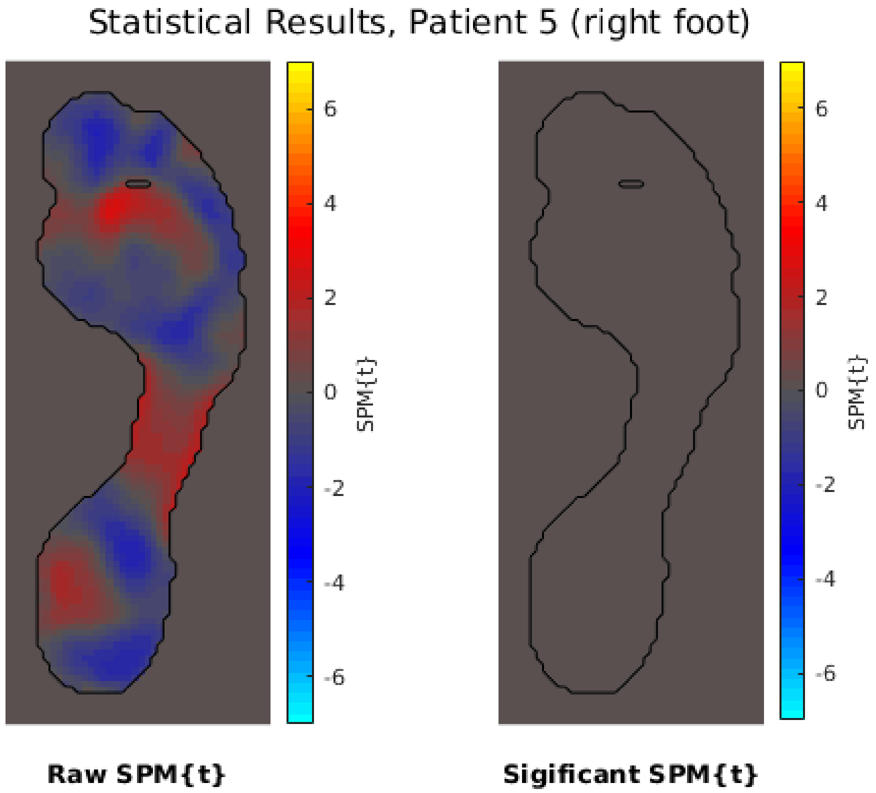
Statistical Results, Patient 5 (right foot)
6
4
2
0
-2
-4
-6
SPM{t}
Raw SPM{t}
Sigificant SPM{t}

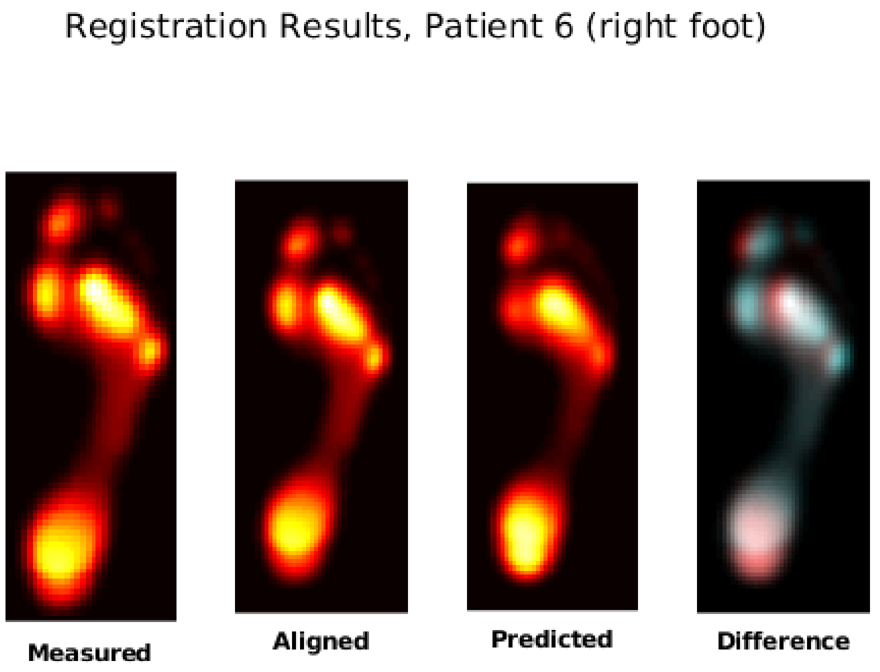
Registration Results, Patient 6 (right foot)
Measured
Aligned
Predicted
Difference

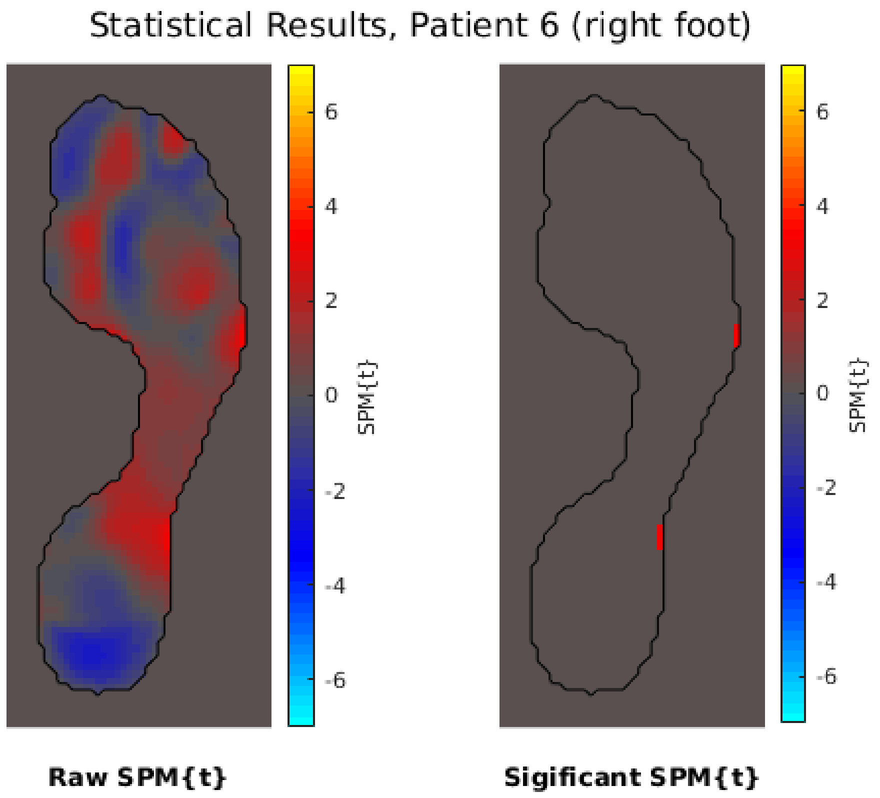
Statistical Results, Patient 6 (right foot)
6
4
2
0
-2
-4
-6
SPM{t}
Raw SPM{t}
Sigificant SPM{t}

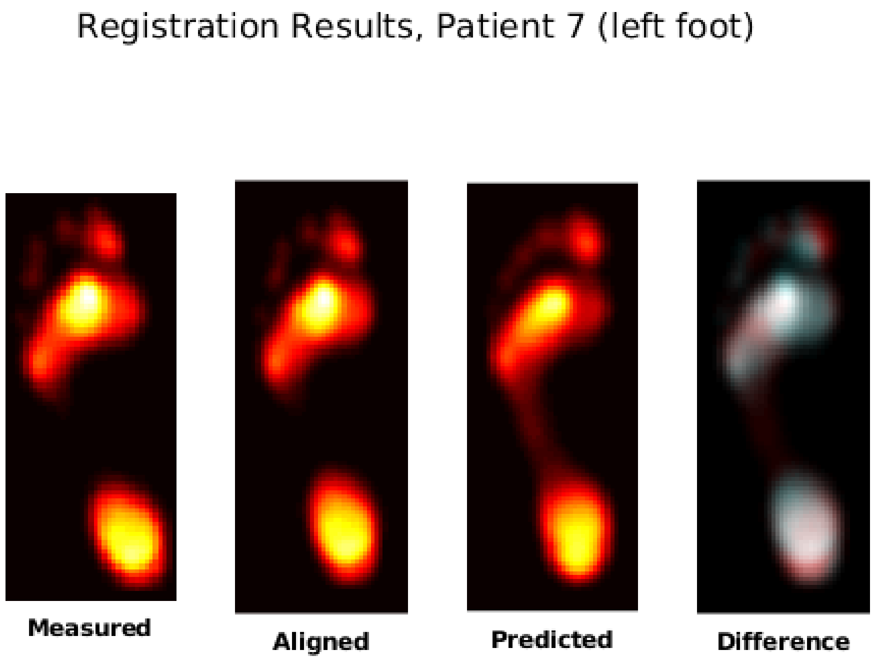
Registration Results, Patient 7 (left foot)
Measured
Aligned
Predicted
Difference

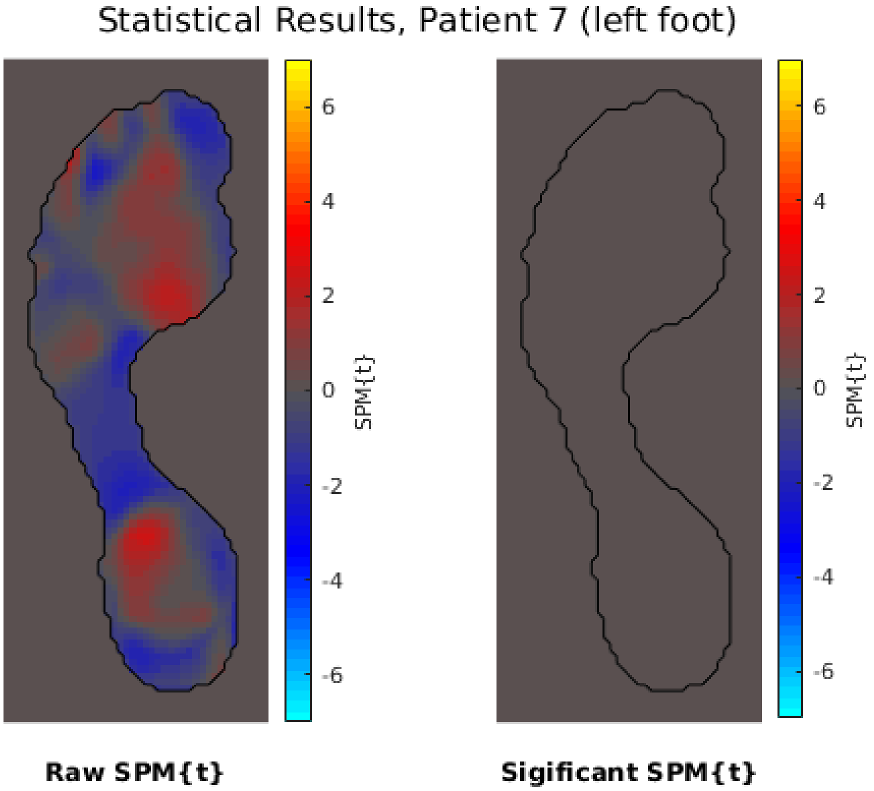
Statistical Results, Patient 7 (left foot)
6
4
2
0
-2
-4
-6
SPM{t}
Raw SPM{t}
Sigificant SPM{t}

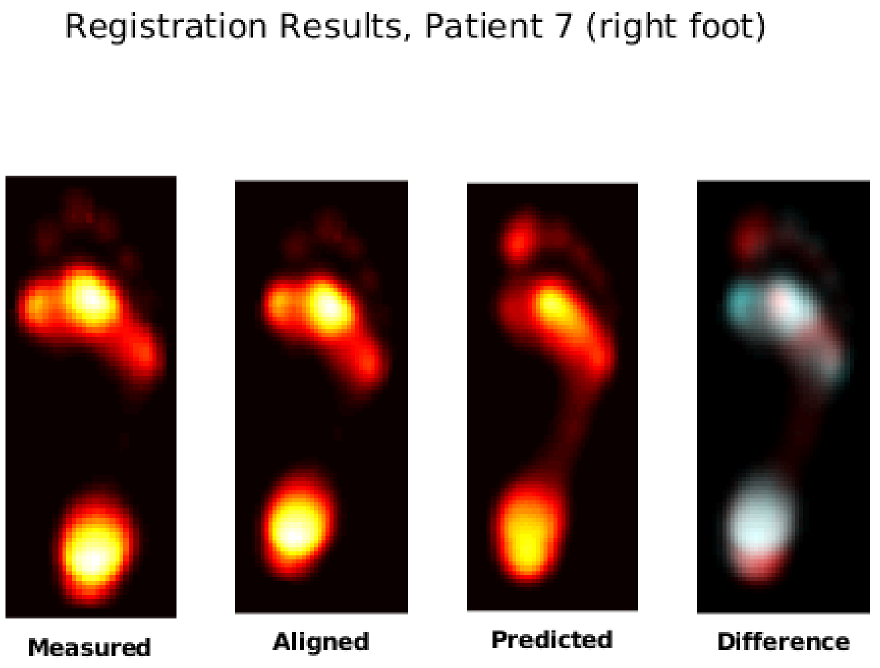
Registration Results, Patient 7 (right foot)
Measured
Aligned
Predicted
Difference

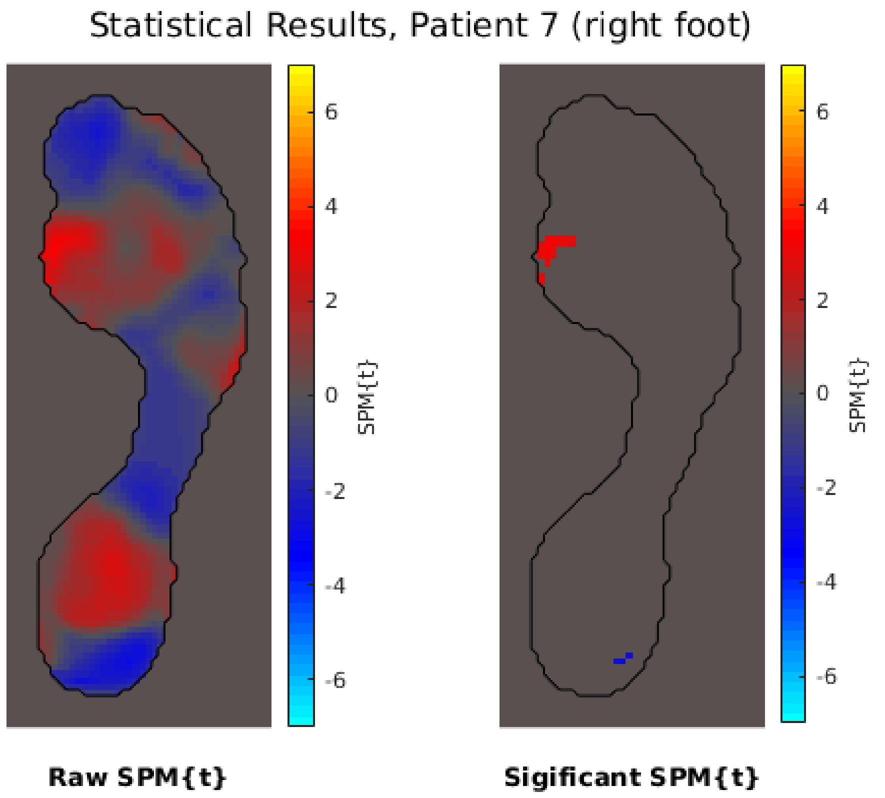
Statistical Results, Patient 7 (right foot)
6
4
2
0
-2
-4
-6
SPM{t}
Raw SPM{t}
Sigificant SPM{t}

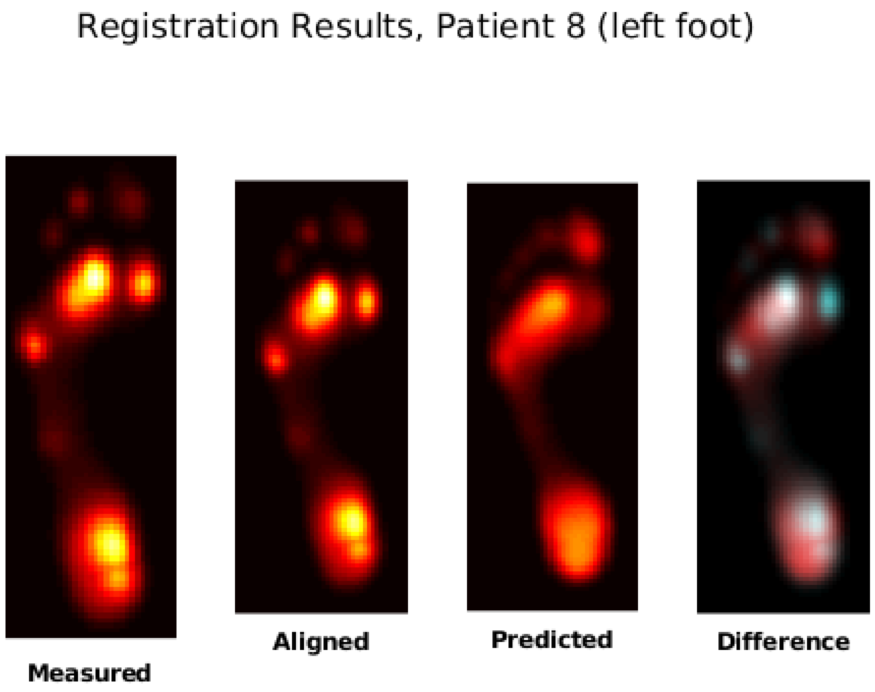
Registration Results, Patient 8 (left foot)
Measured
Aligned
Predicted
Difference

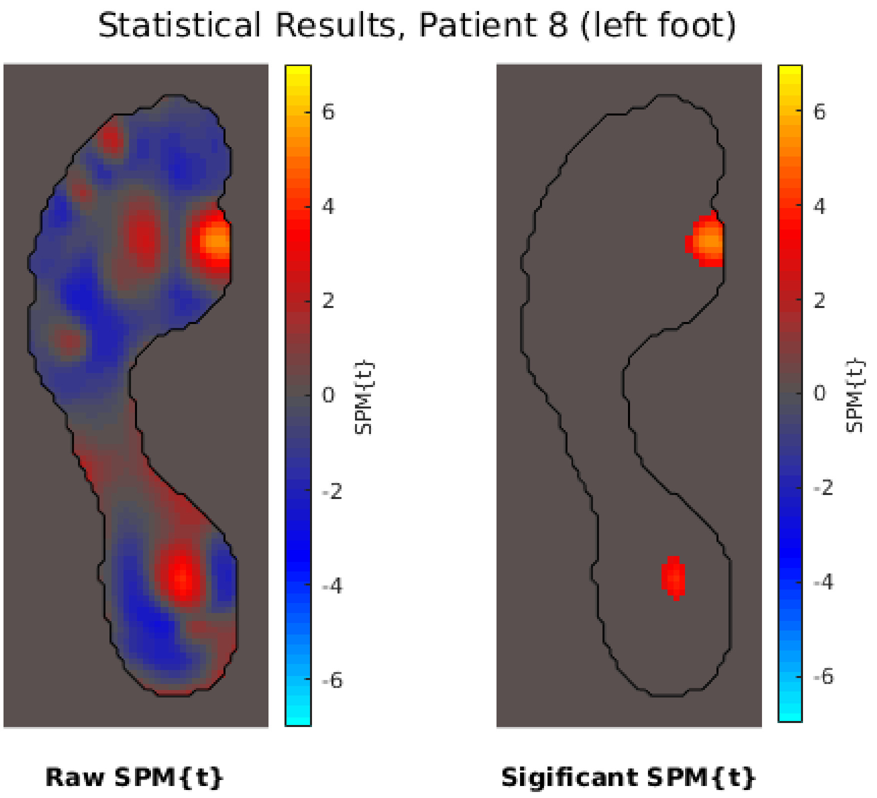
Statistical Results, Patient 8 (left foot)
6
4
2
0
-2
-4
-6
SPM{t}
Raw SPM{t}
Sigificant SPM{t}

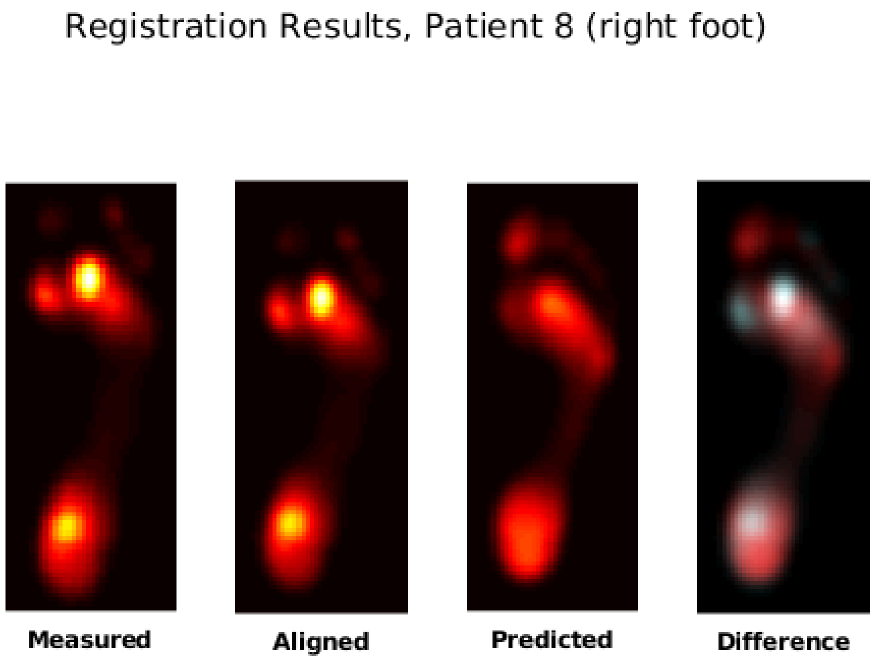
Registration Results, Patient 8 (right foot)
Measured
Aligned
Predicted
Difference

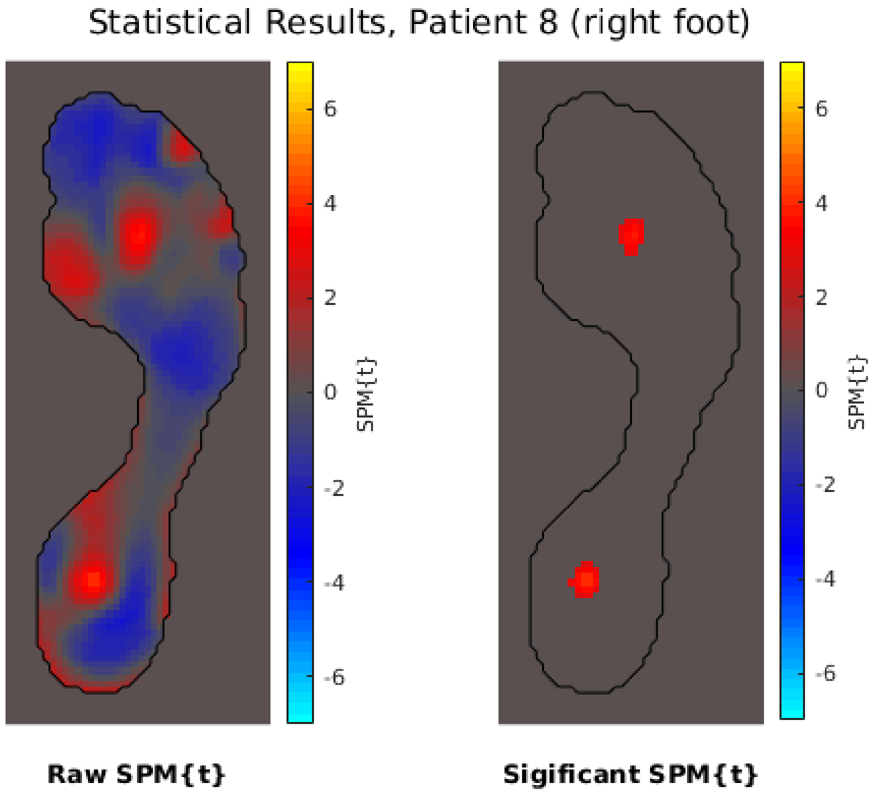
Statistical Results, Patient 8 (right foot)
6
4
2
0
-2
-4
-6
SPM{t}
Raw SPM{t}
Sigificant SPM{t}

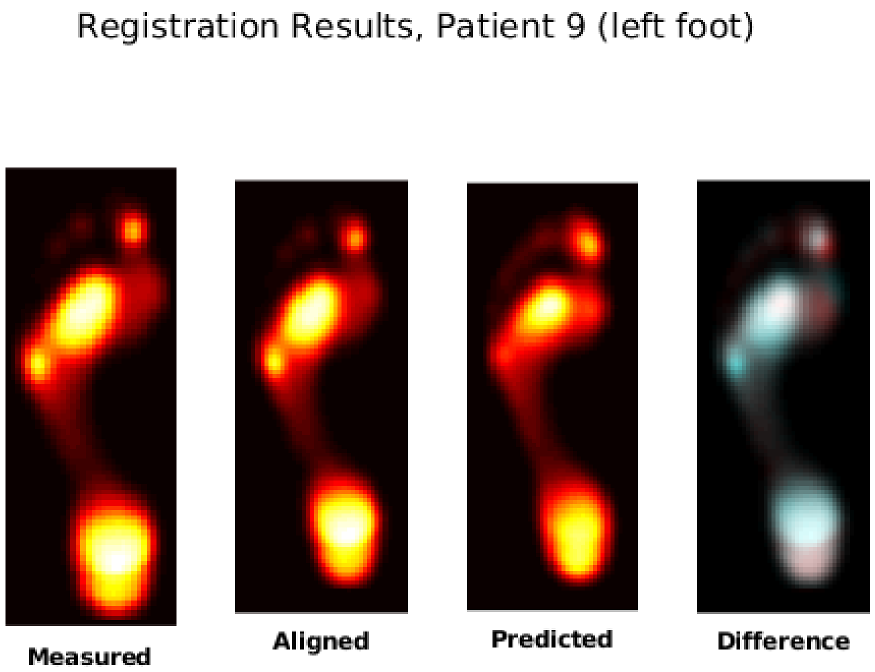
Registration Results, Patient 9 (left foot)
Measured
Aligned
Predicted
Difference

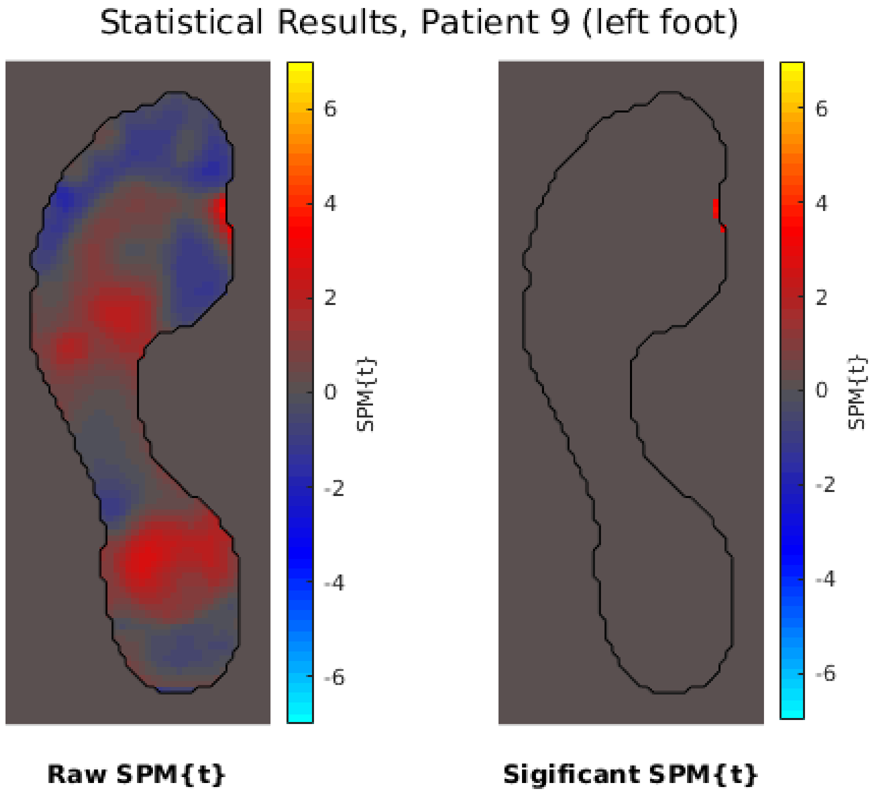
Statistical Results, Patient 9 (left foot)
6
4
2
0
-2
-4
-6
SPM{t}
Raw SPM{t}
Sigificant SPM{t}

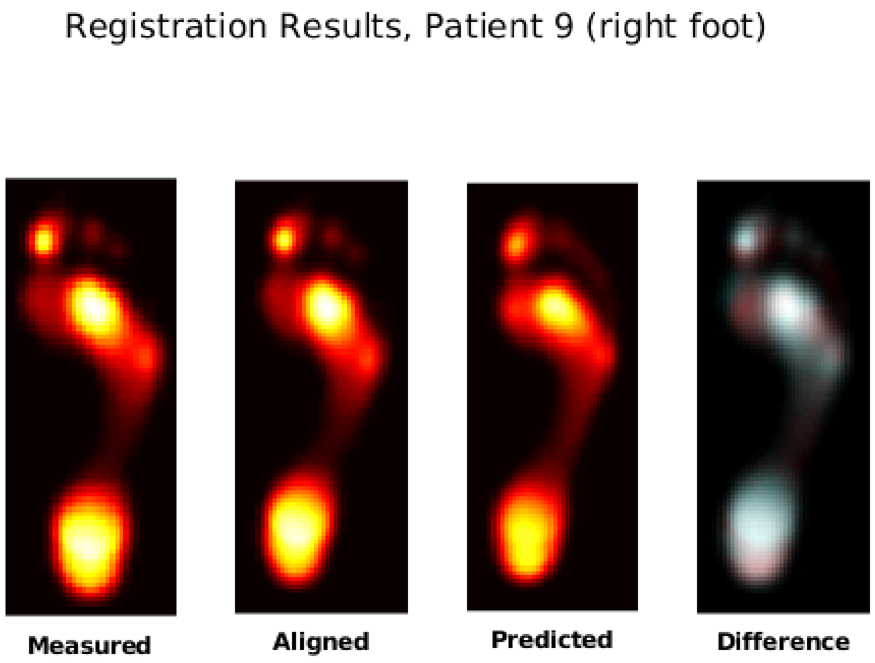
Registration Results, Patient 9 (right foot)
Measured
Aligned
Predicted
Difference

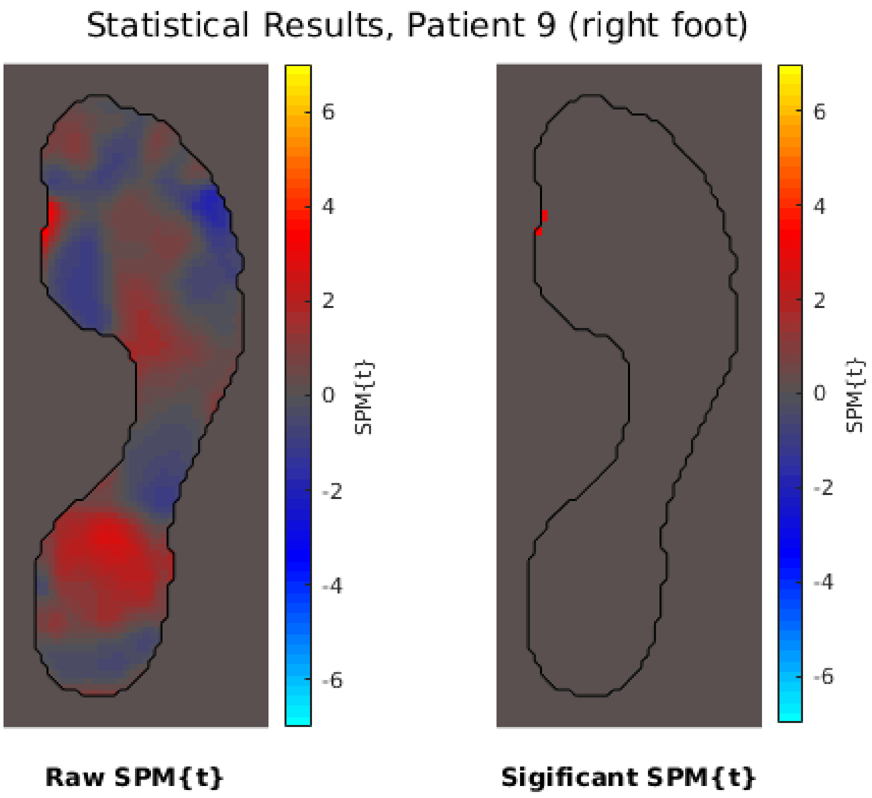
Statistical Results, Patient 9 (right foot)
6
4
2
0
-2
-4
-6
SPM{t}
Raw SPM{t}
Sigificant SPM{t}

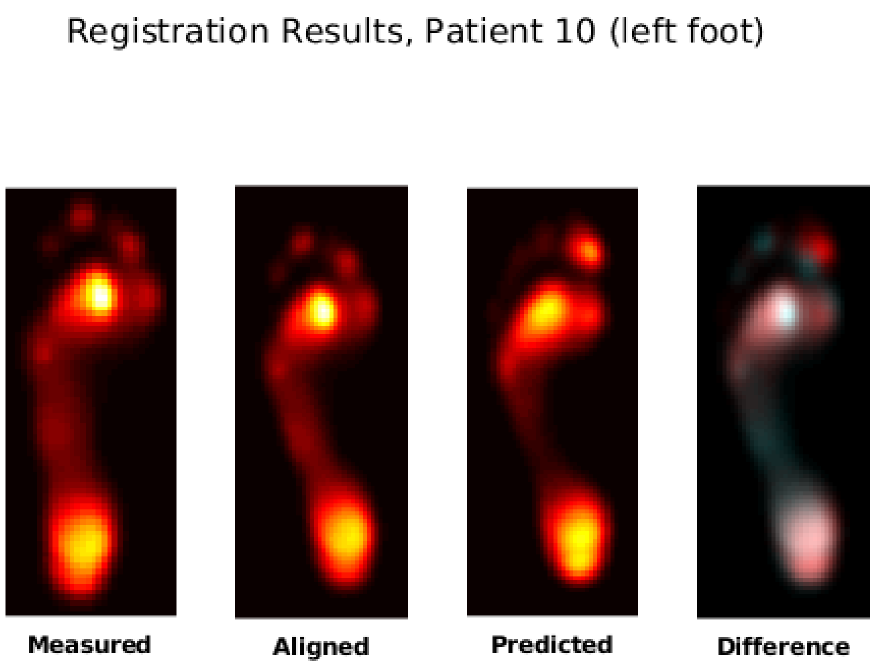
Registration Results, Patient 10 (left foot)
Measured
Aligned
Predicted
Difference

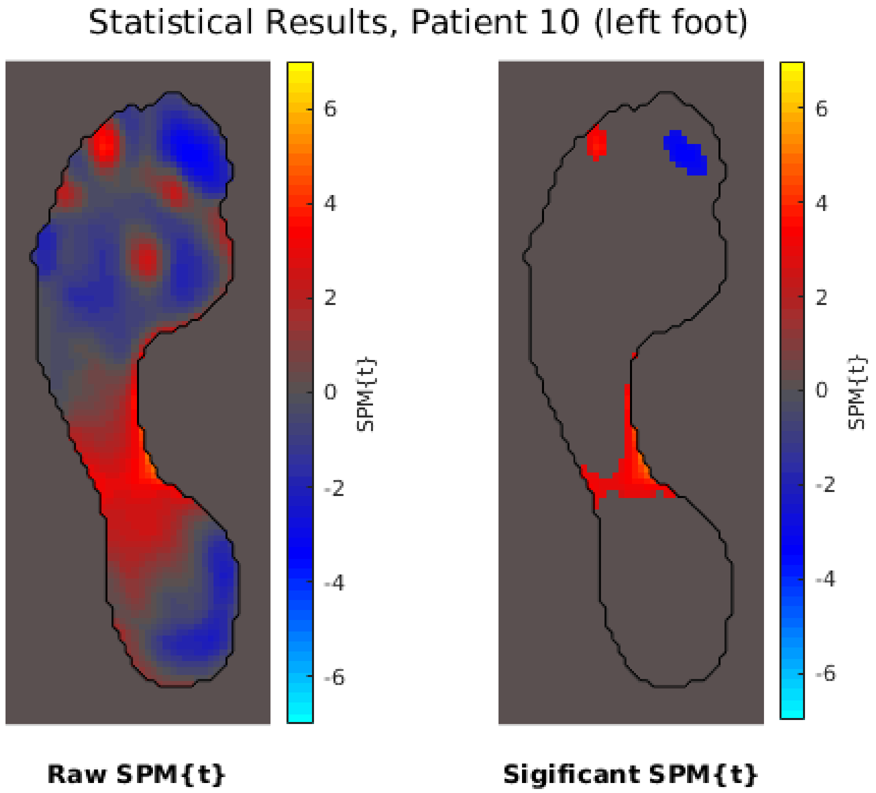
Statistical Results, Patient 10 (left foot)
6
4
2
0
-2
-4
-6
SPM{t}
Raw SPM{t}
Sigificant SPM{t}

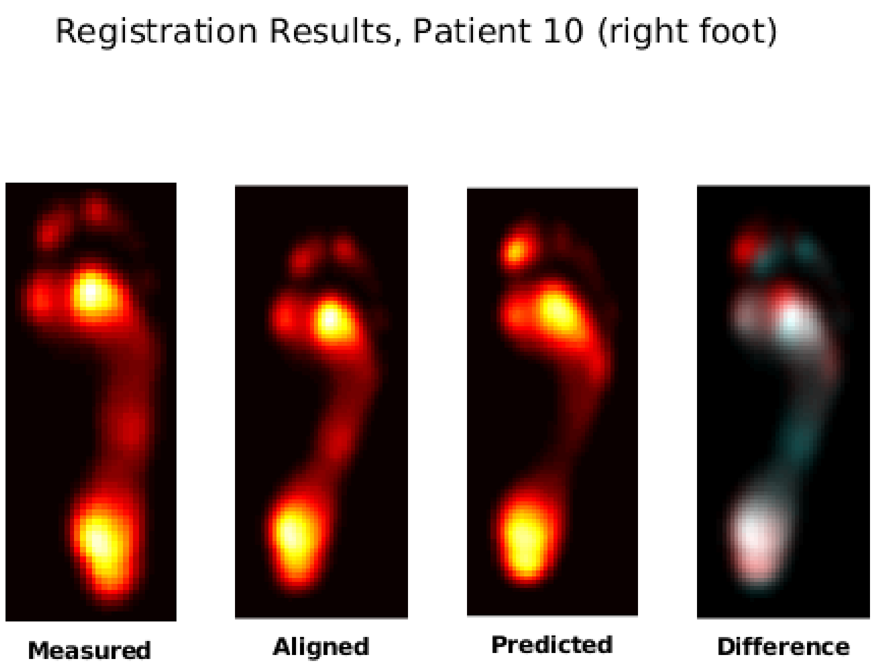
Registration Results, Patient 10 (right foot)
Measured
Aligned
Predicted
Difference

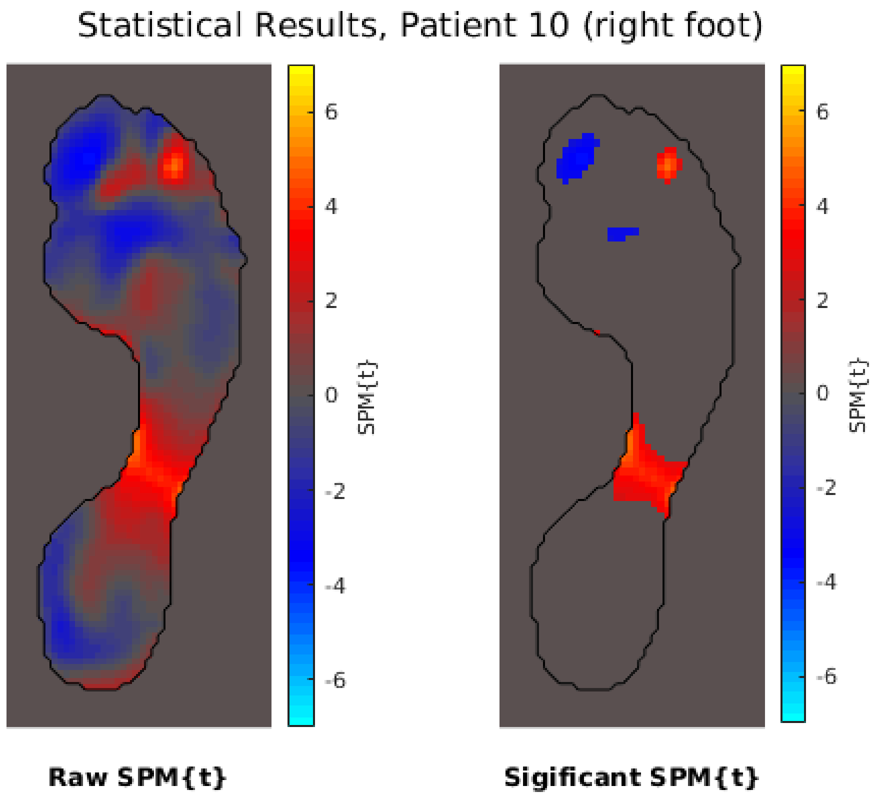
Statistical Results, Patient 10 (right foot)
6
4
2
0
-2
-4
-6
SPM{t}
Raw SPM{t}
Sigificant SPM{t}

Registration Results, Patient 11 (left foot)

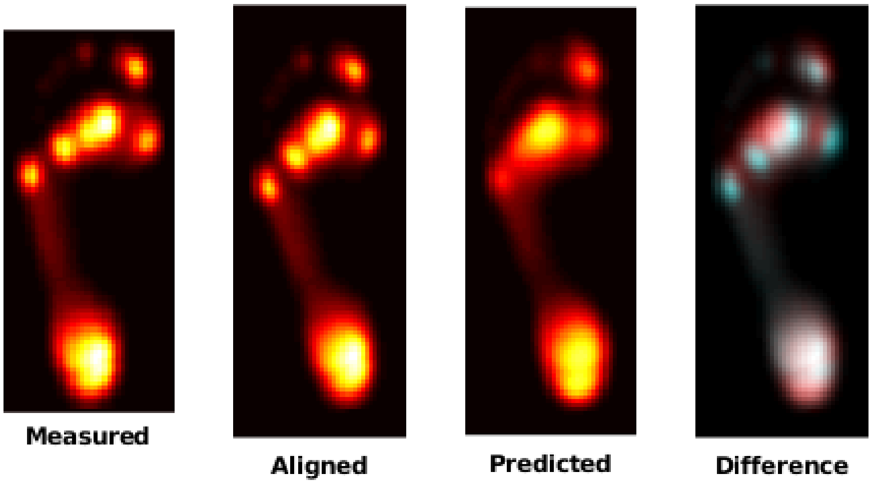

Statistical Results, Patient 11 (left foot)

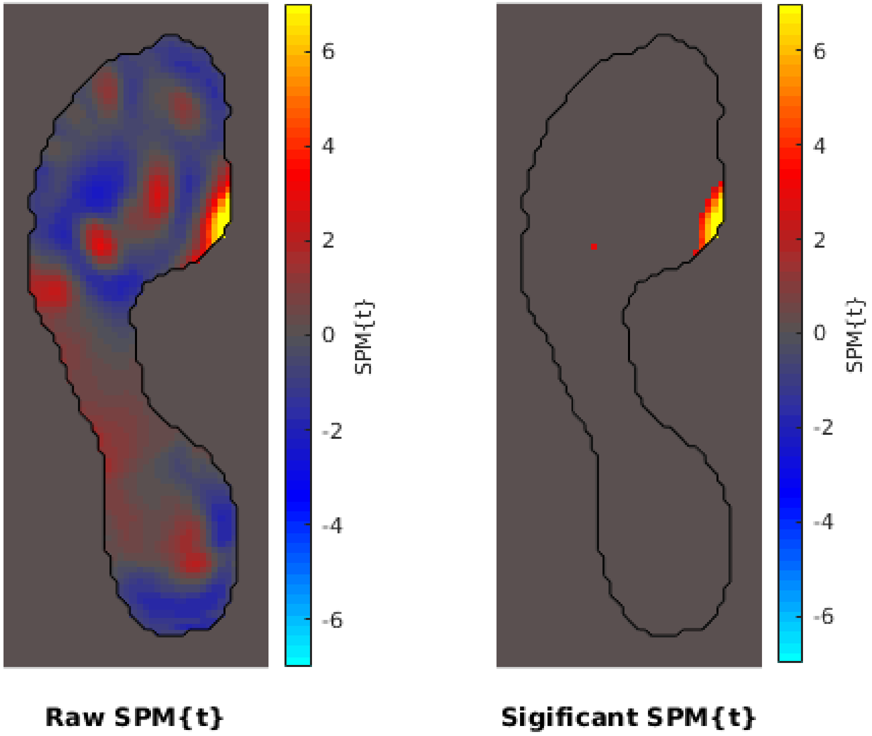

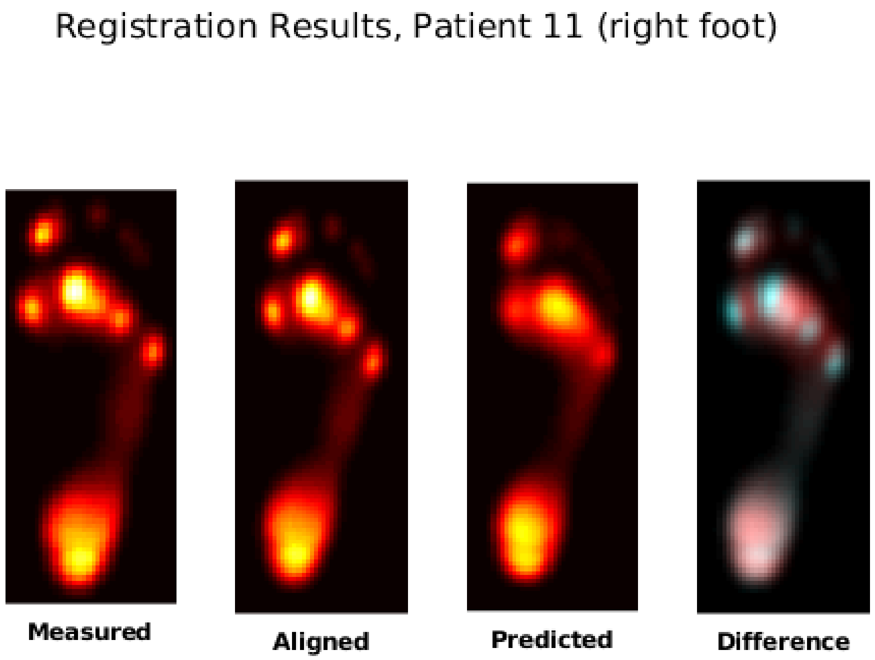
Registration Results, Patient 11 (right foot)
Measured
Aligned
Predicted
Difference

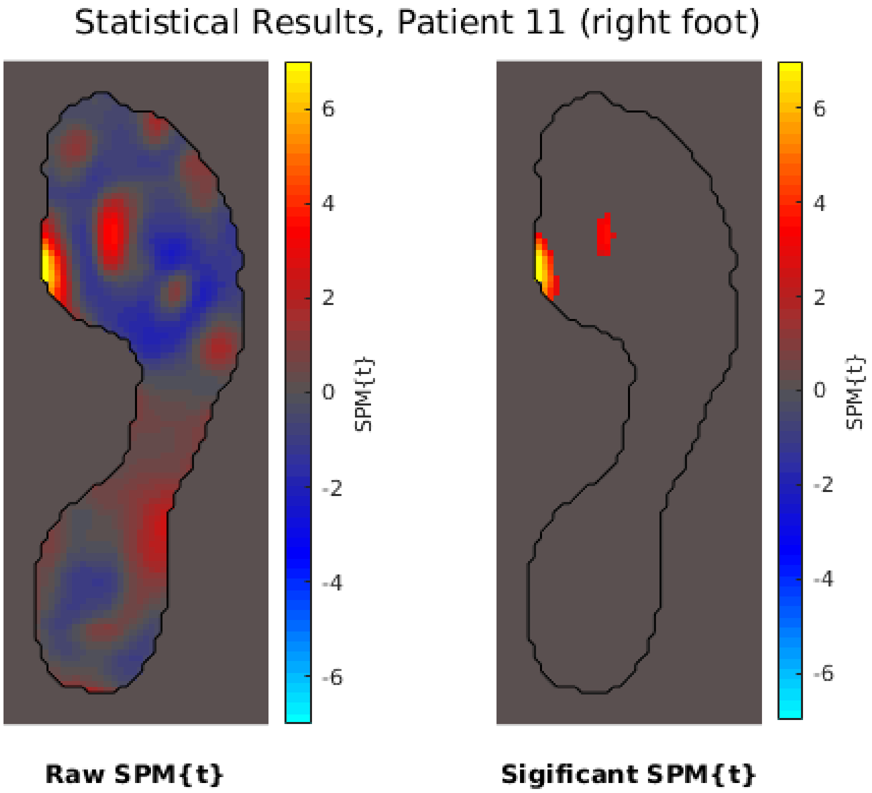
Statistical Results, Patient 11 (right foot)
6
4
2
0
-2
-4
-6
SPM{t}
Raw SPM{t}
Sigificant SPM{t}

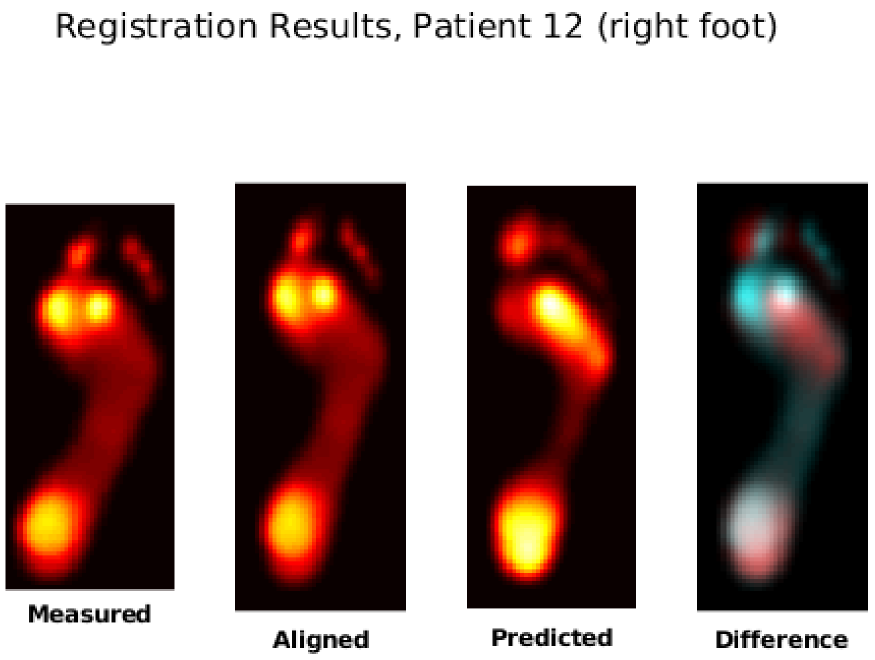
Registration Results, Patient 12 (right foot)
Measured
Aligned
Predicted
Difference

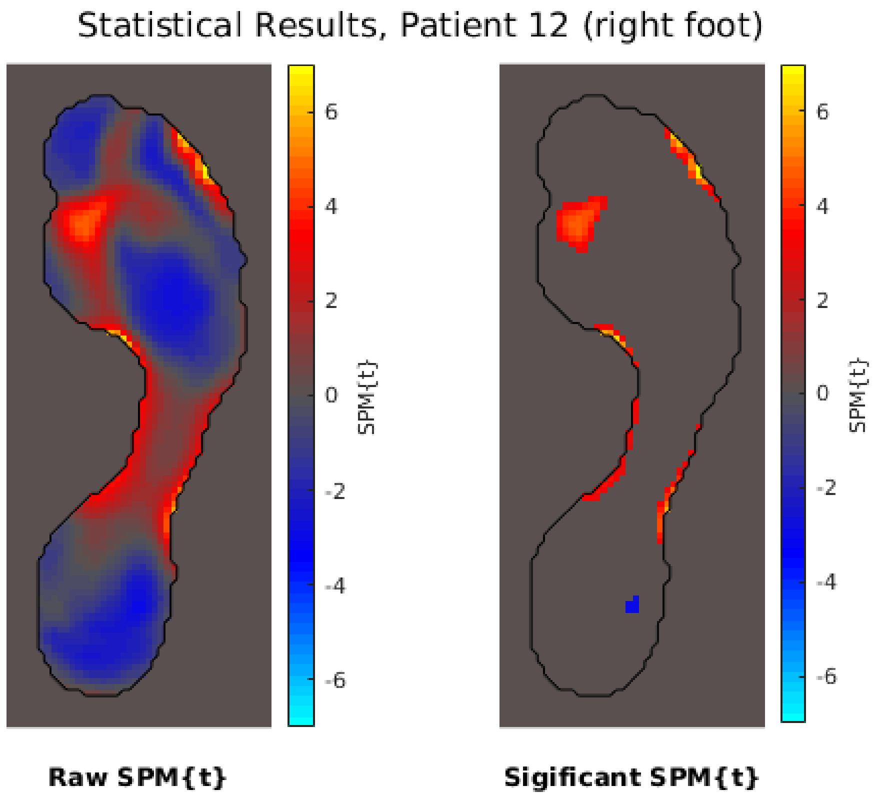
Statistical Results, Patient 12 (right foot)
6
4
2
0
-2
-4
-6
SPM{t}
Raw SPM{t}
Sigificant SPM{t}

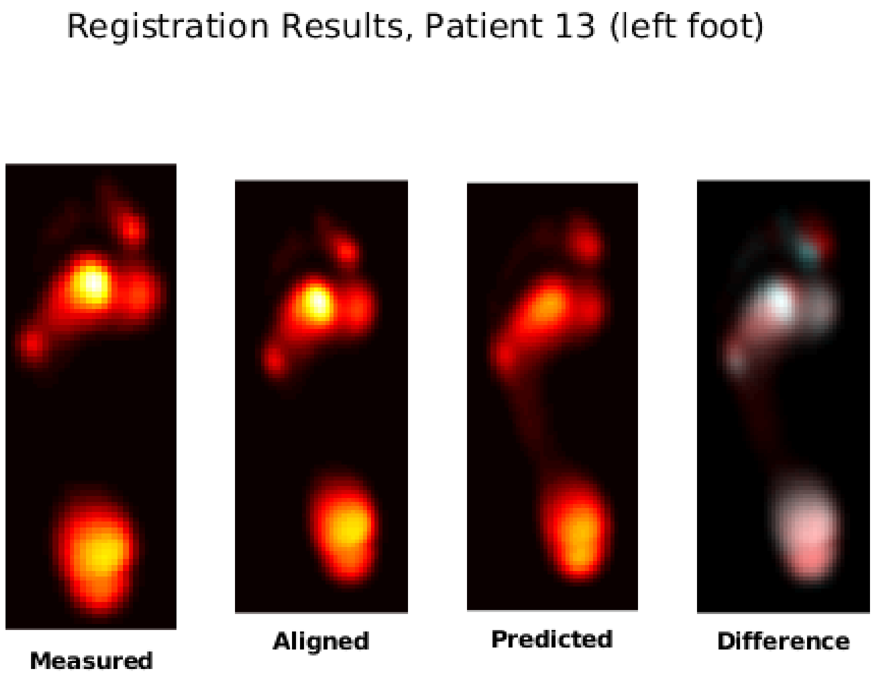
Registration Results, Patient 13 (left foot)
Measured
Aligned
Predicted
Difference

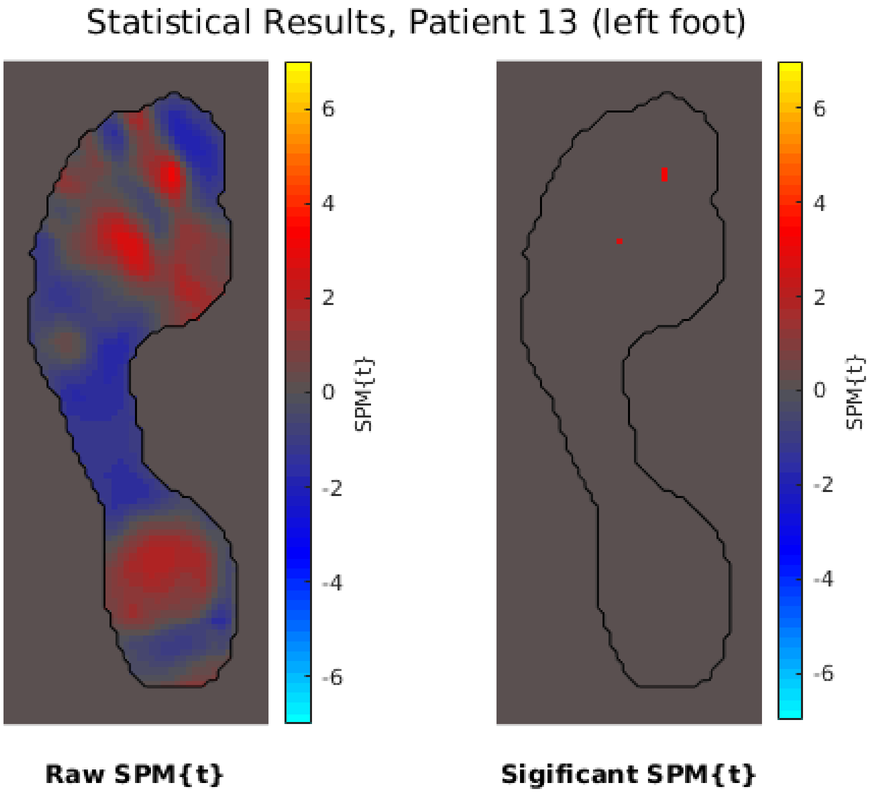
Statistical Results, Patient 13 (left foot)
6
4
2
0
-2
-4
-6
SPM{t}
Raw SPM{t}
Sigificant SPM{t}

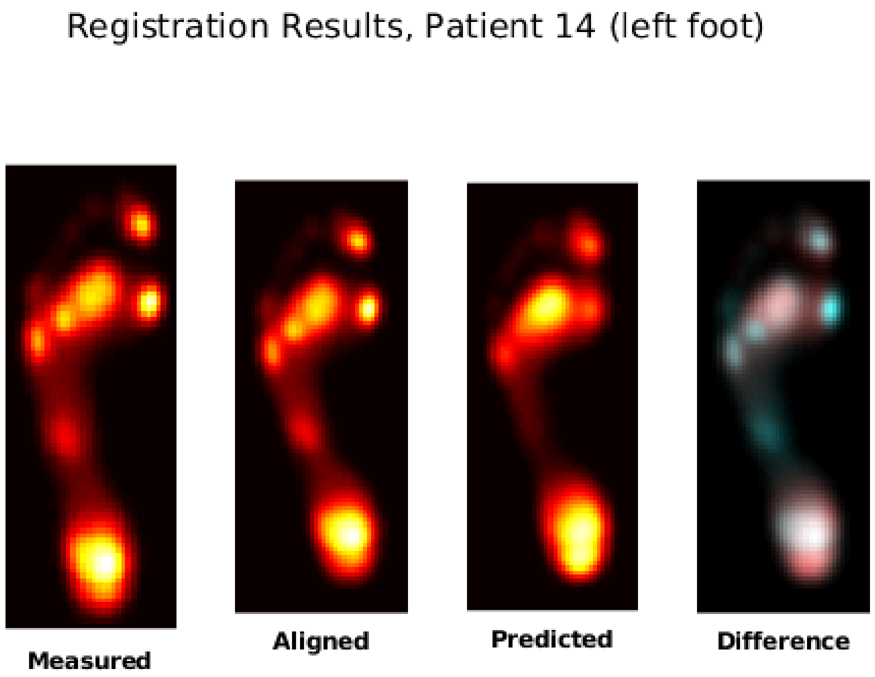
Registration Results, Patient 14 (left foot)
Measured
Aligned
Predicted
Difference

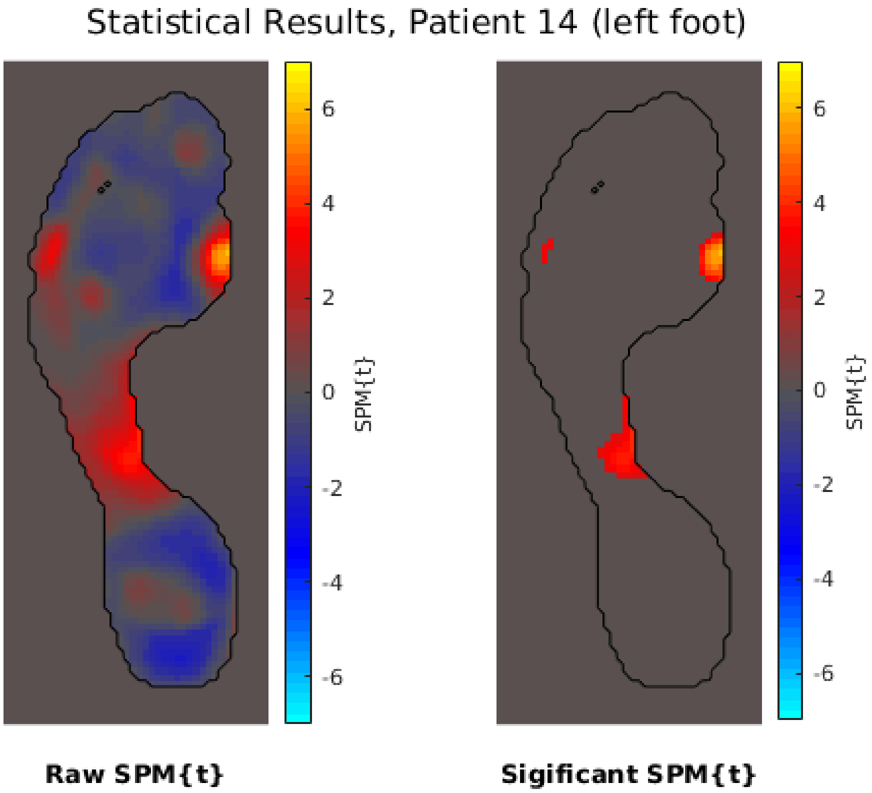
Statistical Results, Patient 14 (left foot)
6
4
2
0
-2
-4
-6
SPM{t}
Raw SPM{t}
Sigificant SPM{t}

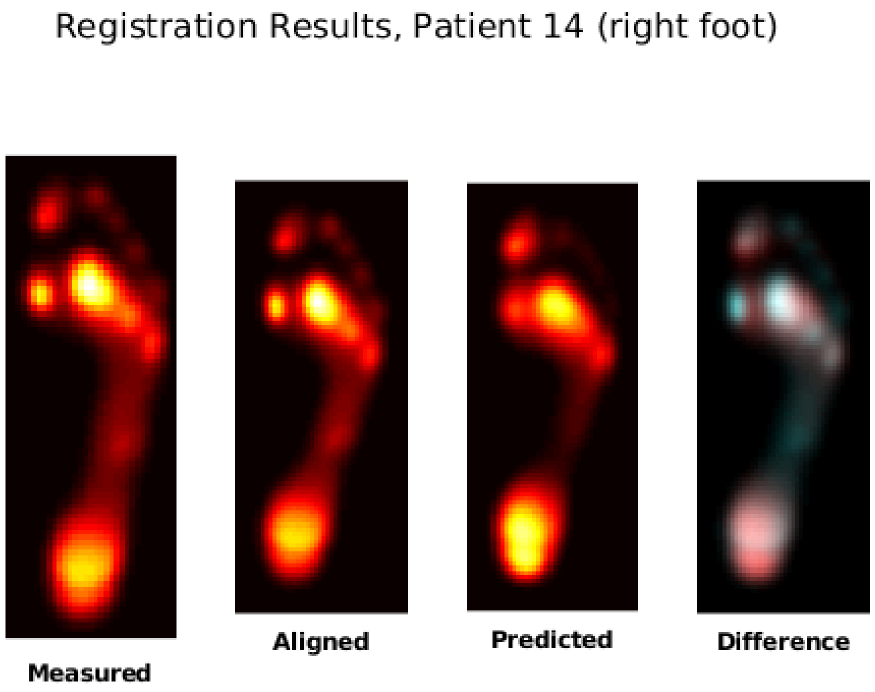
Registration Results, Patient 14 (right foot)
Measured
Aligned
Predicted
Difference

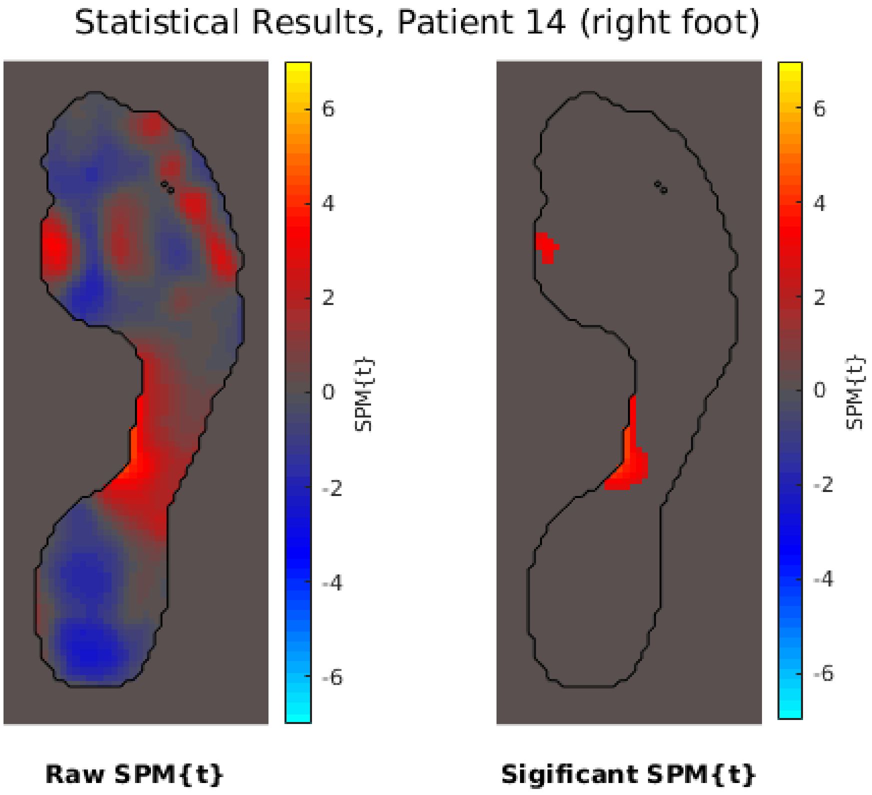
Statistical Results, Patient 14 (right foot)
6
4
2
0
-2
-4
-6
SPM{t}
Raw SPM{t}
Sigificant SPM{t}

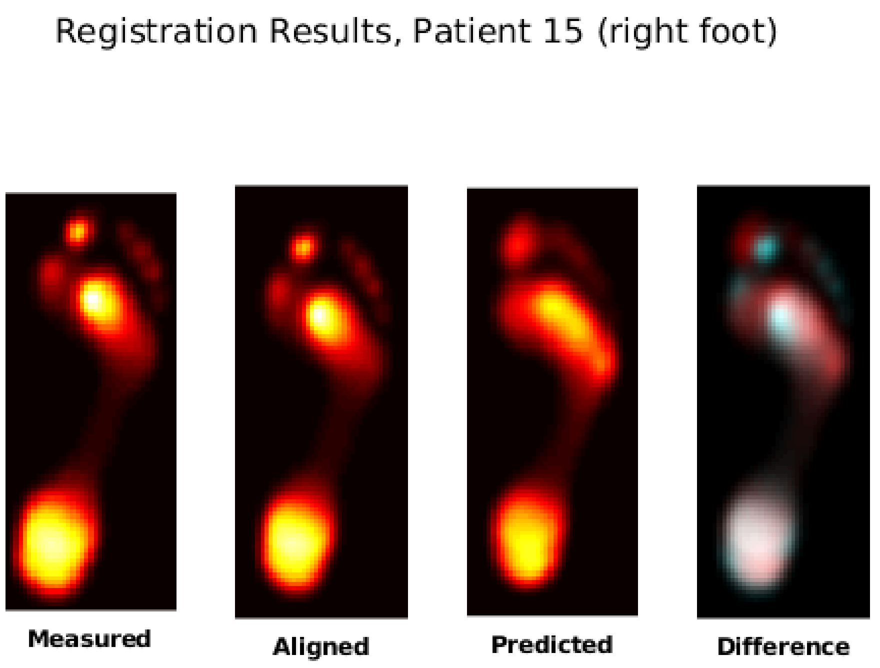

Registration Results, Patient 15 (right foot)
Measured
Aligned
Predicted
Difference

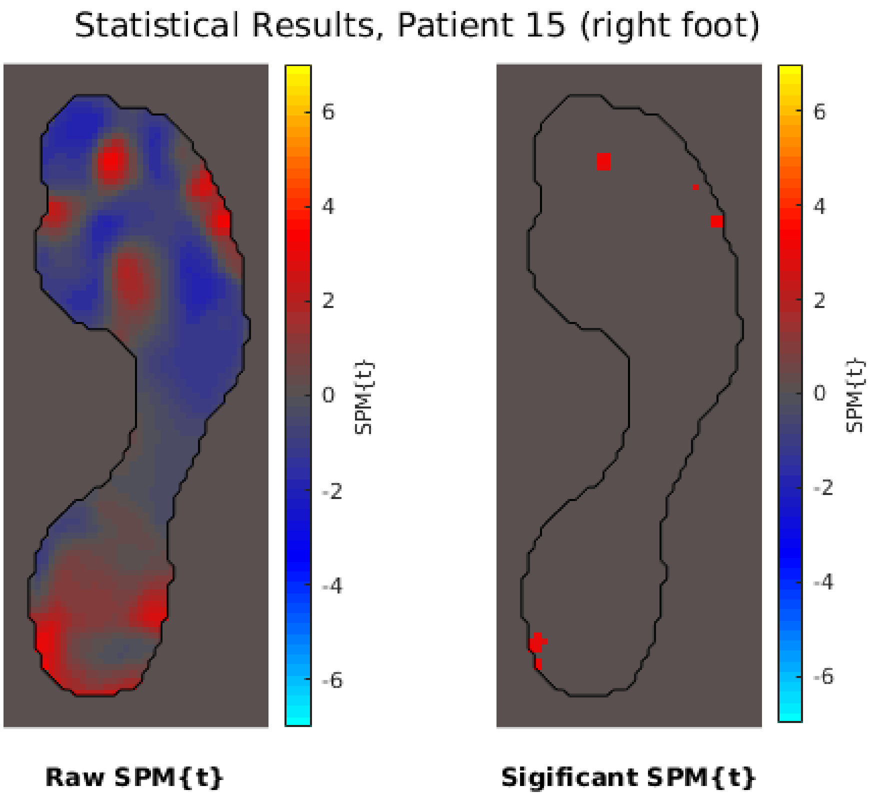

Statistical Results, Patient 15 (right foot)
6
4
2
0
-2
-4
-6
SPM{t}
Raw SPM{t}
Sigificant SPM{t}

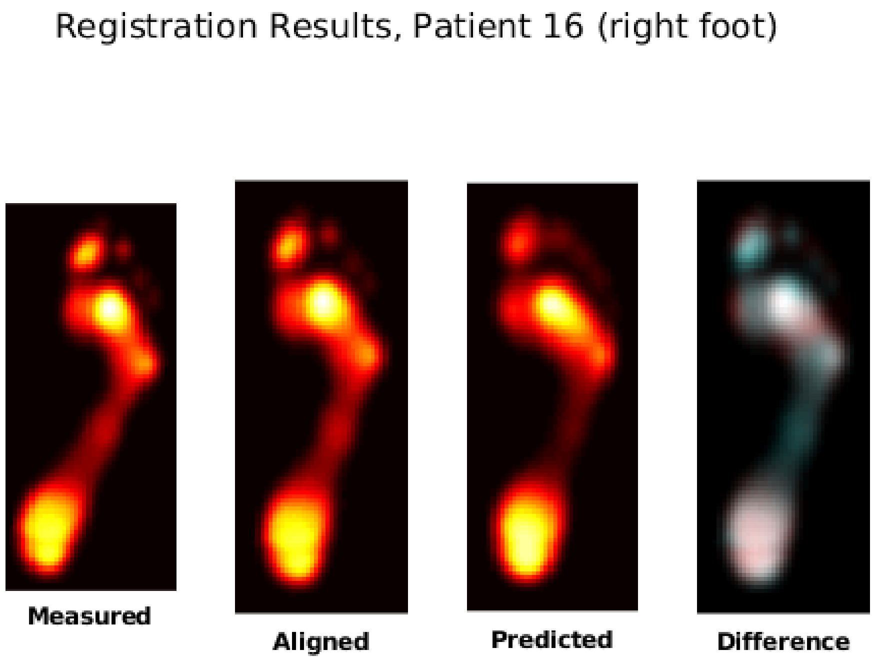
Registration Results, Patient 16 (right foot)
Measured
Aligned
Predicted
Difference

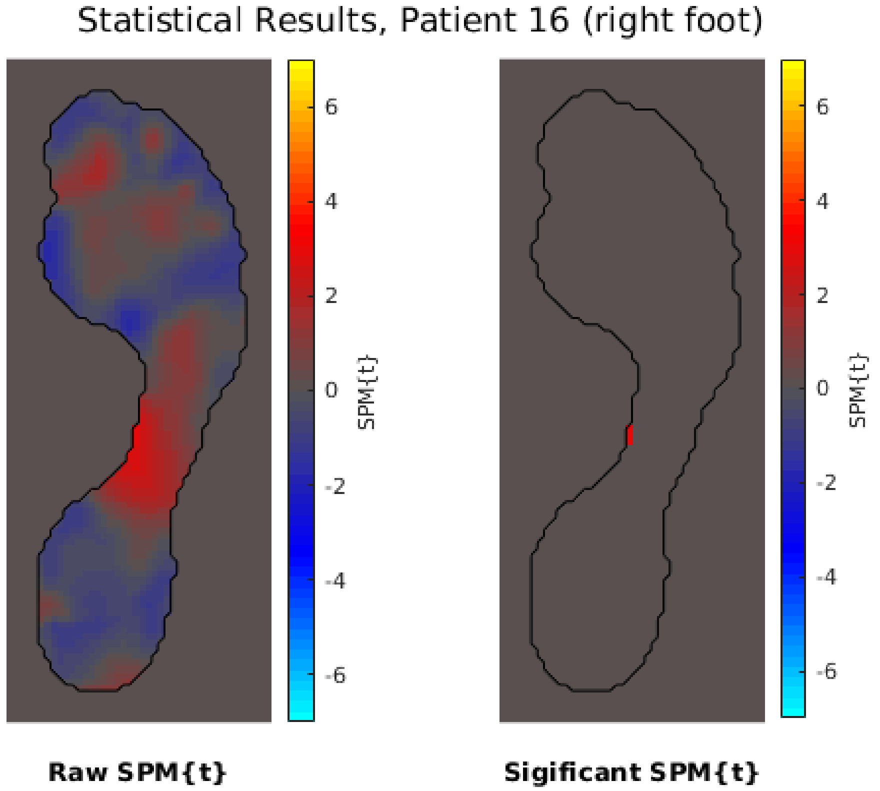
Statistical Results, Patient 16 (right foot)
6
4
2
0
-2
-4
-6
SPM{t}
Raw SPM{t}
Sigificant SPM{t}

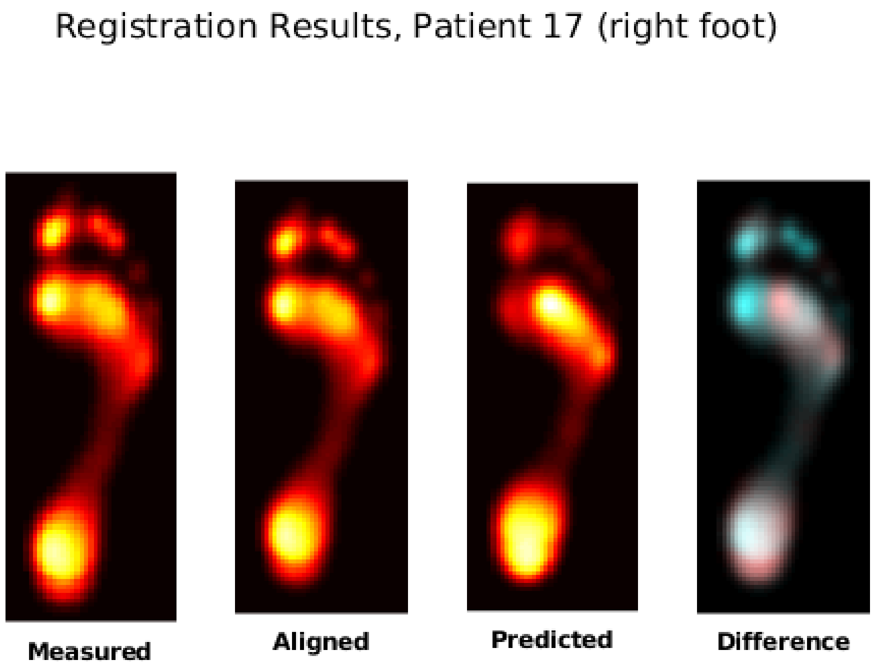
Registration Results, Patient 17 (right foot)
Measured
Aligned
Predicted
Difference

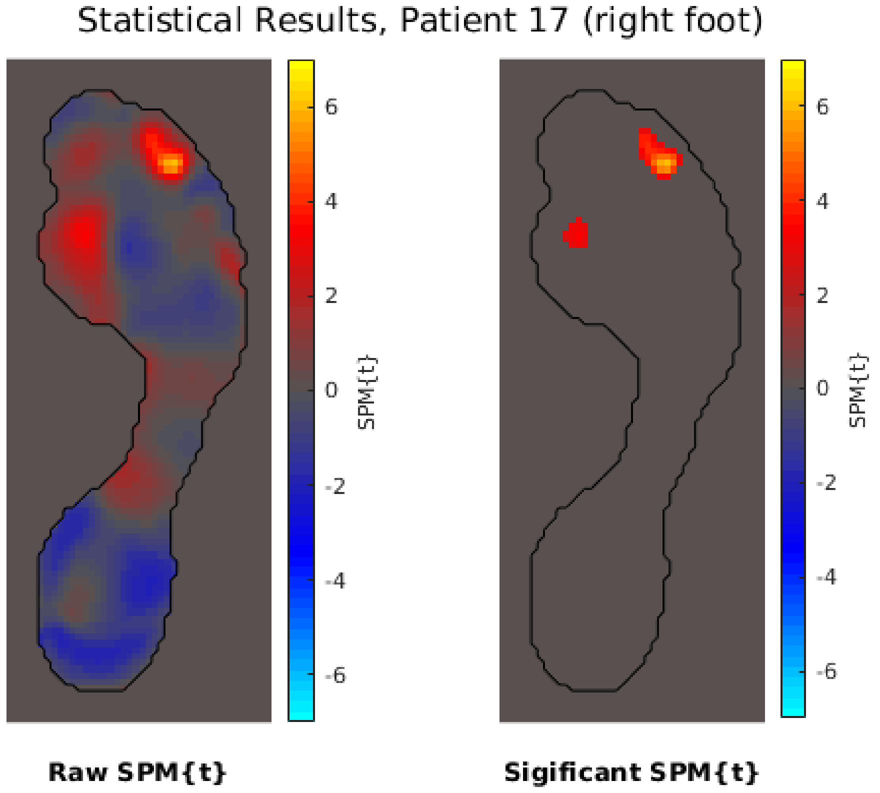
Statistical Results, Patient 17 (right foot)
6
4
2
0
-2
-4
-6
SPM{t}
Raw SPM{t}
Sigificant SPM{t}

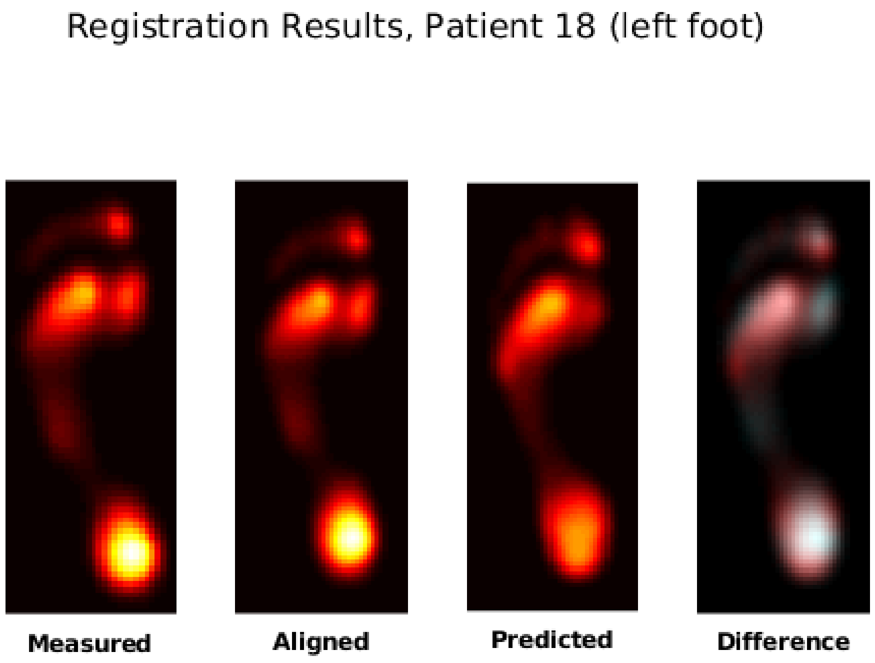
Registration Results, Patient 18 (left foot)
Measured
Aligned
Predicted
Difference

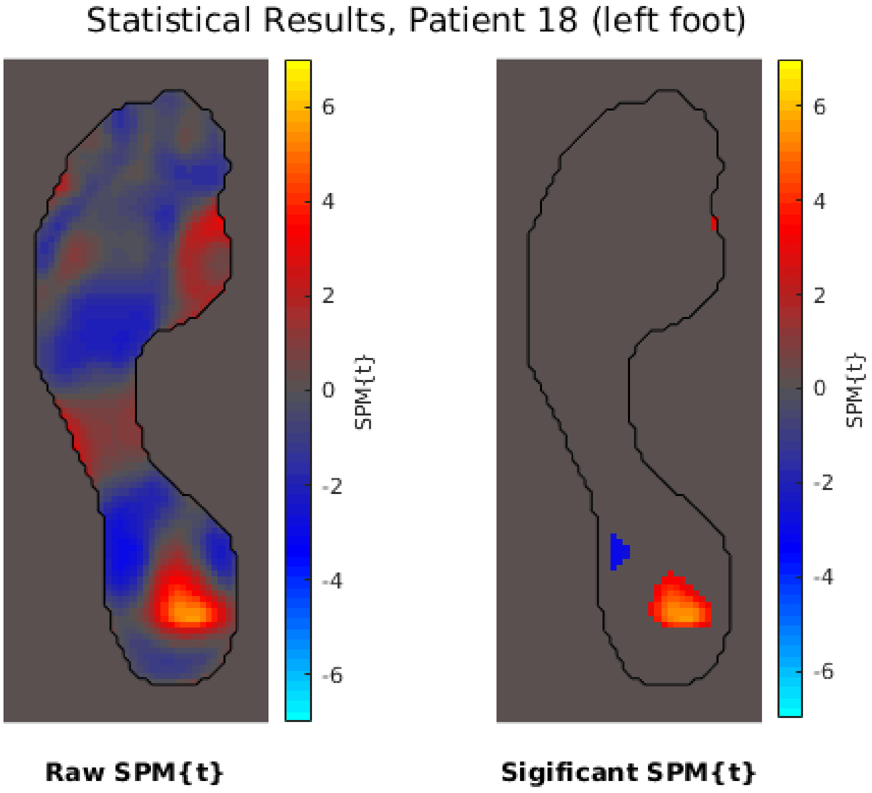
Statistical Results, Patient 18 (left foot)
6
4
2
0
-2
-4
-6
SPM{t}
Raw SPM{t}
Sigificant SPM{t}

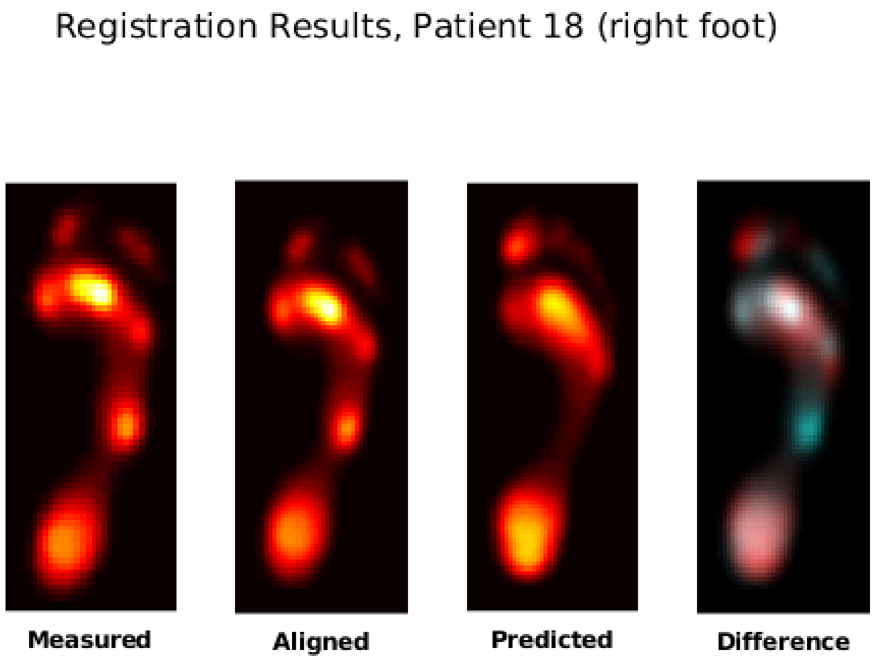
Registration Results, Patient 18 (right foot)
Measured
Aligned
Predicted
Difference

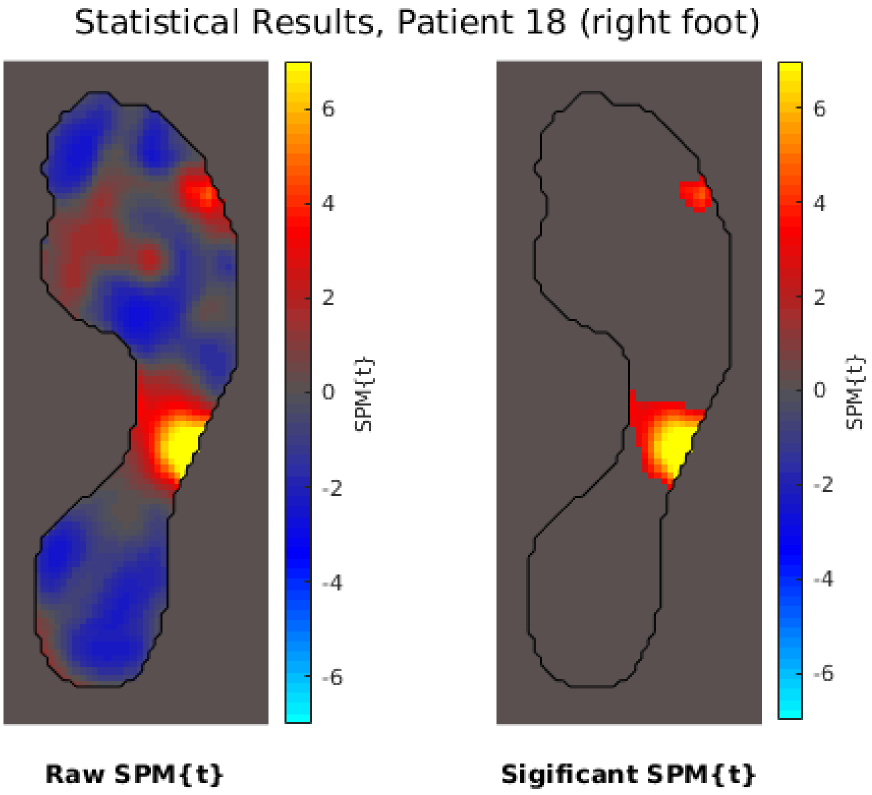
Statistical Results, Patient 18 (right foot)
6
4
2
0
-2
-4
-6
SPM{t}
Raw SPM{t}
Sigificant SPM{t}

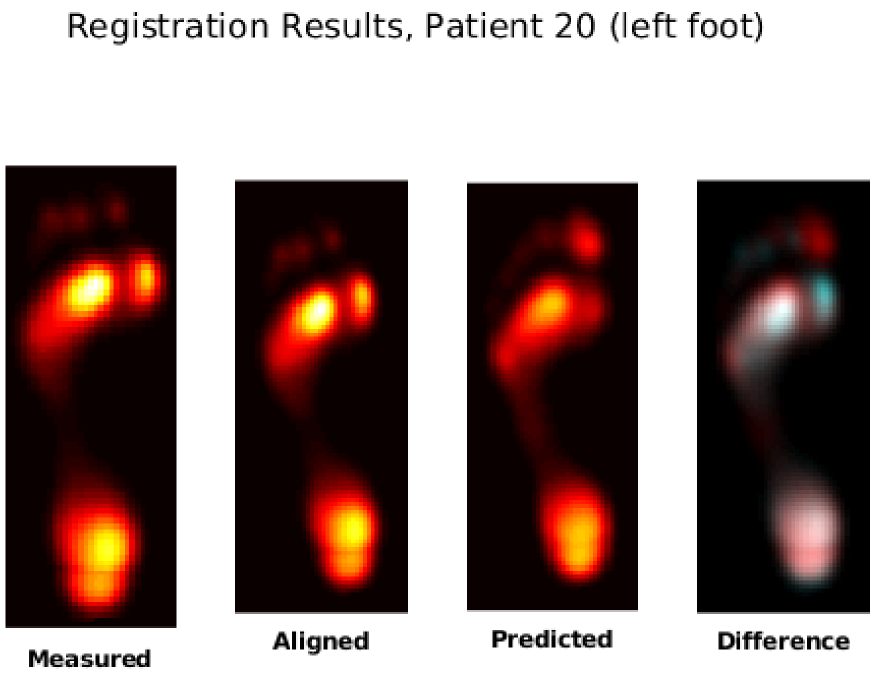
Registration Results, Patient 20 (left foot)
Measured
Aligned
Predicted
Difference

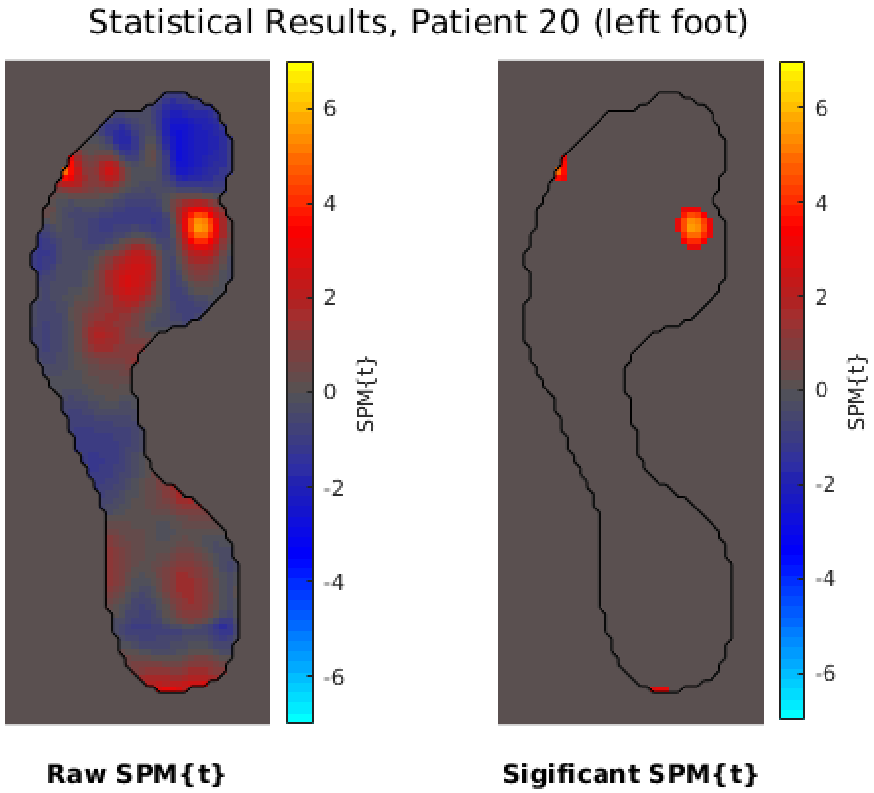
Statistical Results, Patient 20 (left foot)
6
4
2
0
-2
-4
-6
SPM{t}
Raw SPM{t}
Sigificant SPM{t}

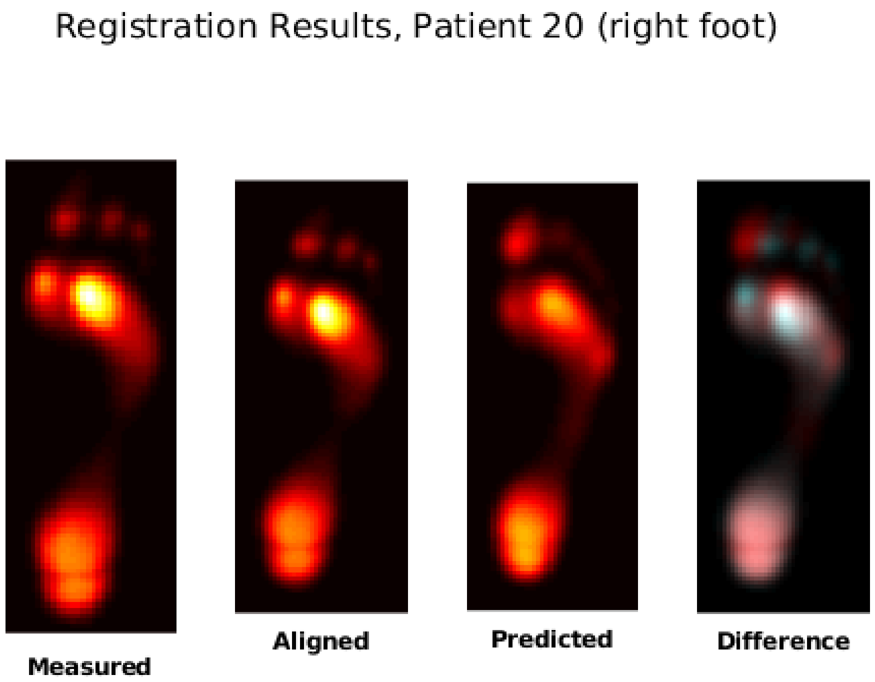
Registration Results, Patient 20 (right foot)
Measured
Aligned
Predicted
Difference

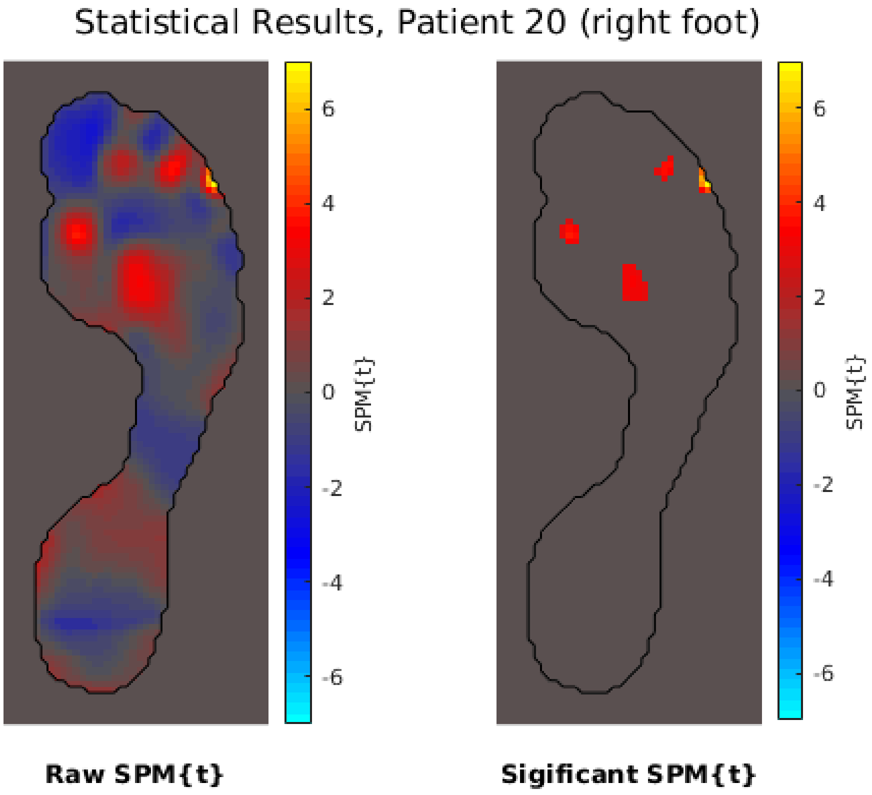
Statistical Results, Patient 20 (right foot)
6
4
2
0
-2
-4
-6
SPM{t}
Raw SPM{t}
Sigificant SPM{t}

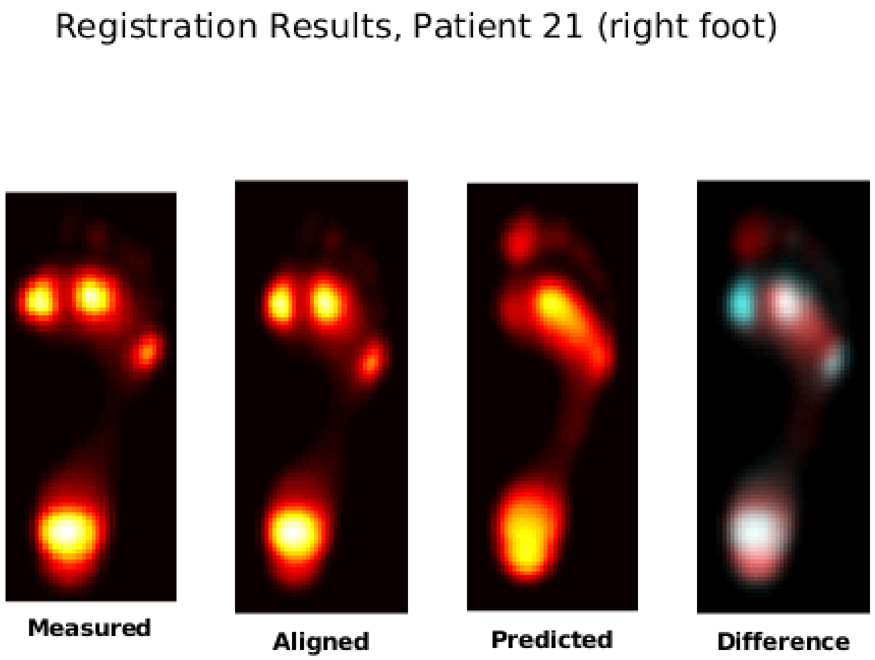

Registration Results, Patient 21 (right foot)
Measured
Aligned
Predicted
Difference

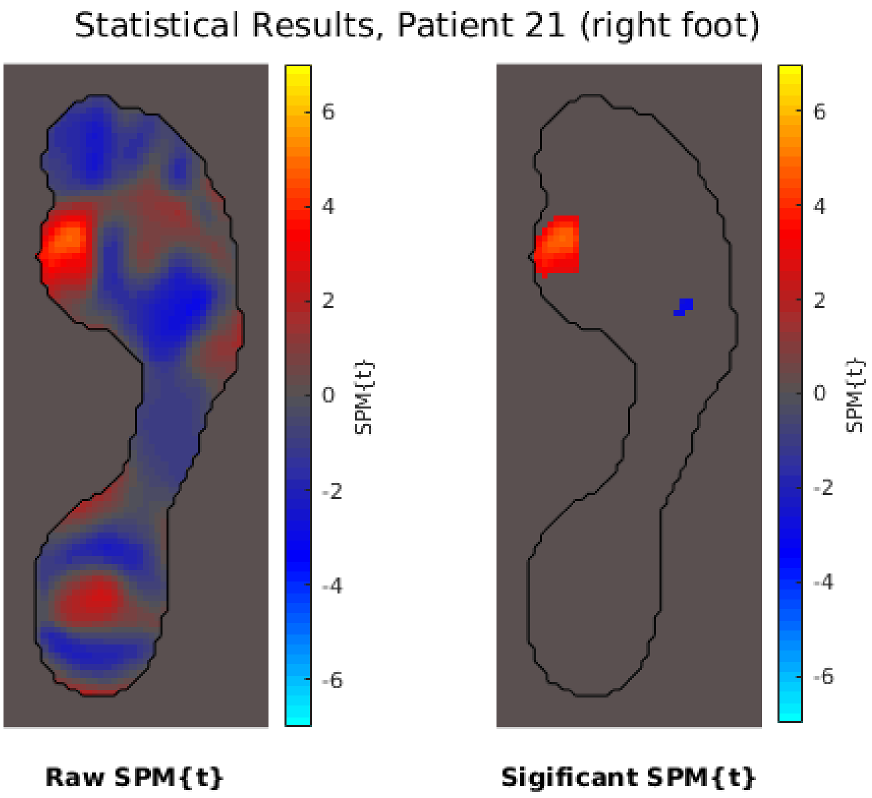

Statistical Results, Patient 21 (right foot)
6
4
2
0
-2
-4
-6
SPM{t}
Raw SPM{t}
Sigificant SPM{t}

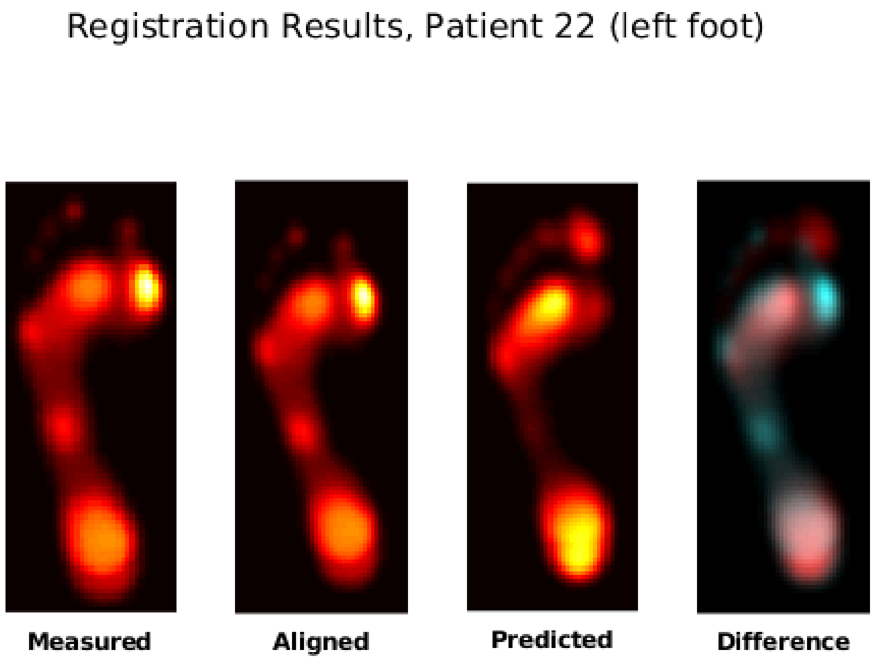
Registration Results, Patient 22 (left foot)
Measured
Aligned
Predicted
Difference

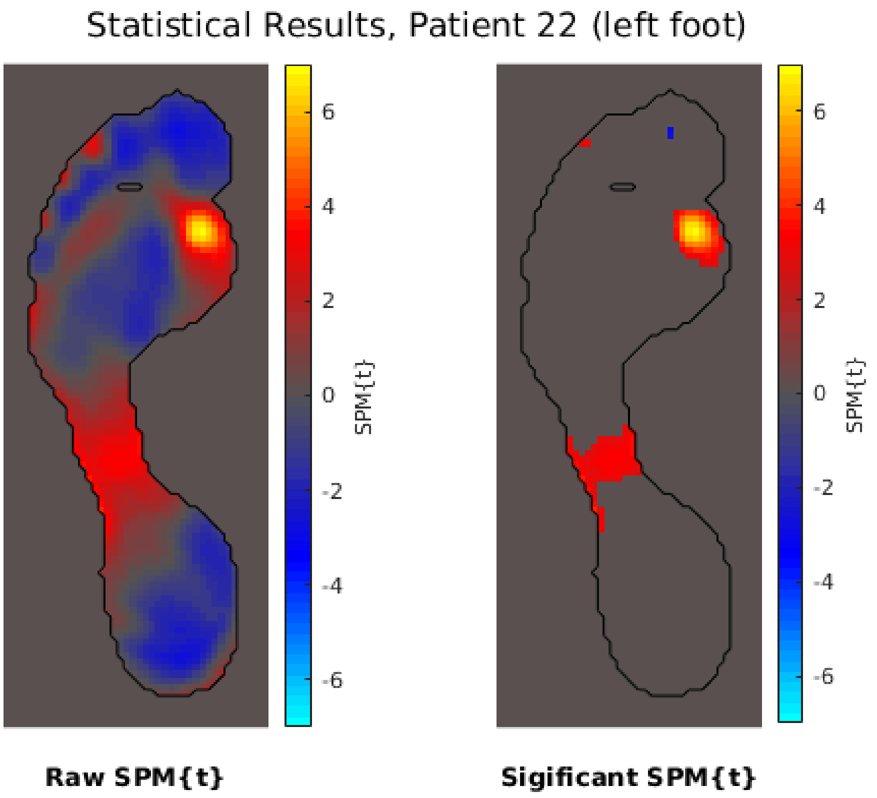
Statistical Results, Patient 22 (left foot)
6
4
2
0
-2
-4
-6
SPM{t}
Raw SPM{t}
Sigificant SPM{t}

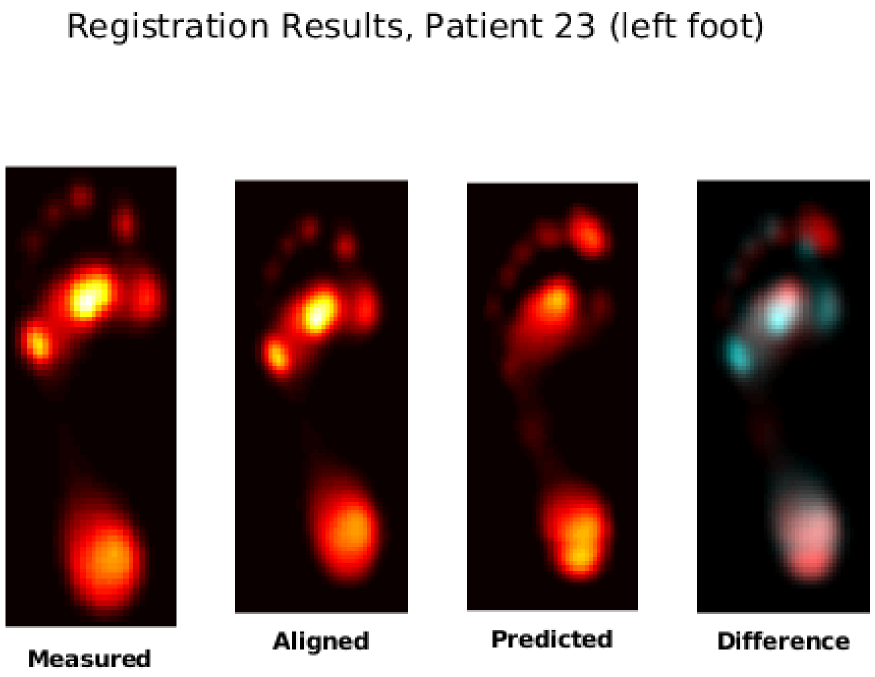
Registration Results, Patient 23 (left foot)
Measured
Aligned
Predicted
Difference

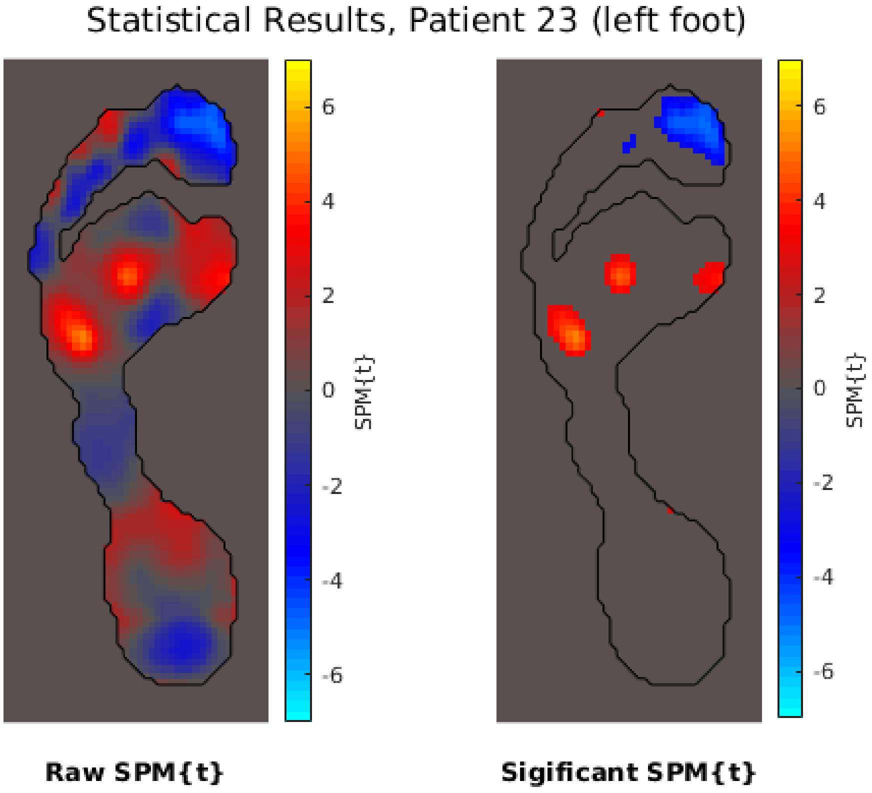
Statistical Results, Patient 23 (left foot)
6
4
2
0
-2
-4
-6
SPM{t}
Raw SPM{t}
Sigificant SPM{t}

Registration Results, Patient 24 (left foot)

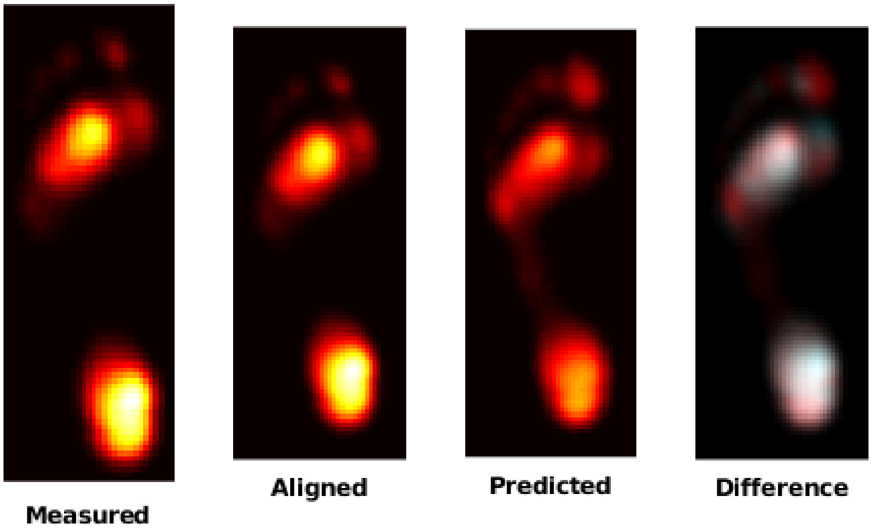

Statistical Results, Patient 24 (left foot)

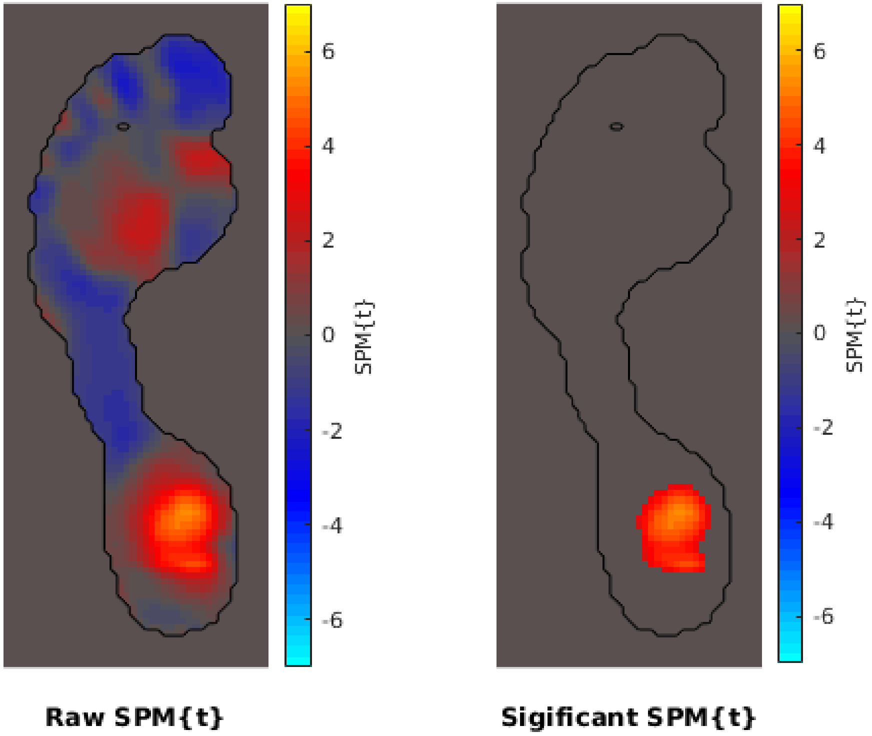

Registration Results, Patient 24 (right foot)

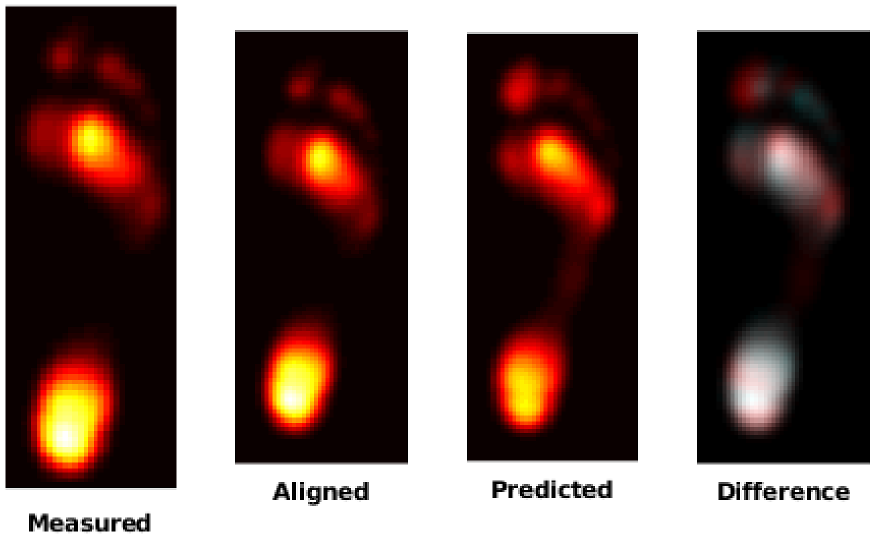

Statistical Results, Patient 24 (right foot)

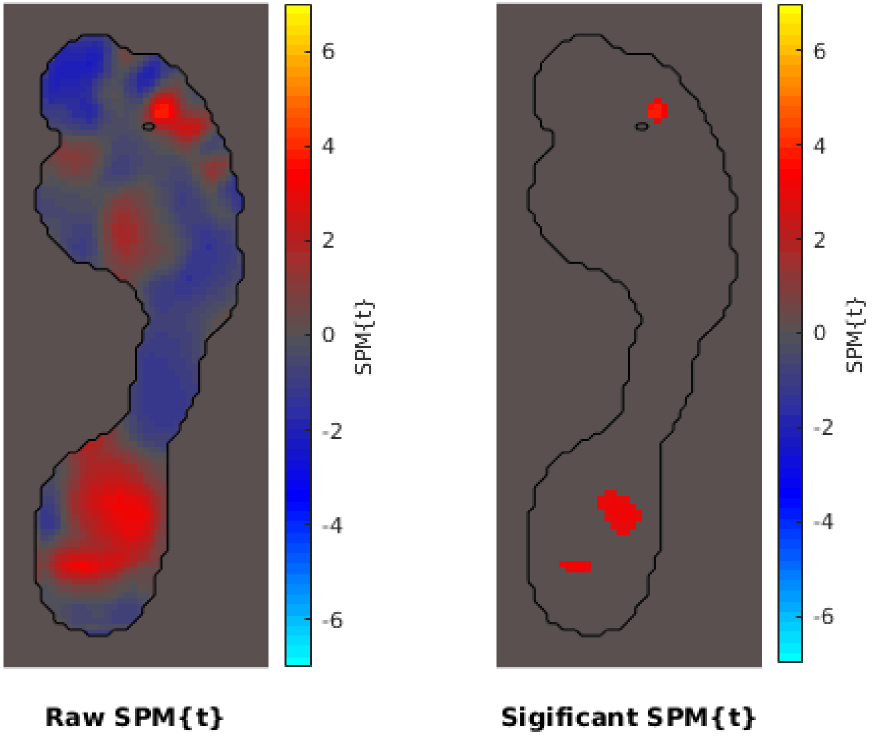

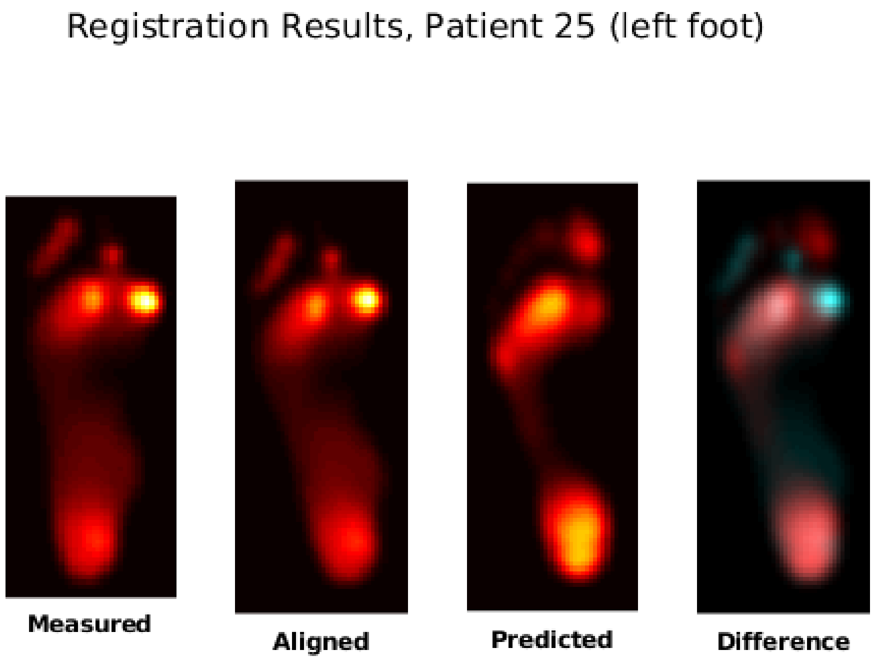
Registration Results, Patient 25 (left foot)
Measured
Aligned
Predicted
Difference

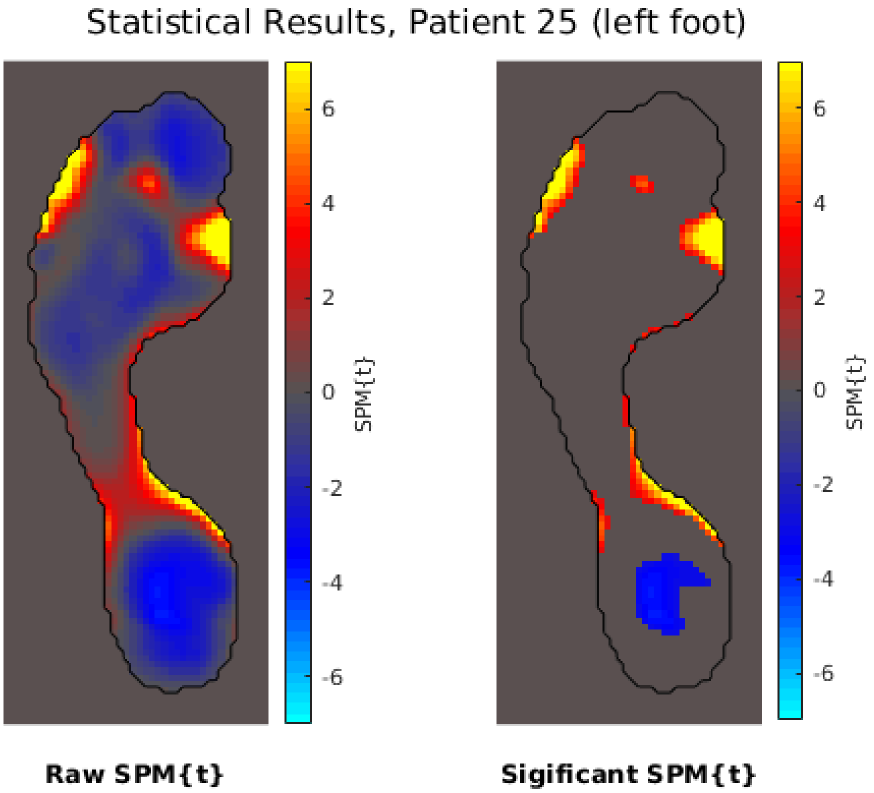
Statistical Results, Patient 25 (left foot)
6
4
2
0
-2
-4
-6
SPM{t}
6
4
2
0
-2
-4
-6
SPM{t}
Raw SPM{t}
Sigificant SPM{t}

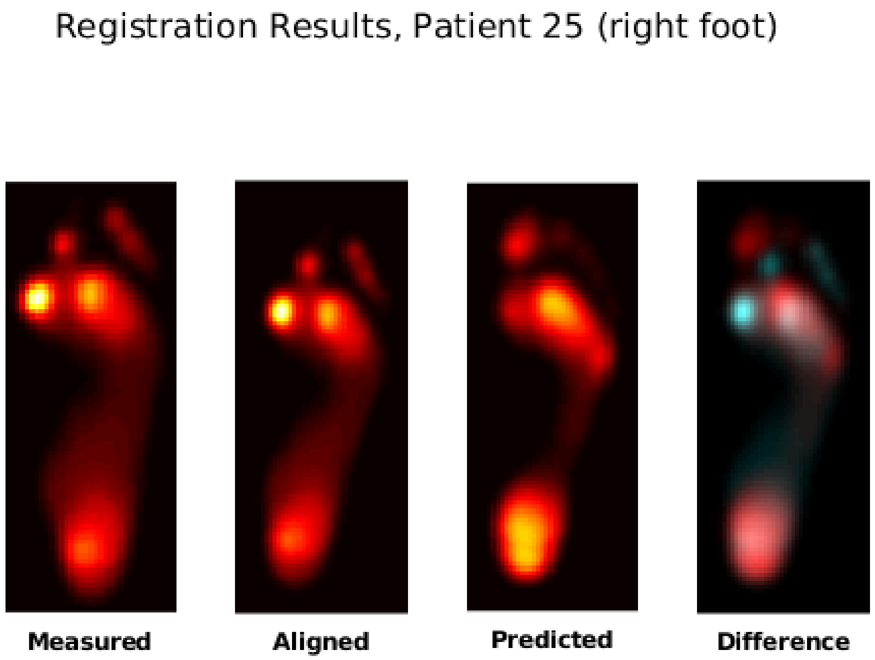
Registration Results, Patient 25 (right foot)
Measured
Aligned
Predicted
Difference

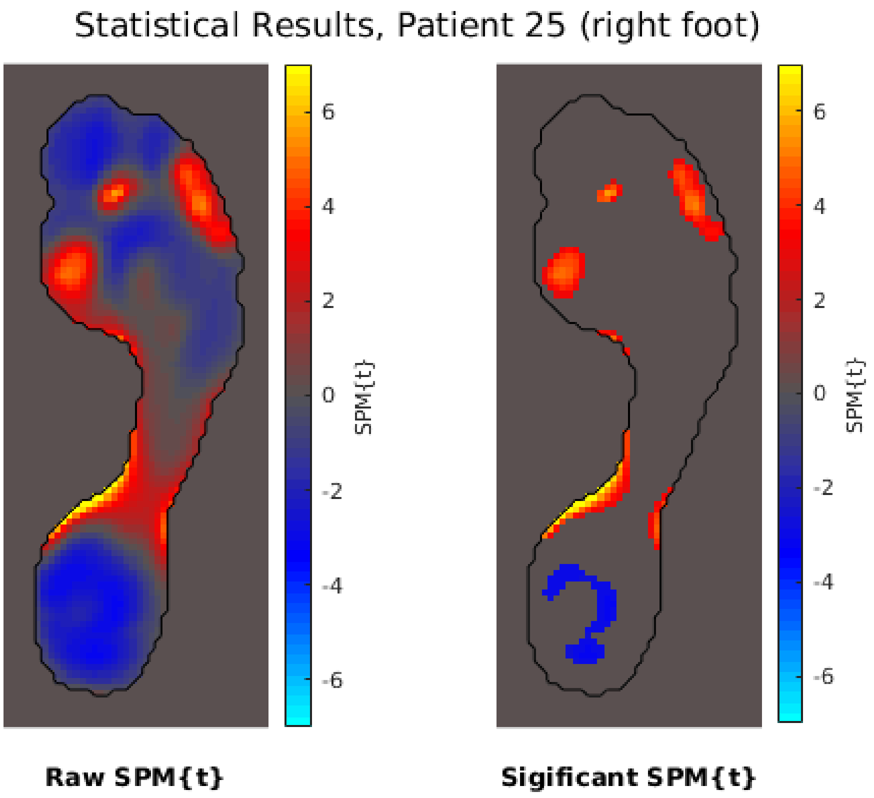
Statistical Results, Patient 25 (right foot)
6
4
2
0
-2
-4
-6
SPM{t}
Raw SPM{t}
Sigificant SPM{t}

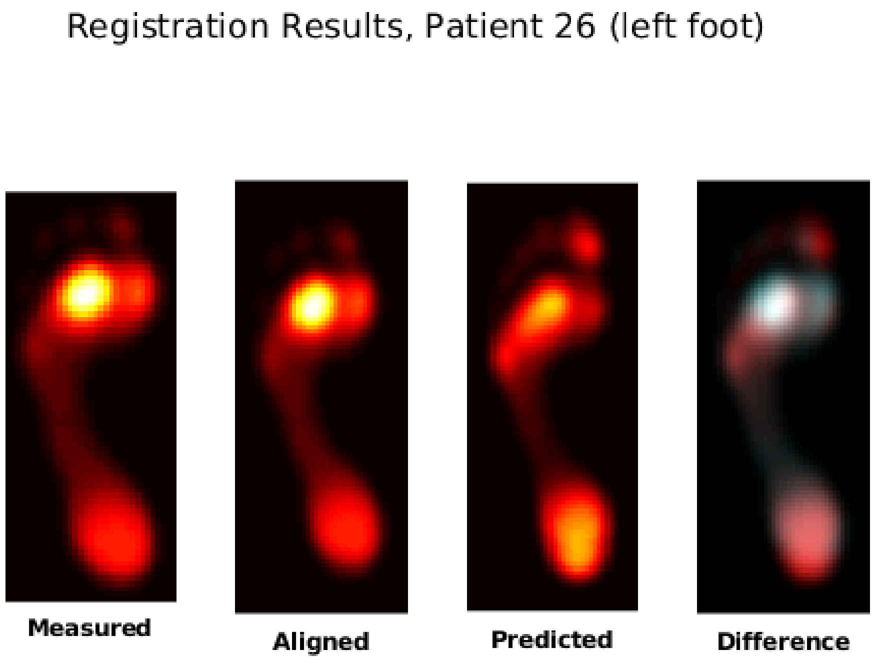
Registration Results, Patient 26 (left foot)
Measured
Aligned
Predicted
Difference

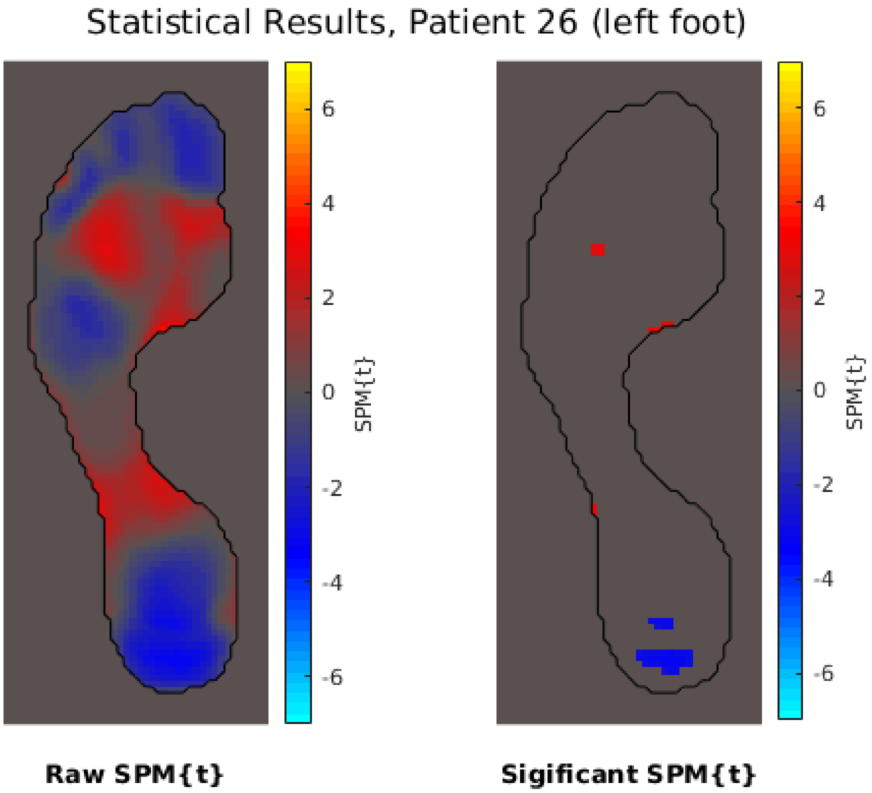
Statistical Results, Patient 26 (left foot)
6
4
2
0
-2
-4
-6
SPM{t}
Raw SPM{t}
Sigificant SPM{t}

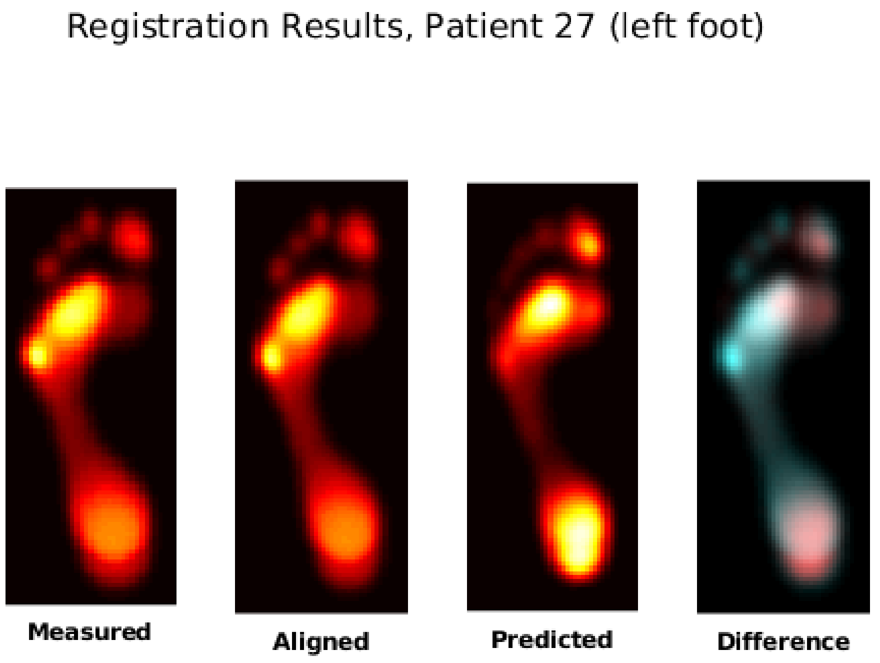
Registration Results, Patient 27 (left foot)
Measured
Aligned
Predicted
Difference

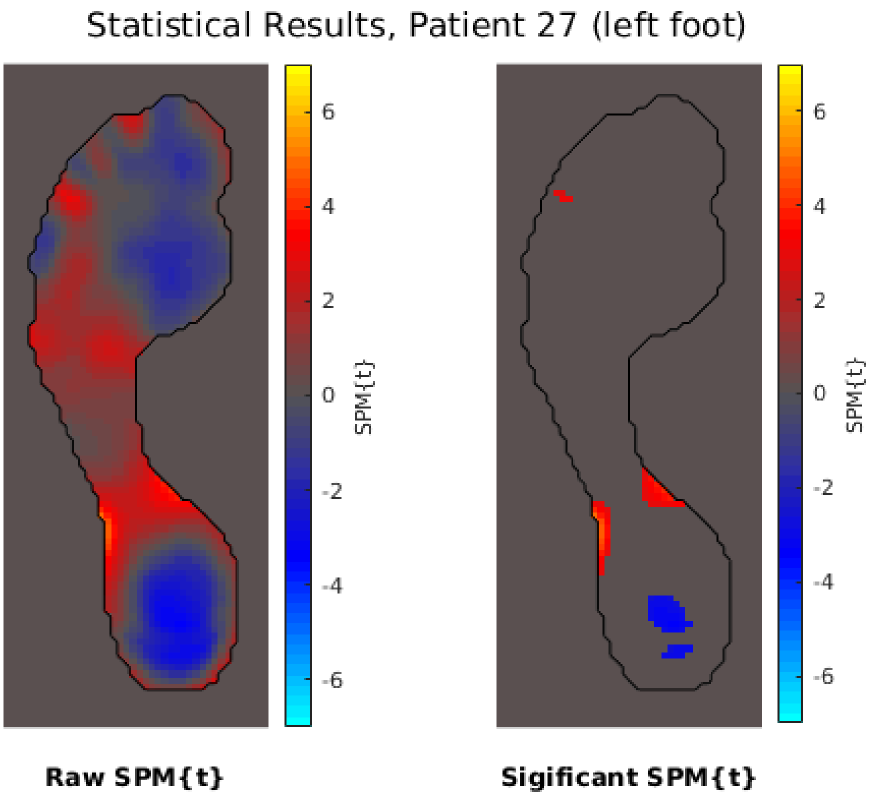
Statistical Results, Patient 27 (left foot)
6
4
2
0
-2
-4
-6
SPM{t}
Raw SPM{t}
Sigificant SPM{t}

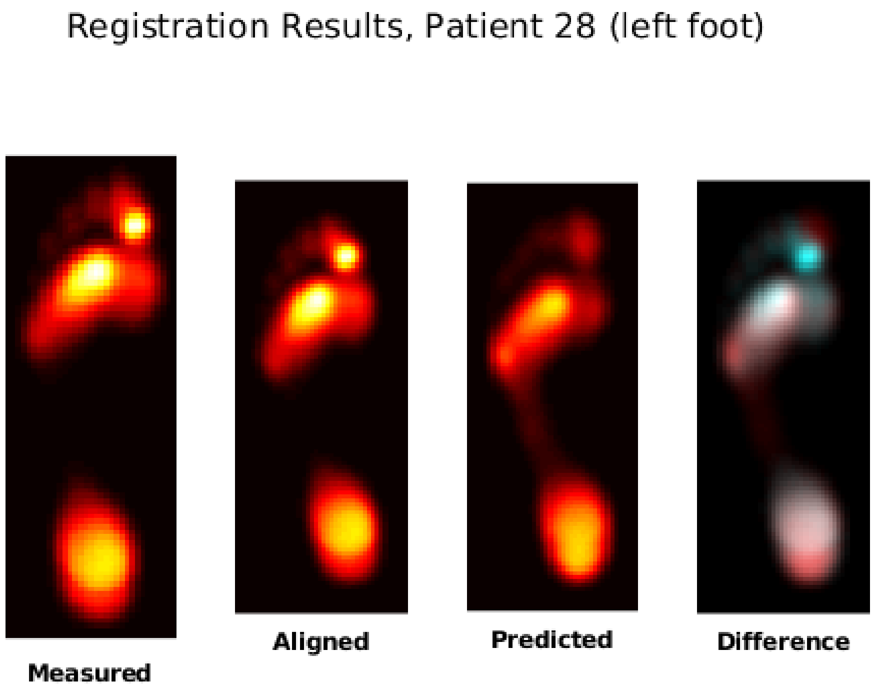
Registration Results, Patient 28 (left foot)
Measured
Aligned
Predicted
Difference

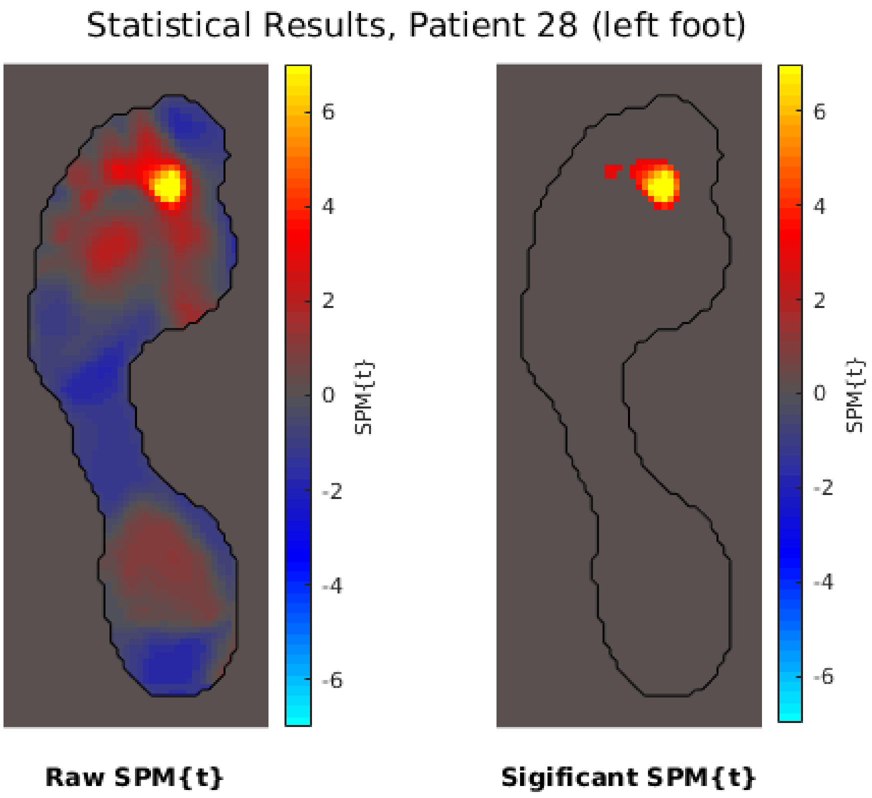
Statistical Results, Patient 28 (left foot)
6
4
2
0
-2
-4
-6
SPM{t}
Raw SPM{t}
Sigificant SPM{t}

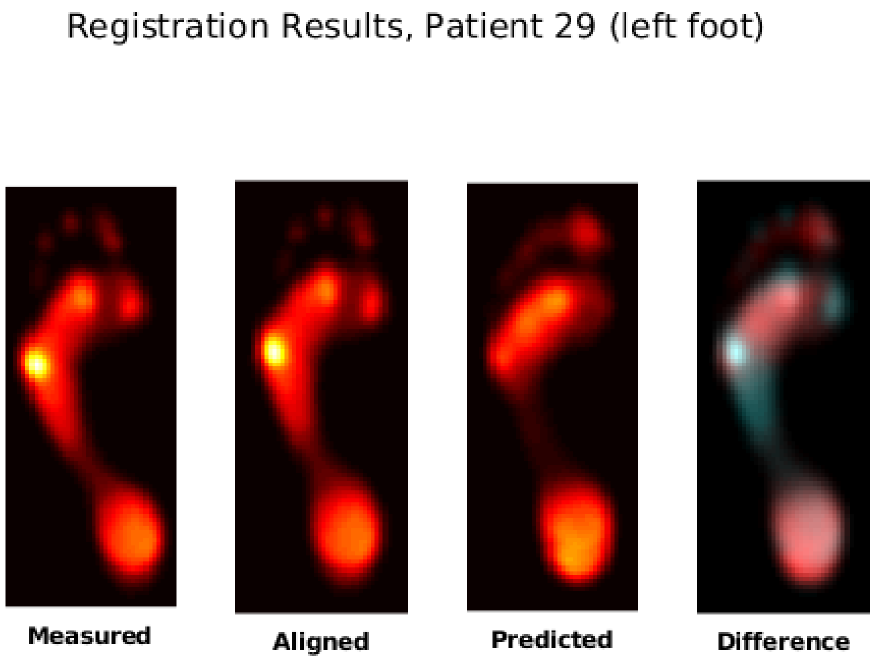
Registration Results, Patient 29 (left foot)
Measured
Aligned
Predicted
Difference

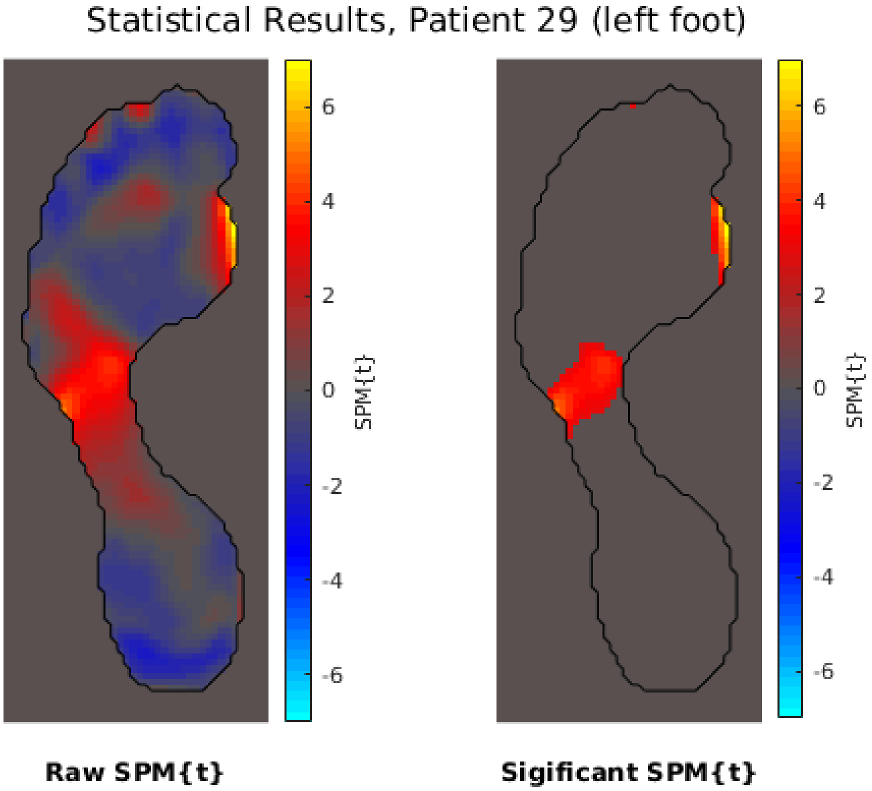
Statistical Results, Patient 29 (left foot)
6
4
2
0
-2
-4
-6
SPM{t}
Raw SPM{t}
Sigificant SPM{t}

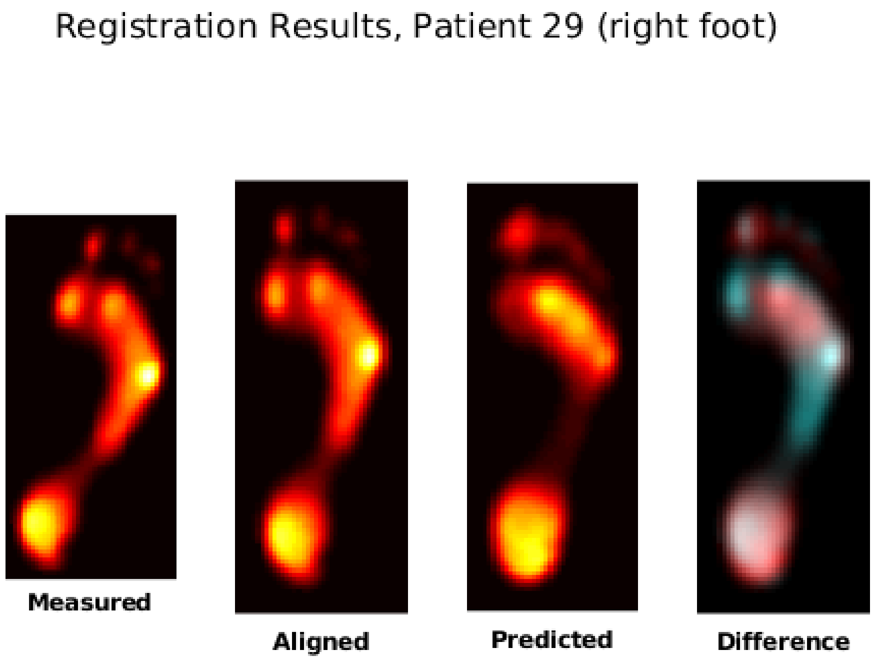
Registration Results, Patient 29 (right foot)
Measured
Aligned
Predicted
Difference

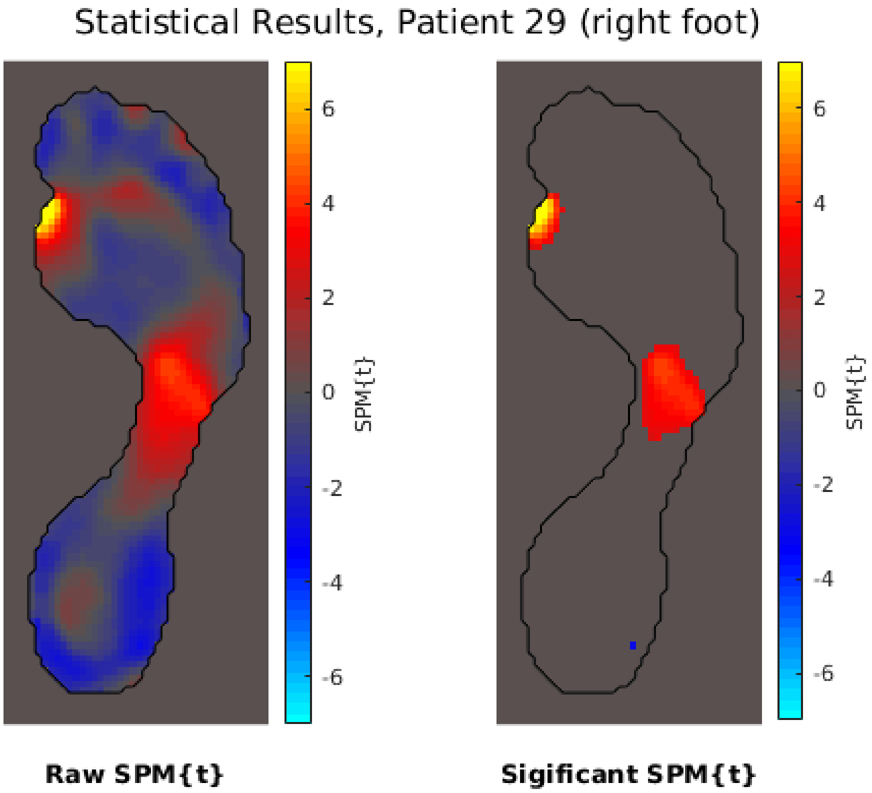
Statistical Results, Patient 29 (right foot)
6
4
2
0
-2
-4
-6
SPM{t}
Raw SPM{t}
Sigificant SPM{t}

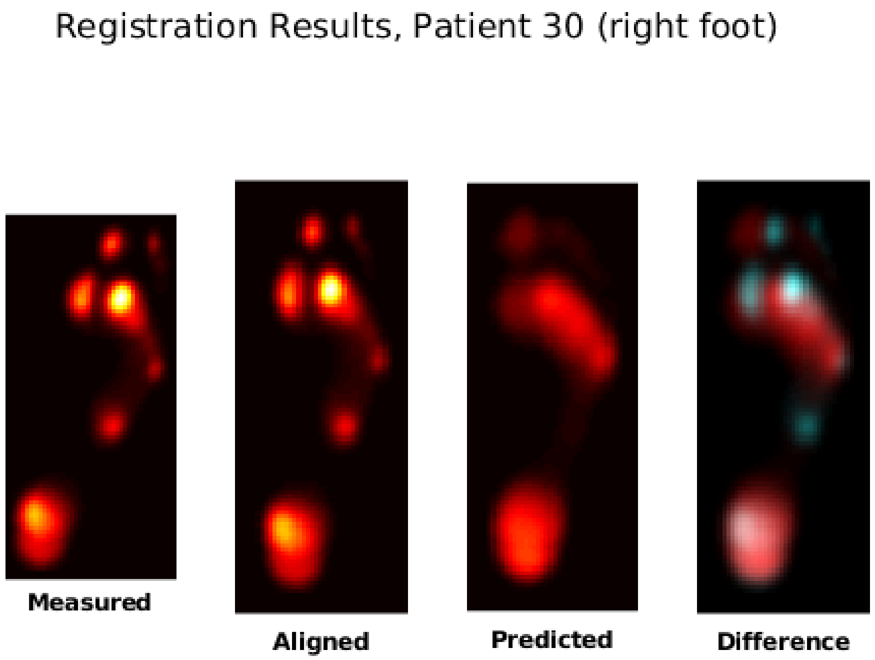
Registration Results, Patient 30 (right foot)
Measured
Aligned
Predicted
Difference

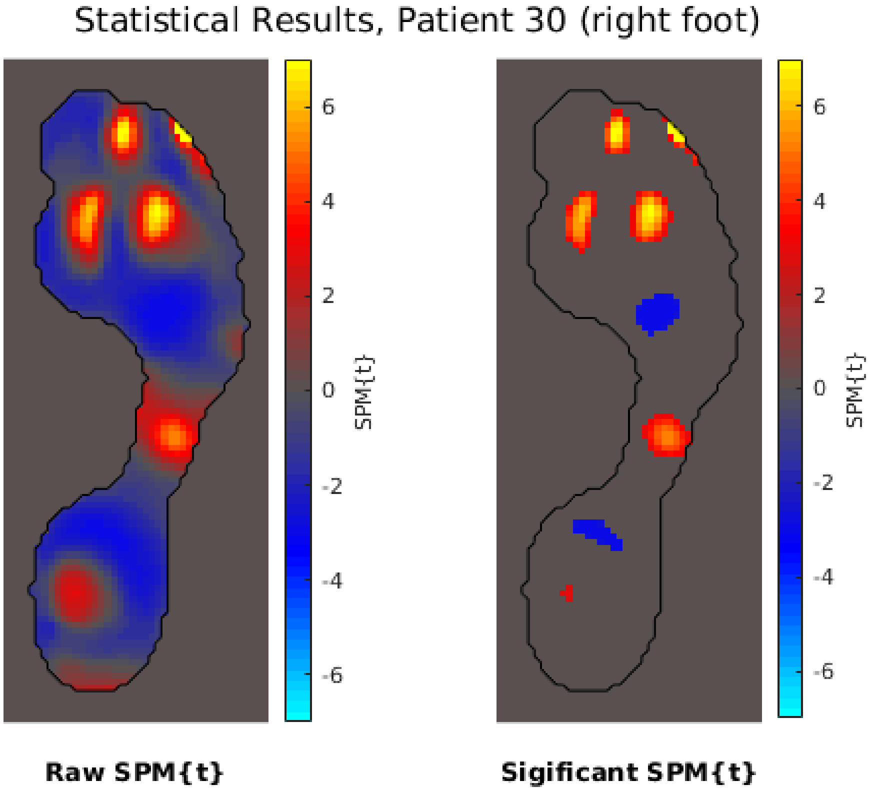
Statistical Results, Patient 30 (right foot)
6
4
2
0
-2
-4
-6
SPM{t}
Raw SPM{t}
Sigificant SPM{t}

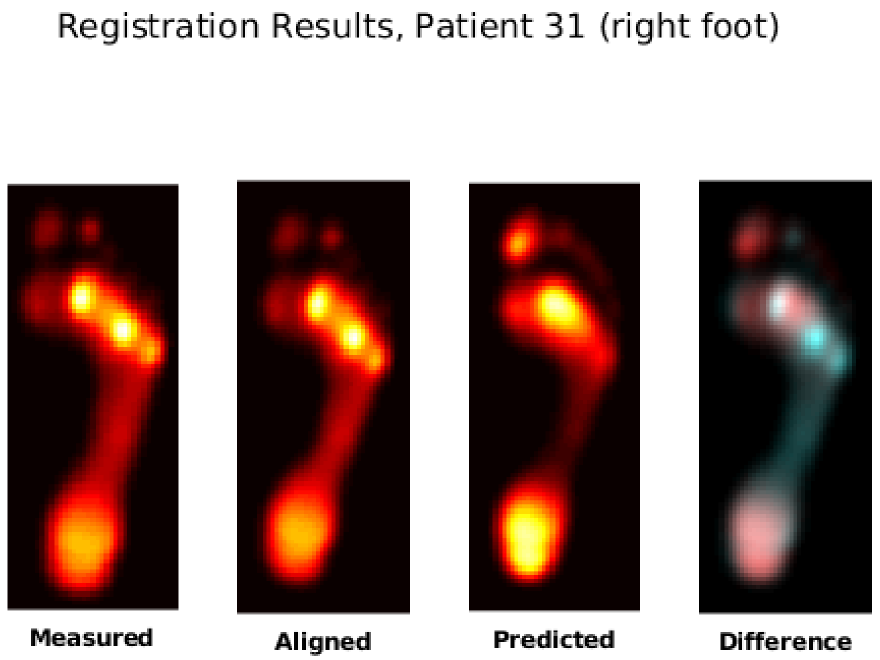
Registration Results, Patient 31 (right foot)
Measured
Aligned
Predicted
Difference

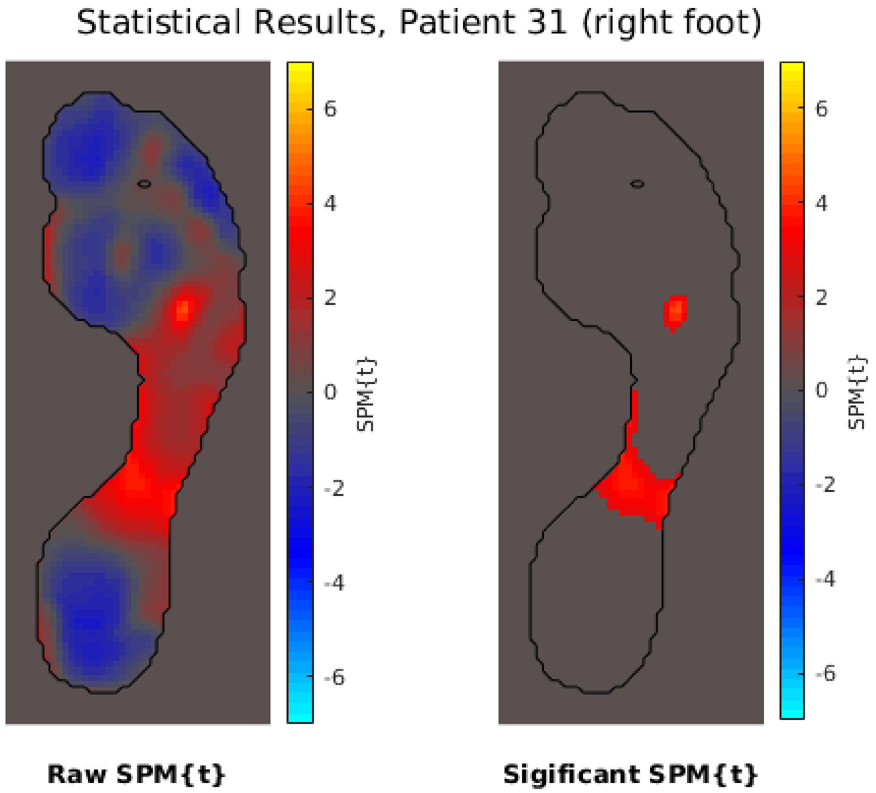
Statistical Results, Patient 31 (right foot)
6
4
2
0
-2
-4
-6
SPM{t}
Raw SPM{t}
Sigificant SPM{t}

Registration Results, Patient 32 (right foot)

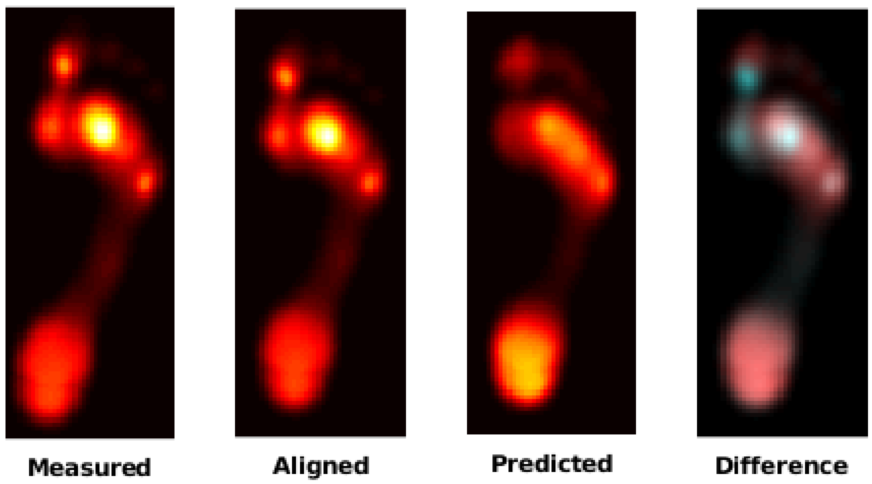

Statistical Results, Patient 32 (right foot)

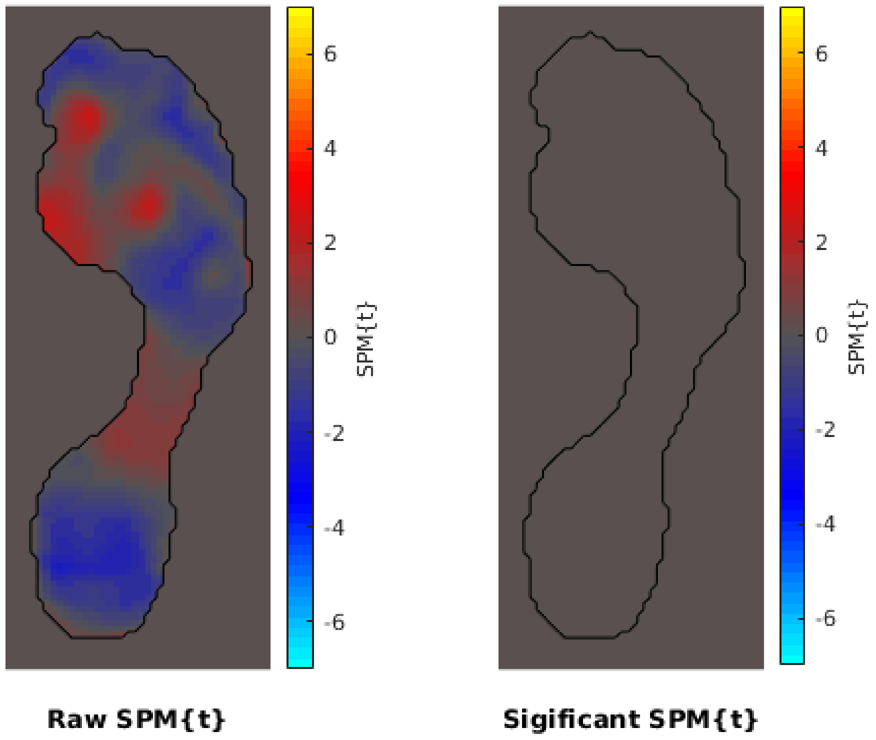

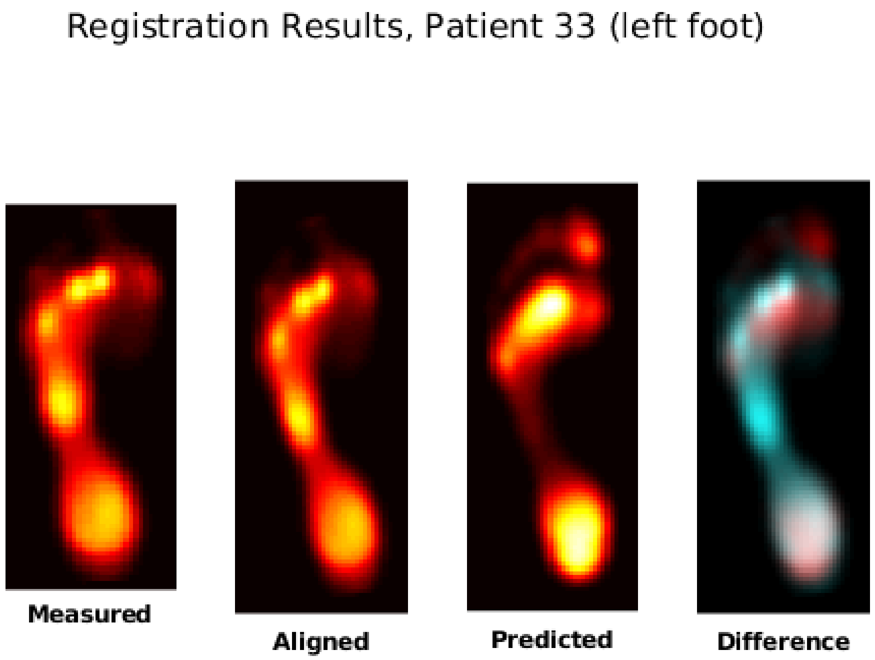
Registration Results, Patient 33 (left foot)
Measured
Aligned
Predicted
Difference

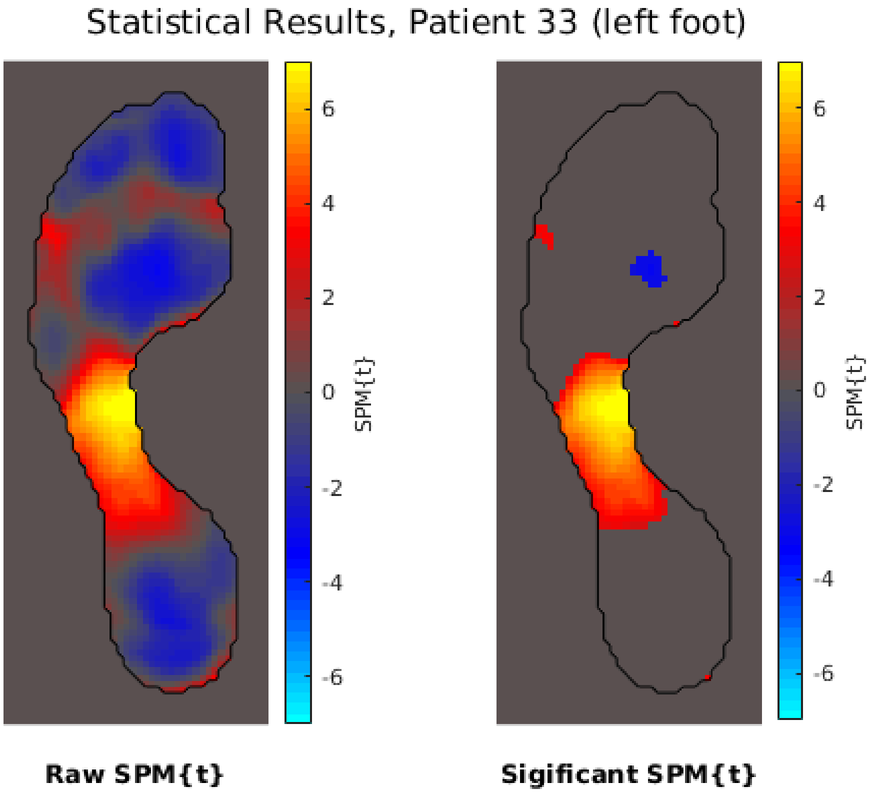
Statistical Results, Patient 33 (left foot)
6
4
2
0
-2
-4
-6
SPM{t}
Raw SPM{t}
Sigificant SPM{t}

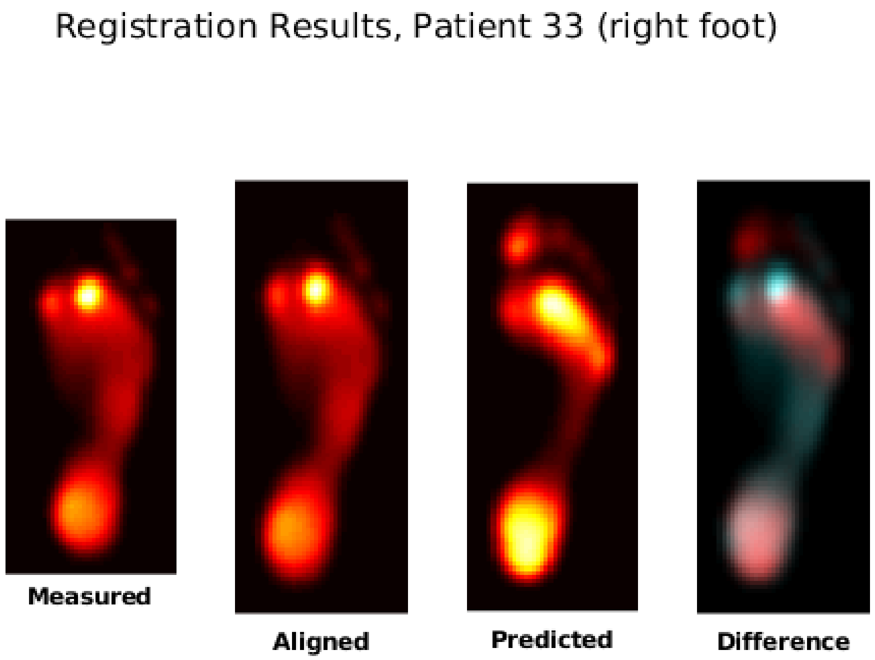
Registration Results, Patient 33 (right foot)
Measured
Aligned
Predicted
Difference

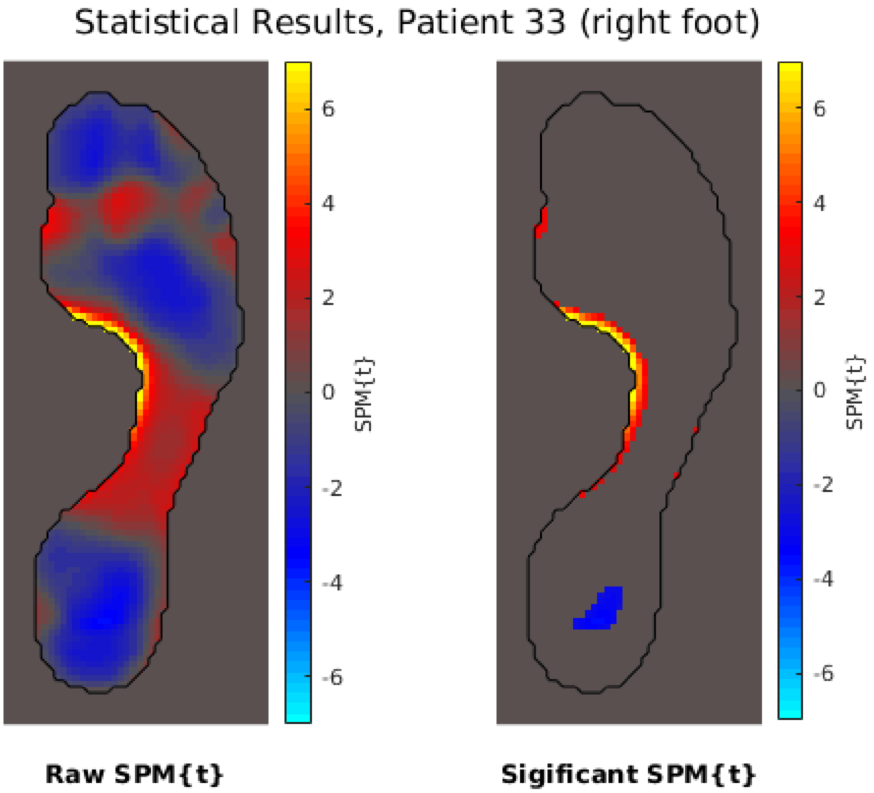
Statistical Results, Patient 33 (right foot)
6
4
2
0
-2
-4
-6
SPM{t}
Raw SPM{t}
Sigificant SPM{t}

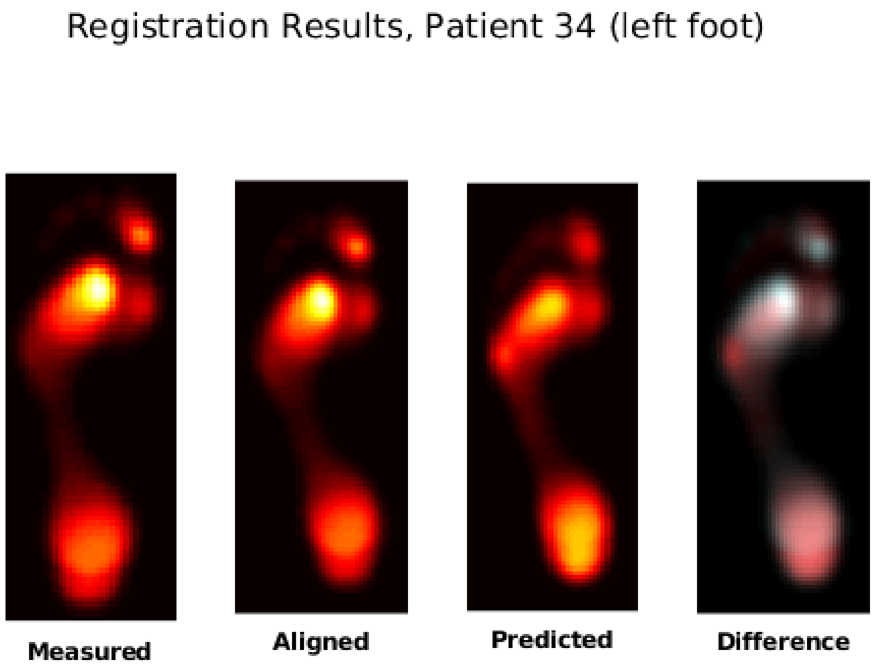
Registration Results, Patient 34 (left foot)
Measured
Aligned
Predicted
Difference

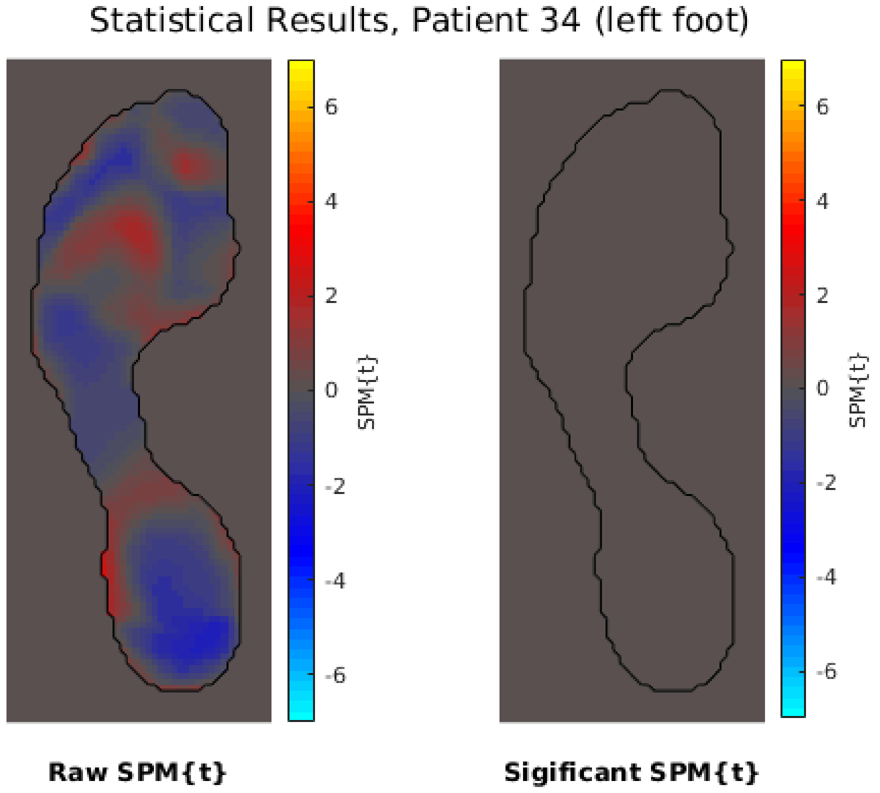
Statistical Results, Patient 34 (left foot)
6
4
2
0
-2
-4
-6
SPM{t}
Raw SPM{t}
Sigificant SPM{t}

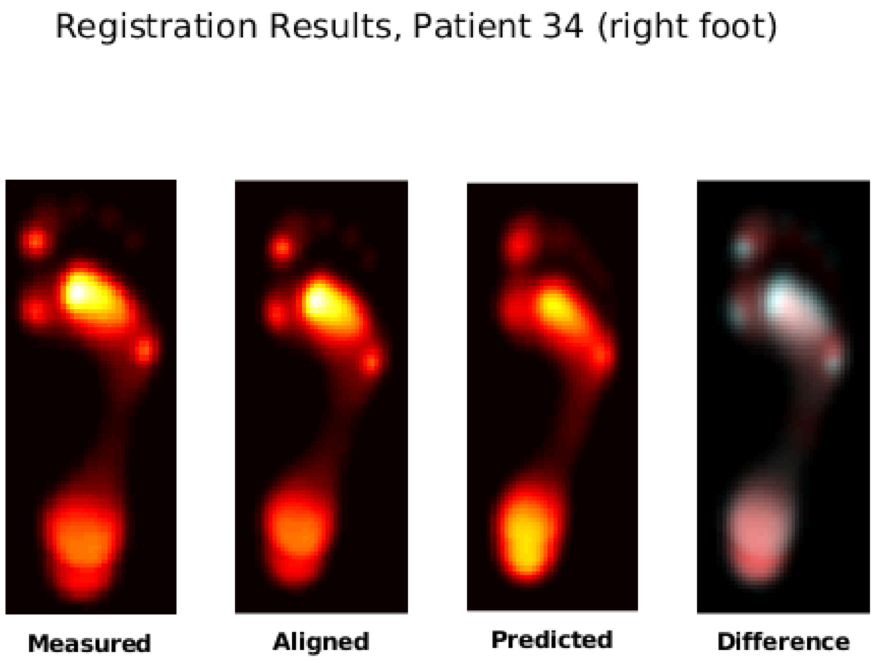
Registration Results, Patient 34 (right foot)
Measured
Aligned
Predicted
Difference

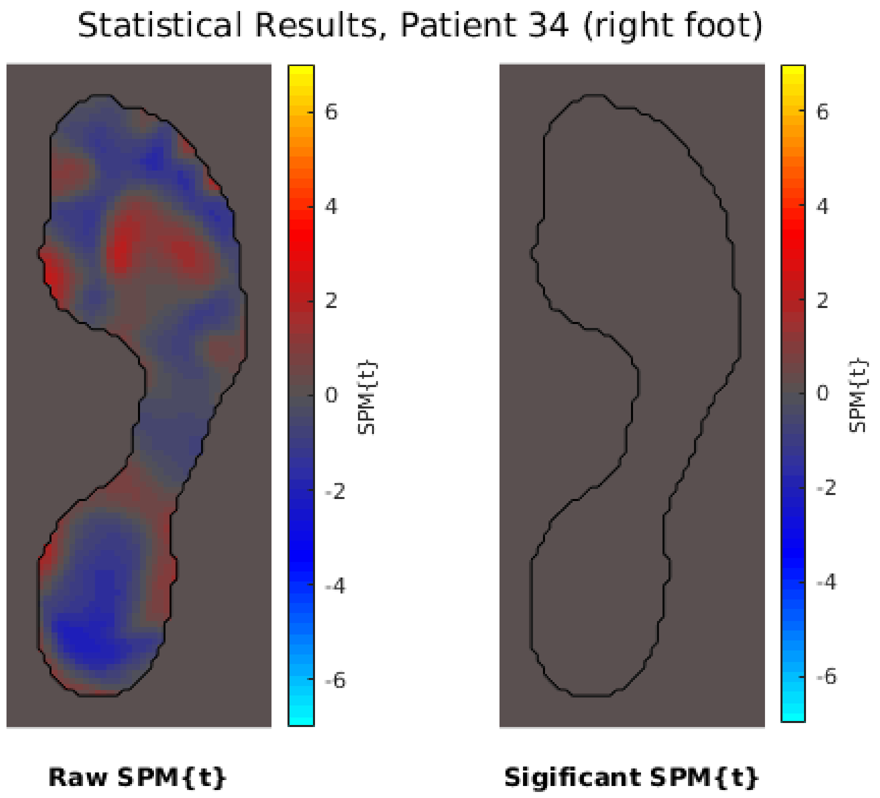
Statistical Results, Patient 34 (right foot)
6
4
2
0
-2
-4
-6
SPM{t}
Raw SPM{t}
Sigificant SPM{t}

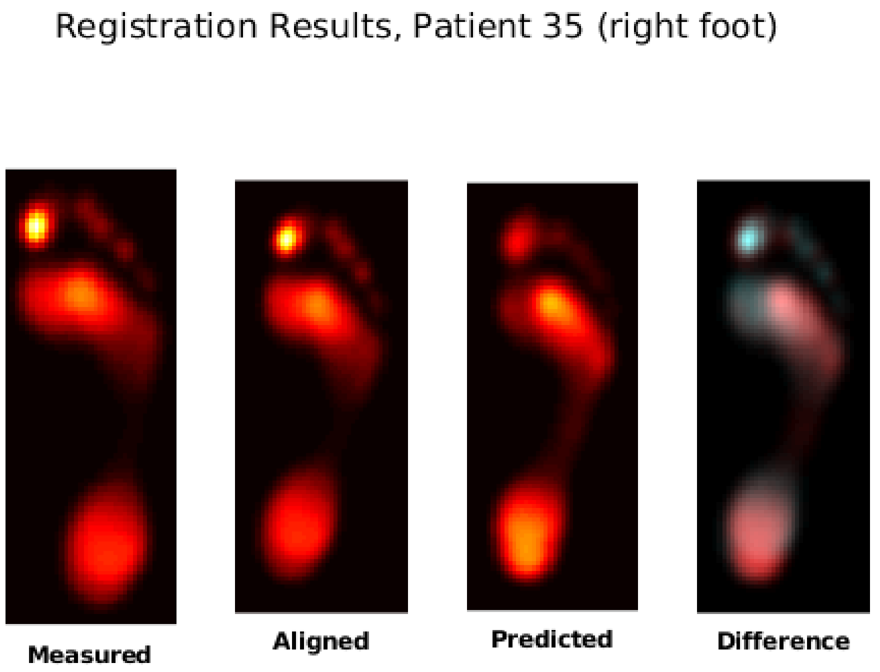
Registration Results, Patient 35 (right foot)
Measured
Aligned
Predicted
Difference

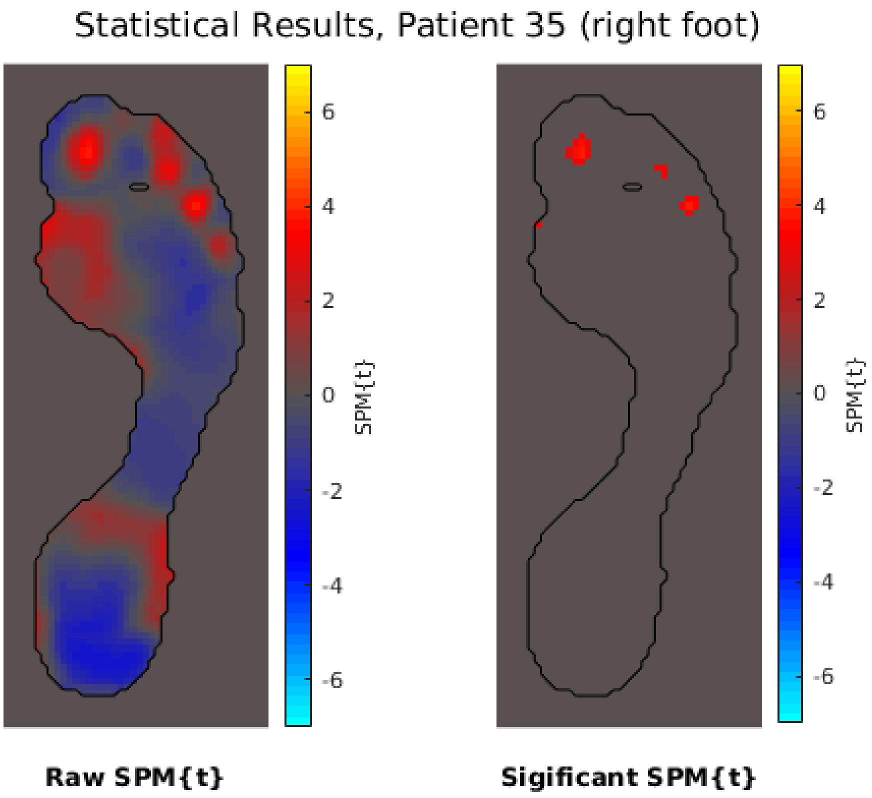
Statistical Results, Patient 35 (right foot)
6
4
2
0
-2
-4
-6
SPM{t}
Raw SPM{t}
Sigificant SPM{t}

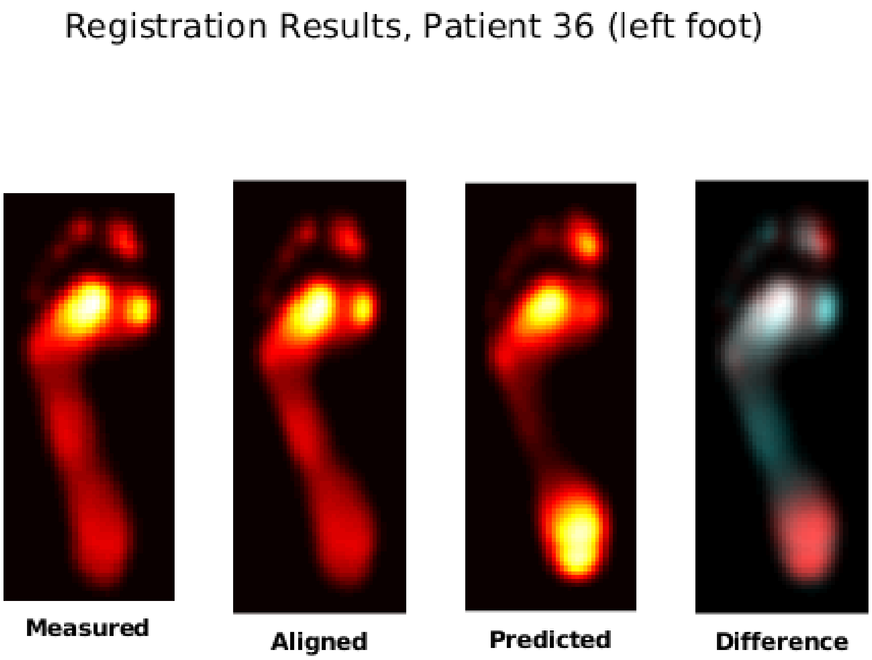
Registration Results, Patient 36 (left foot)
Measured
Aligned
Predicted
Difference

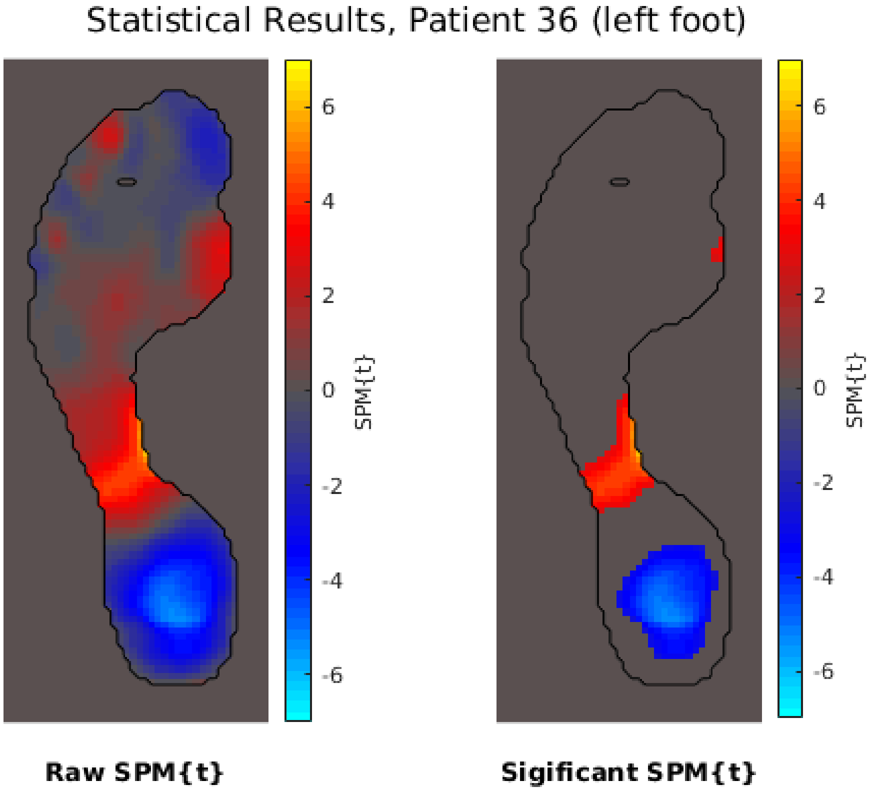
Statistical Results, Patient 36 (left foot)
6
4
2
0
-2
-4
-6
SPM{t}
Raw SPM{t}
Sigificant SPM{t}

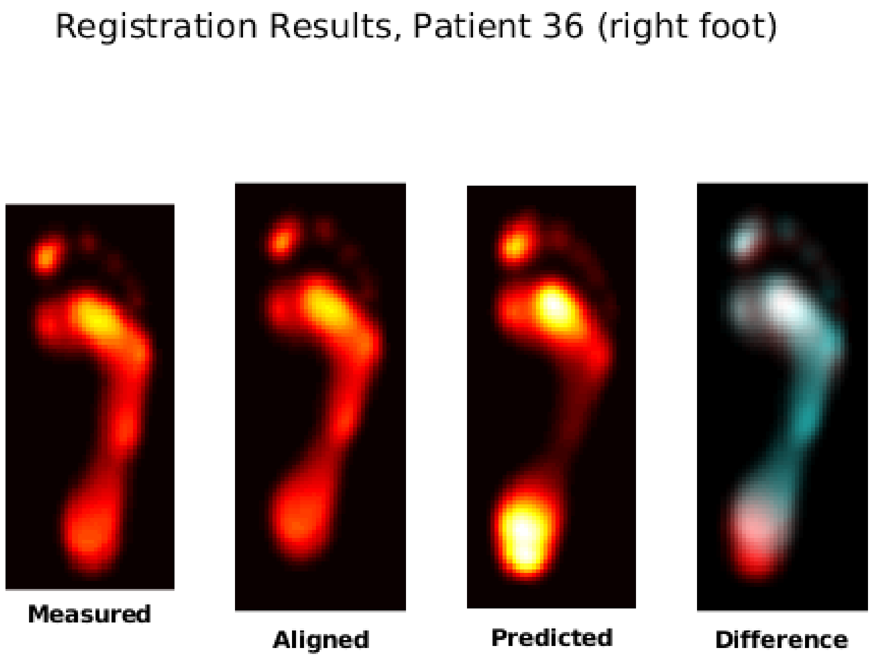
Registration Results, Patient 36 (right foot)
Measured
Aligned
Predicted
Difference

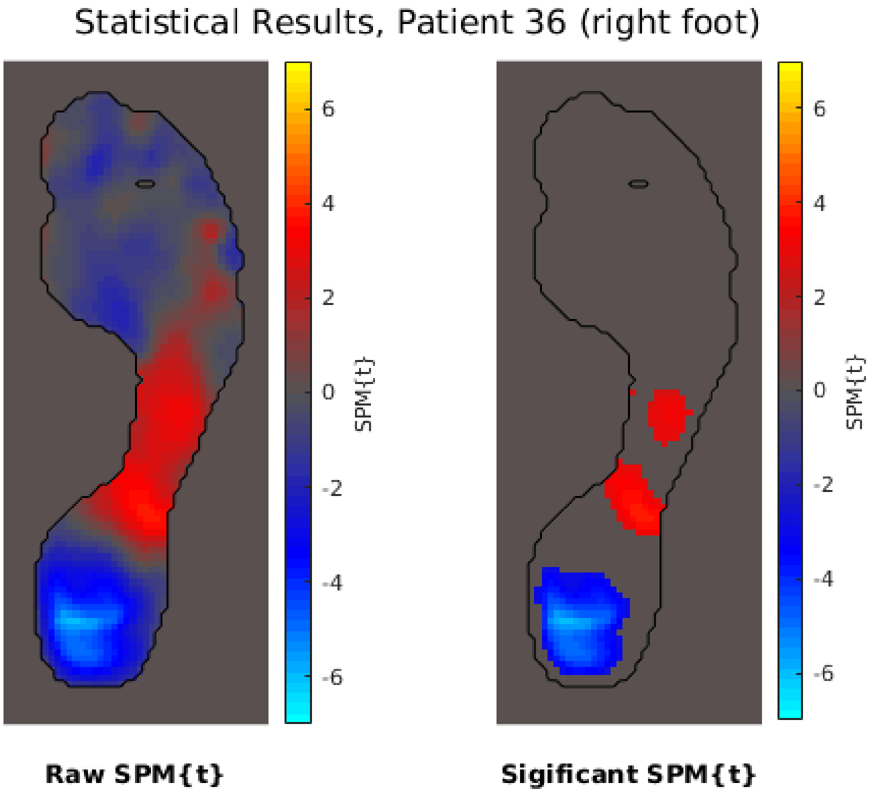
Statistical Results, Patient 36 (right foot)
6
4
2
0
-2
-4
-6
SPM{t}
Raw SPM{t}
Sigificant SPM{t}

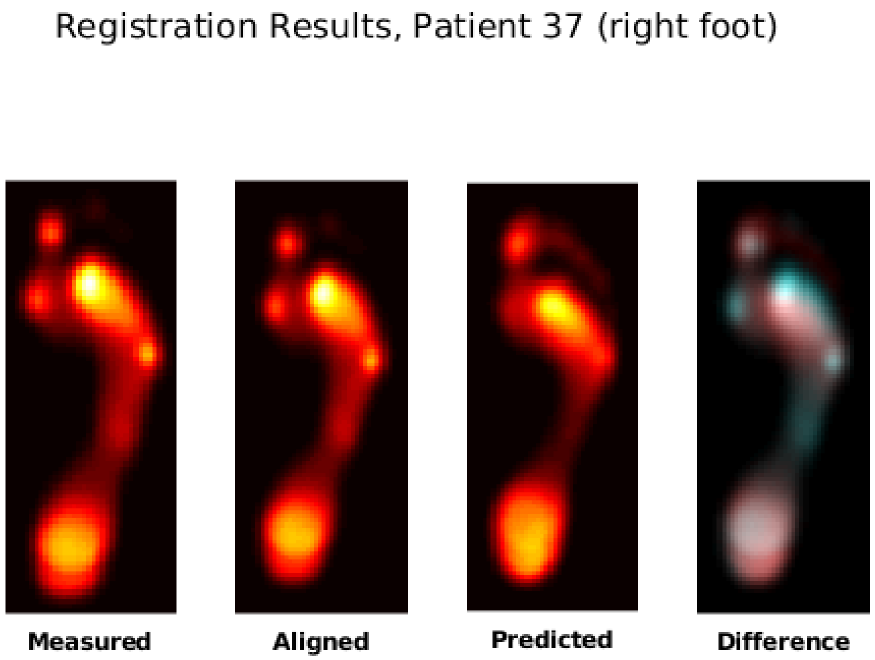
Registration Results, Patient 37 (right foot)
Measured
Aligned
Predicted
Difference

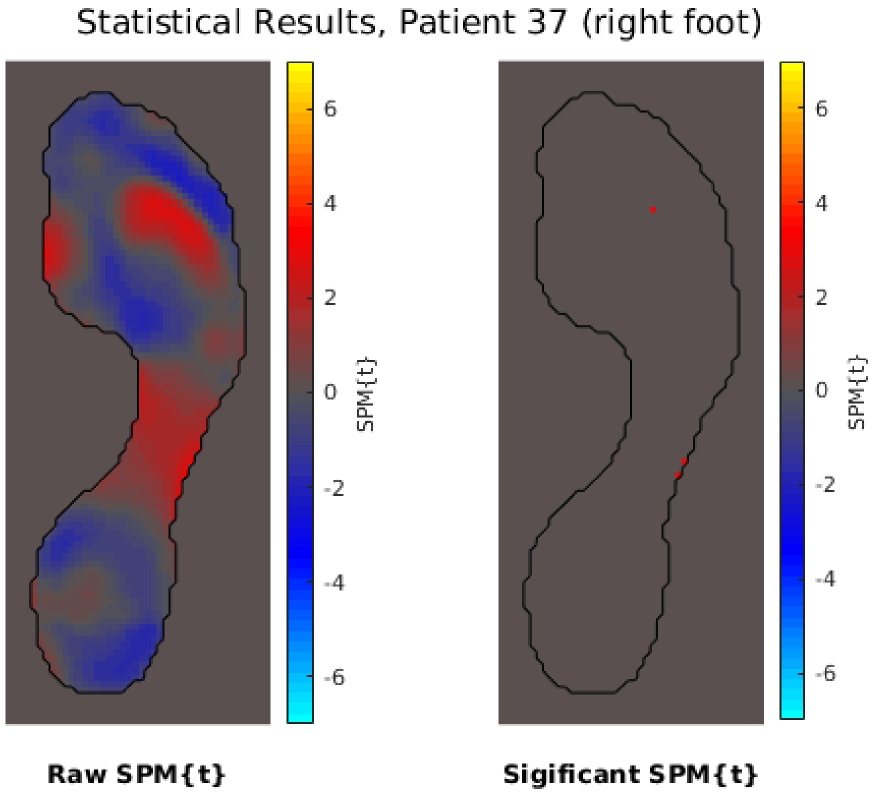
Statistical Results, Patient 37 (right foot)
6
4
2
0
-2
-4
-6
SPM{t}
Raw SPM{t}
Sigificant SPM{t}

Registration Results, Patient 38 (left foot)

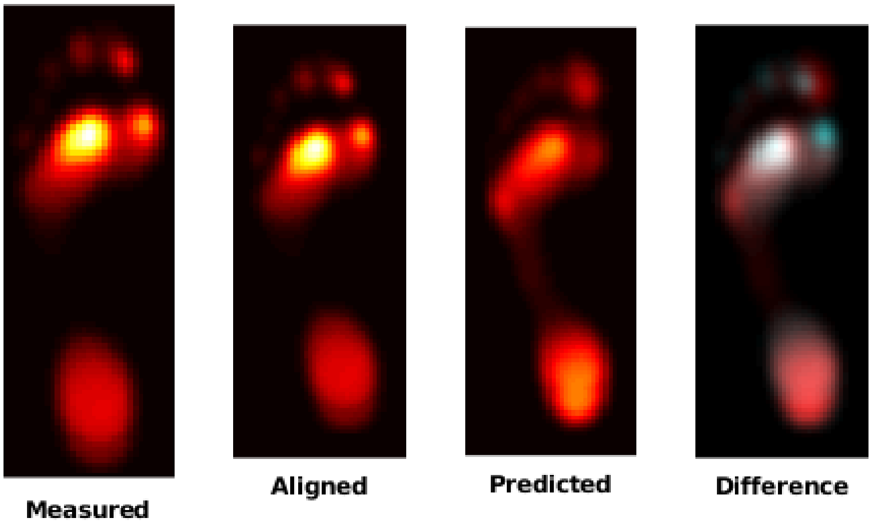

Statistical Results, Patient 38 (left foot)

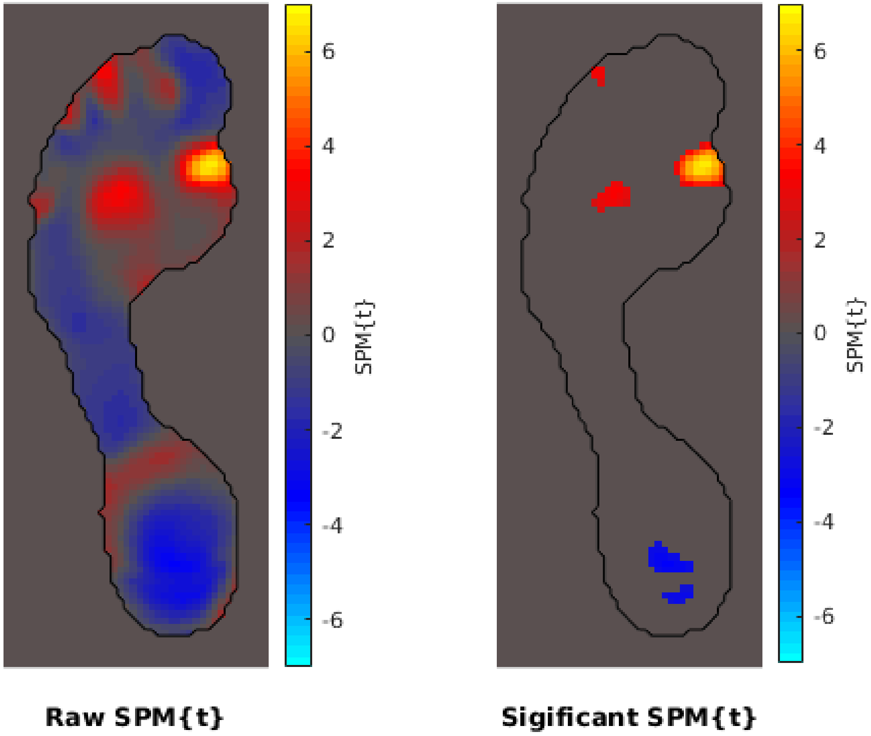

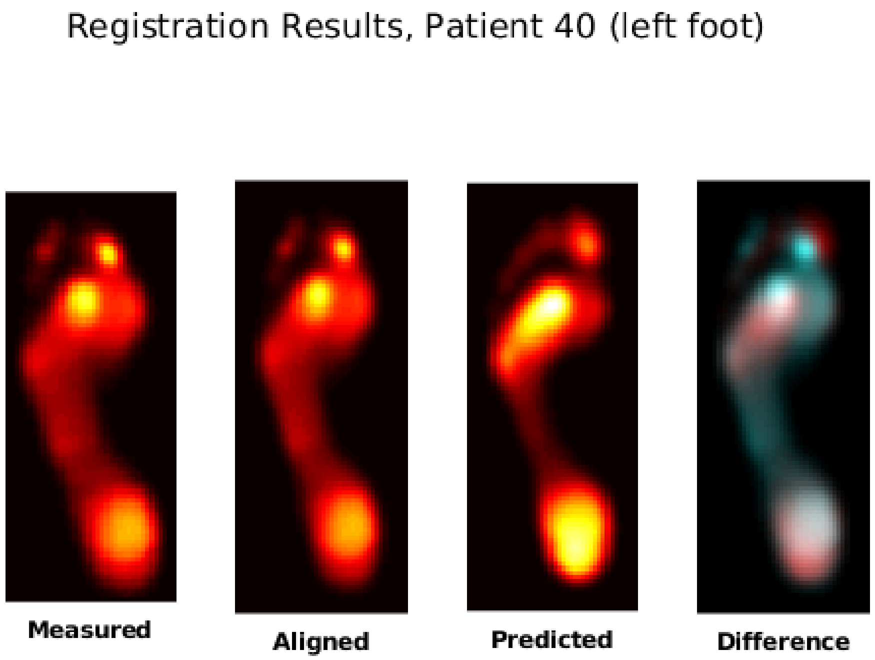
Registration Results, Patient 40 (left foot)
Measured
Aligned
Predicted
Difference

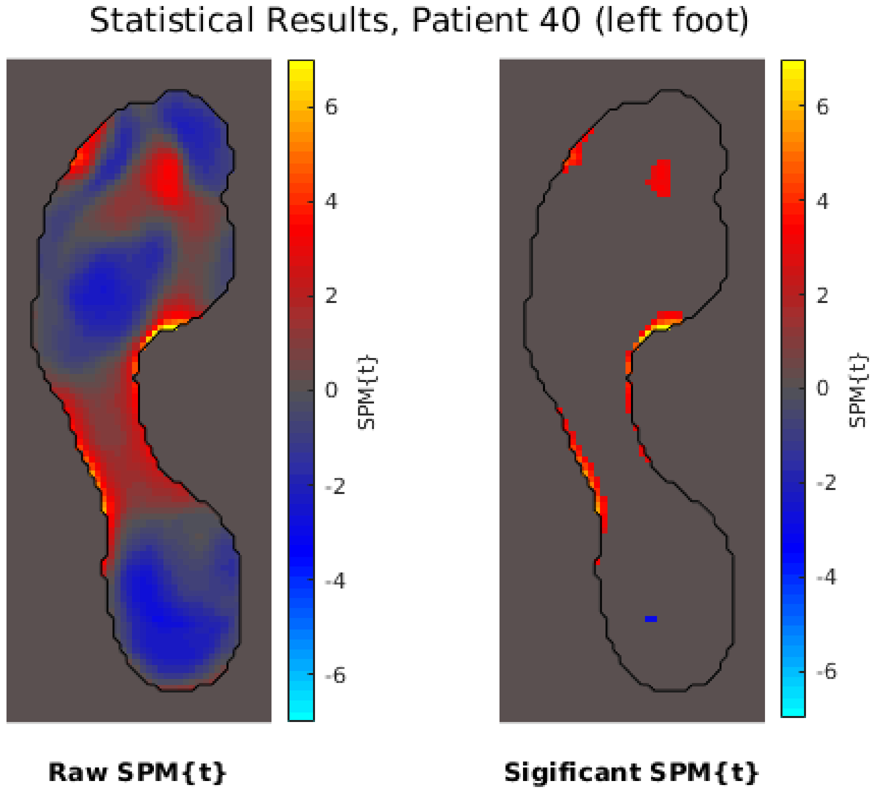
Statistical Results, Patient 40 (left foot)
6
4
2
0
-2
-4
-6
SPM{t}
6
4
2
0
-2
-4
-6
SPM{t}
Raw SPM{t}
Sigificant SPM{t}

Registration Results, Patient 42 (left foot)

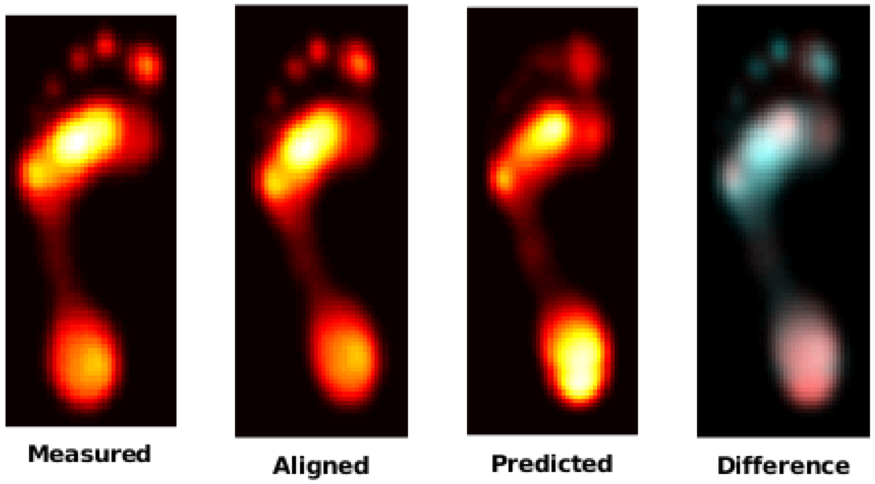

Statistical Results, Patient 42 (left foot)

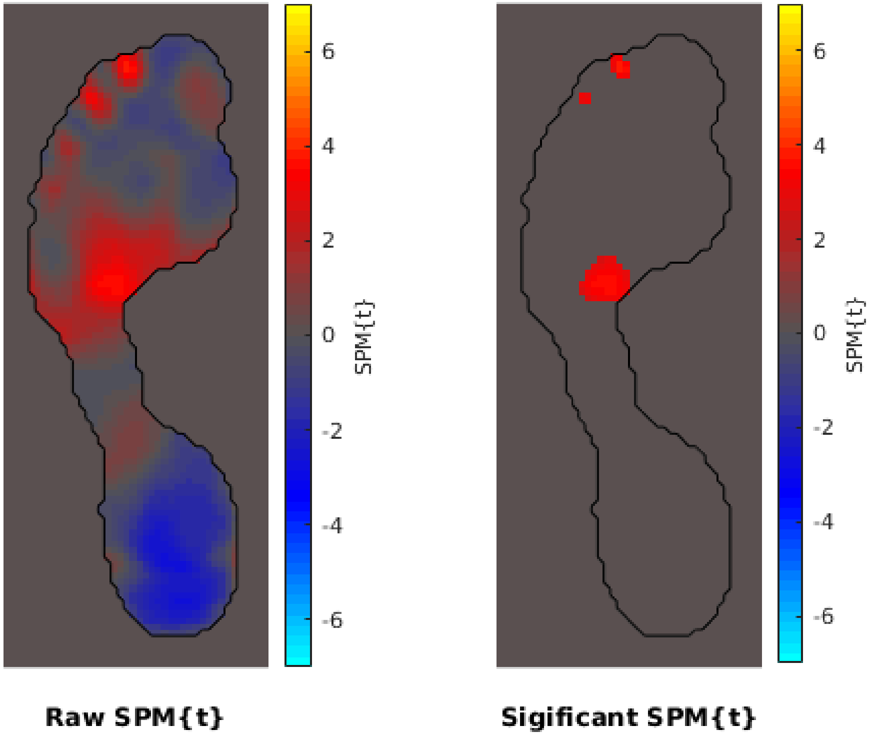

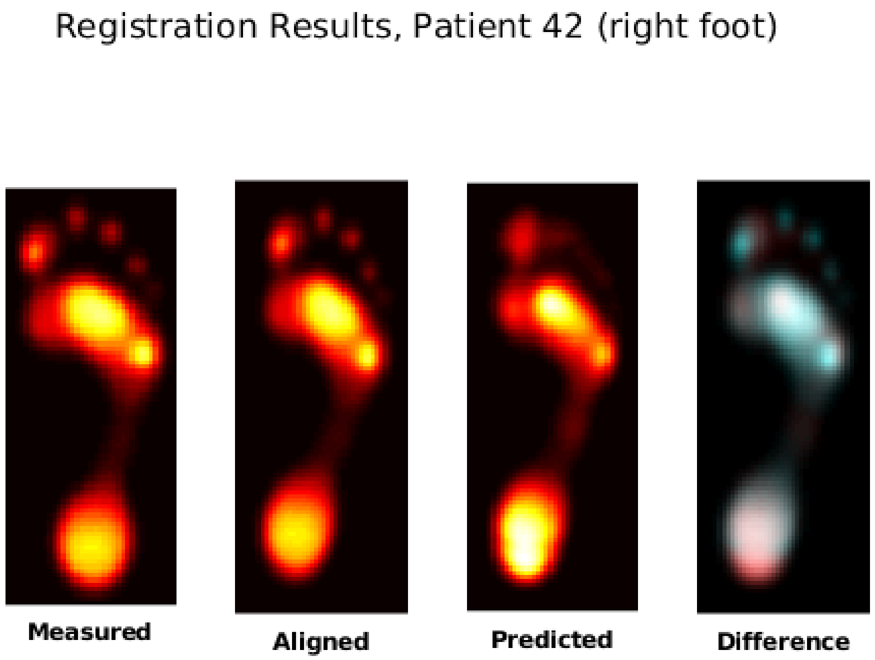
Registration Results, Patient 42 (right foot)
Measured
Aligned
Predicted
Difference

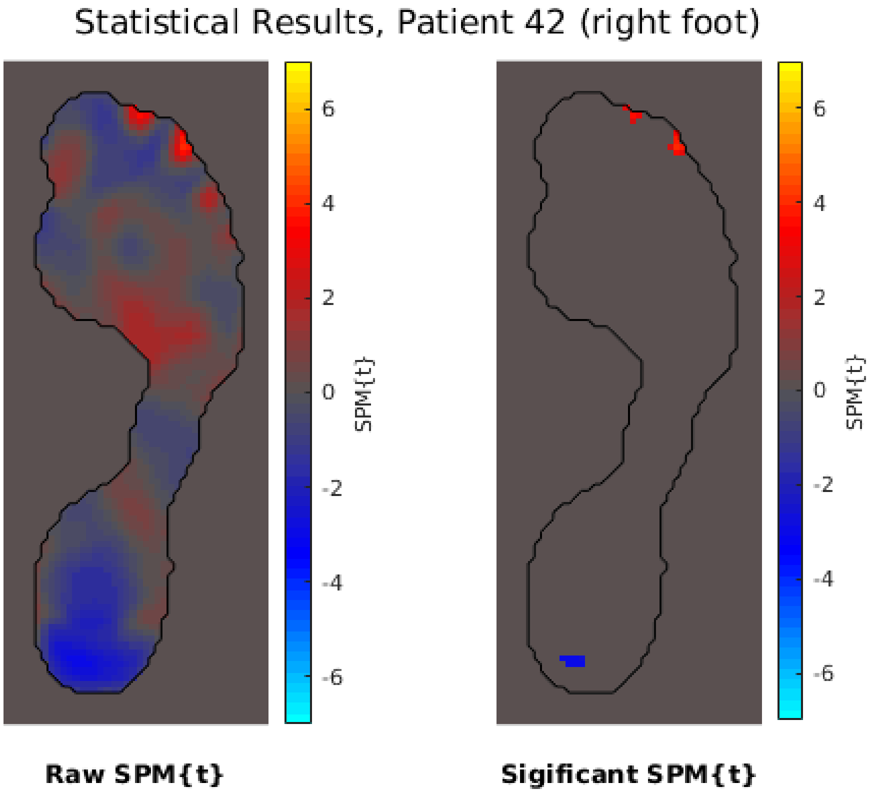
Statistical Results, Patient 42 (right foot)
SPM{t}
6
4
2
0
-2
-4
-6
Raw SPM{t}
Sigificant SPM{t}

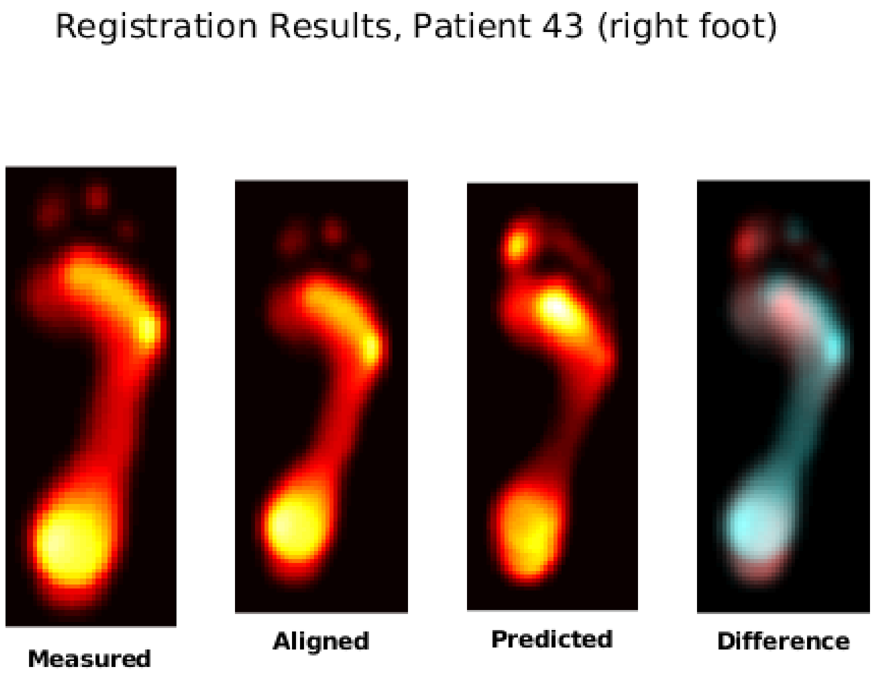
Registration Results, Patient 43 (right foot)
Measured
Aligned
Predicted
Difference

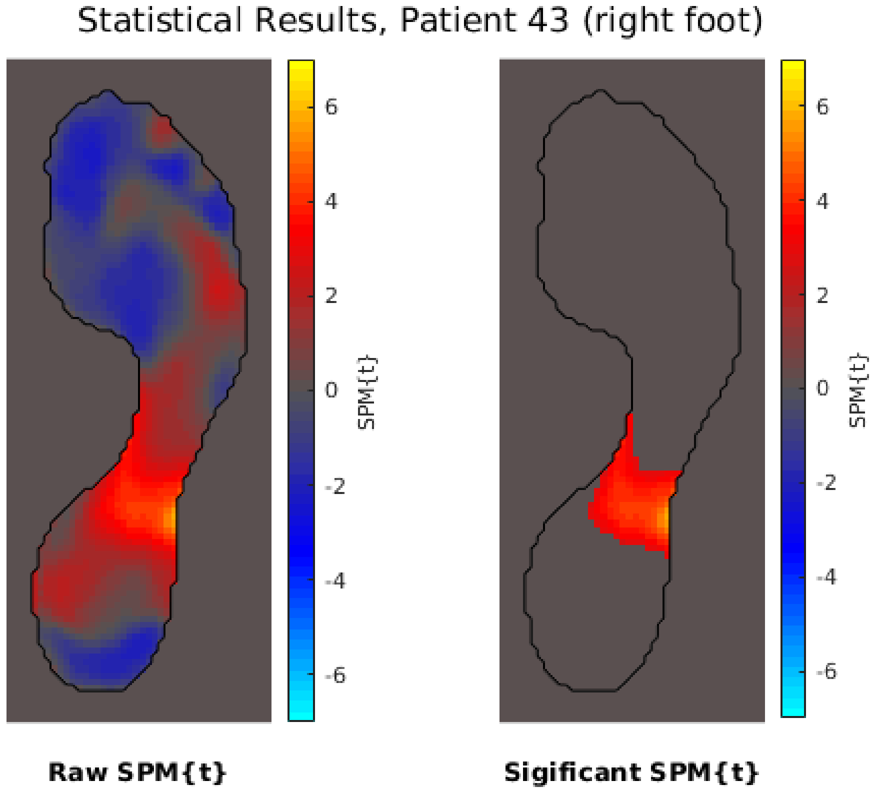
Statistical Results, Patient 43 (right foot)
6
4
2
0
-2
-4
-6
SPM{t}
Raw SPM{t}
Sigificant SPM{t}

Registration Results, Patient 44 (left foot)

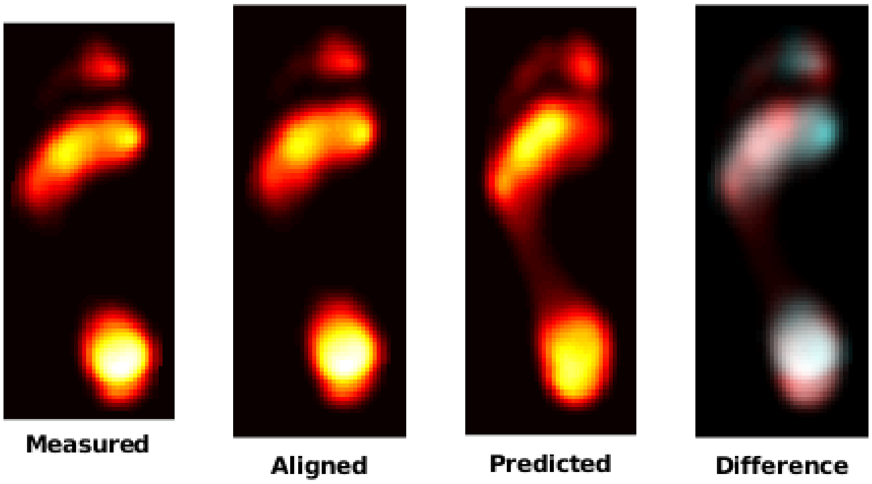

Statistical Results, Patient 44 (left foot)

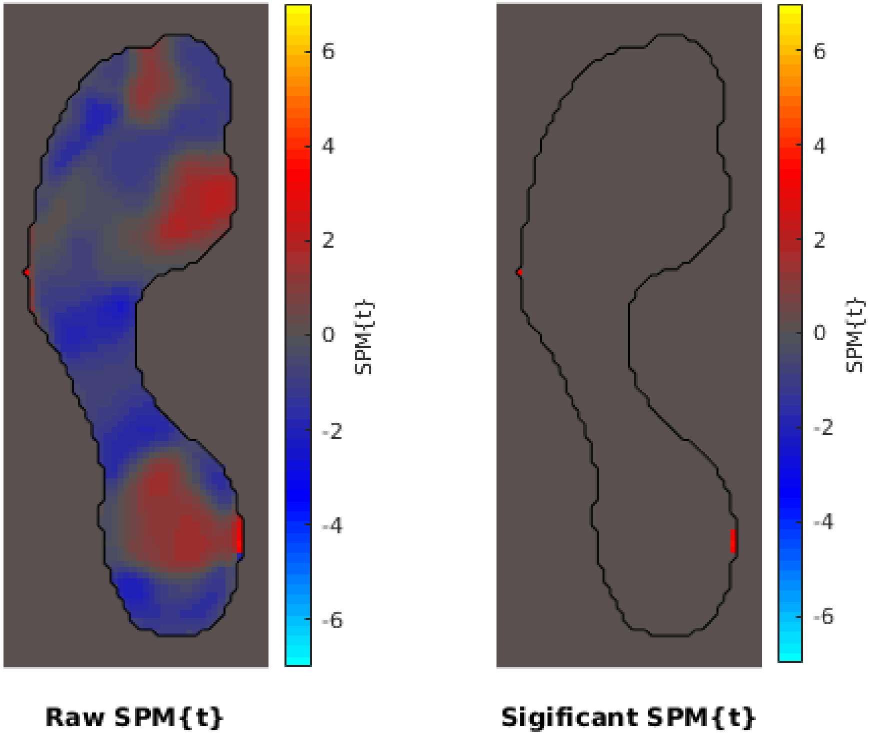

Registration Results, Patient 44 (right foot)

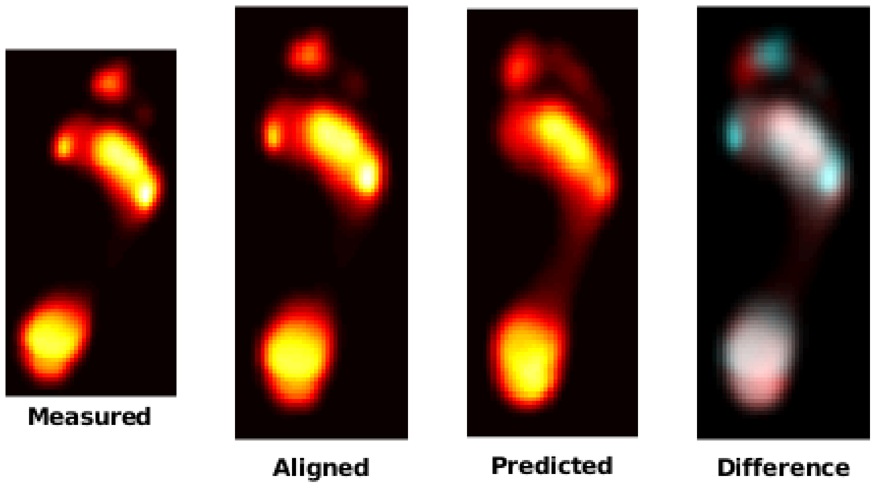

Statistical Results, Patient 44 (right foot)

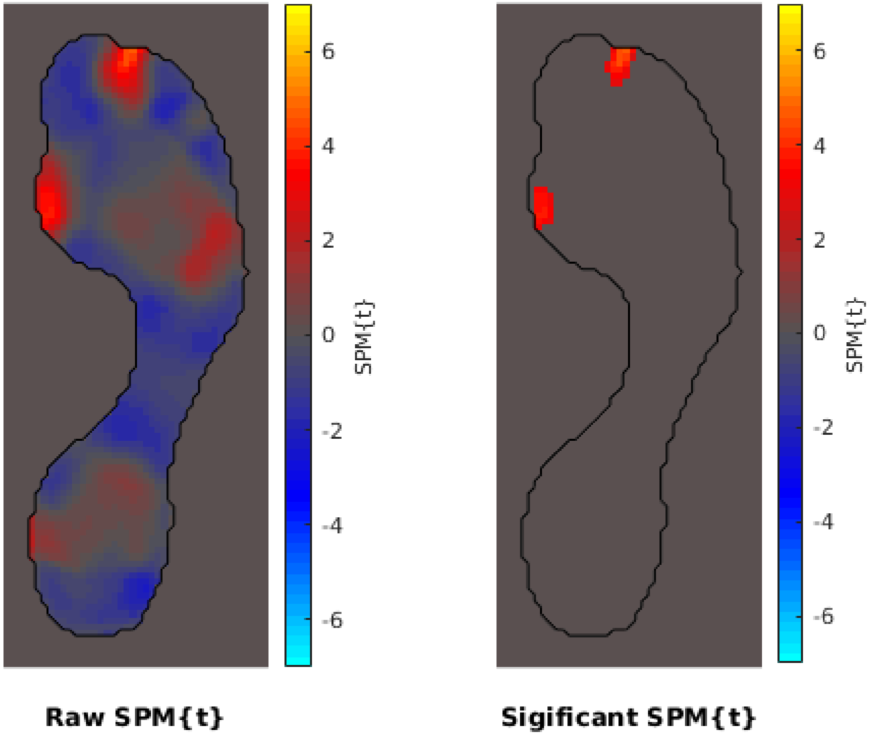

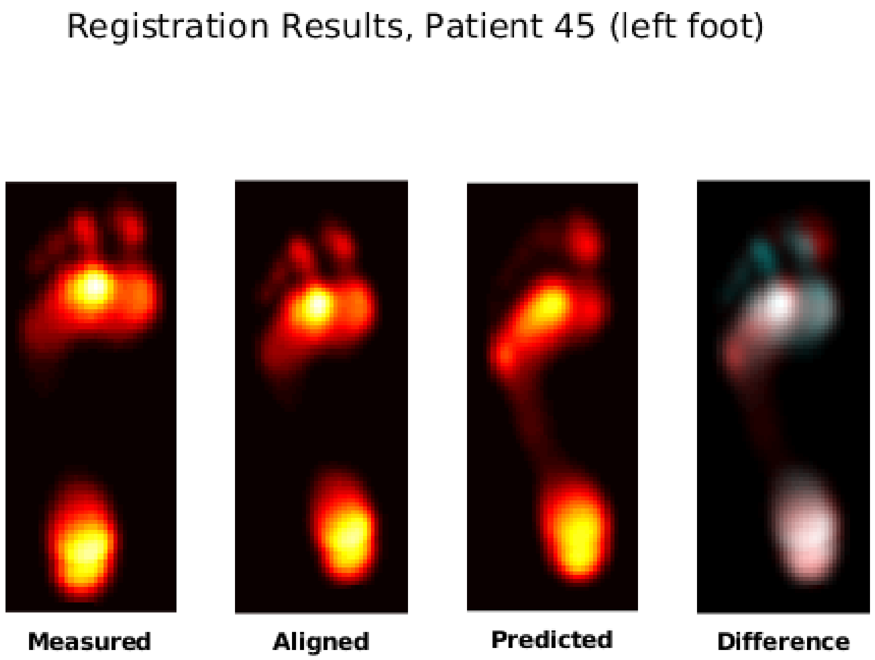
Registration Results, Patient 45 (left foot)
Measured
Aligned
Predicted
Difference

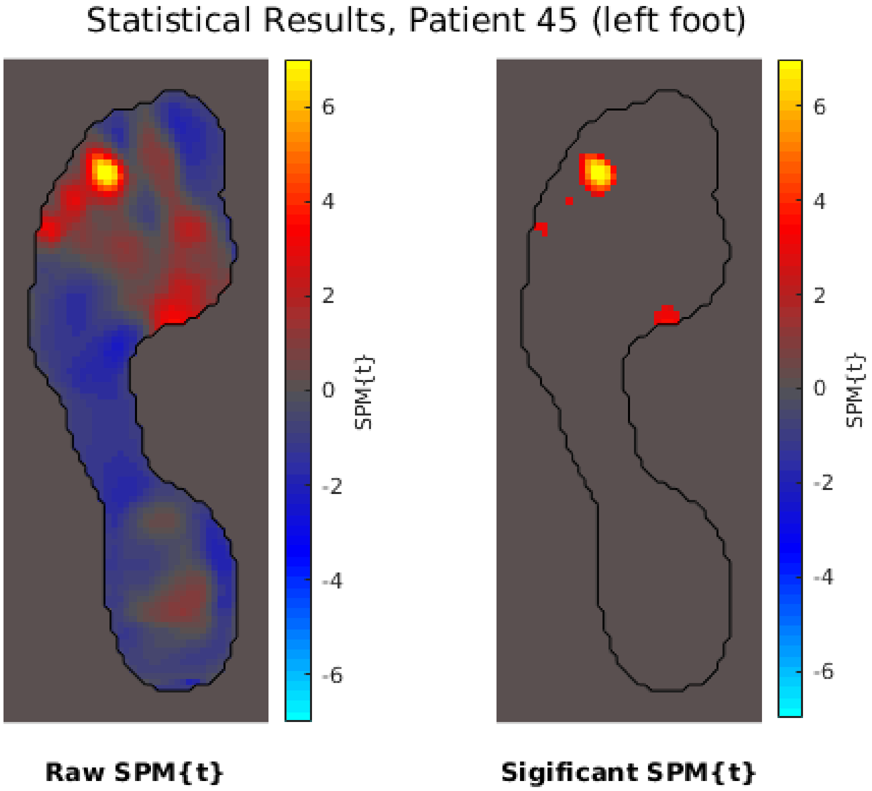
Statistical Results, Patient 45 (left foot)
6
4
2
0
-2
-4
-6
SPM{t}
Raw SPM{t}
Sigificant SPM{t}

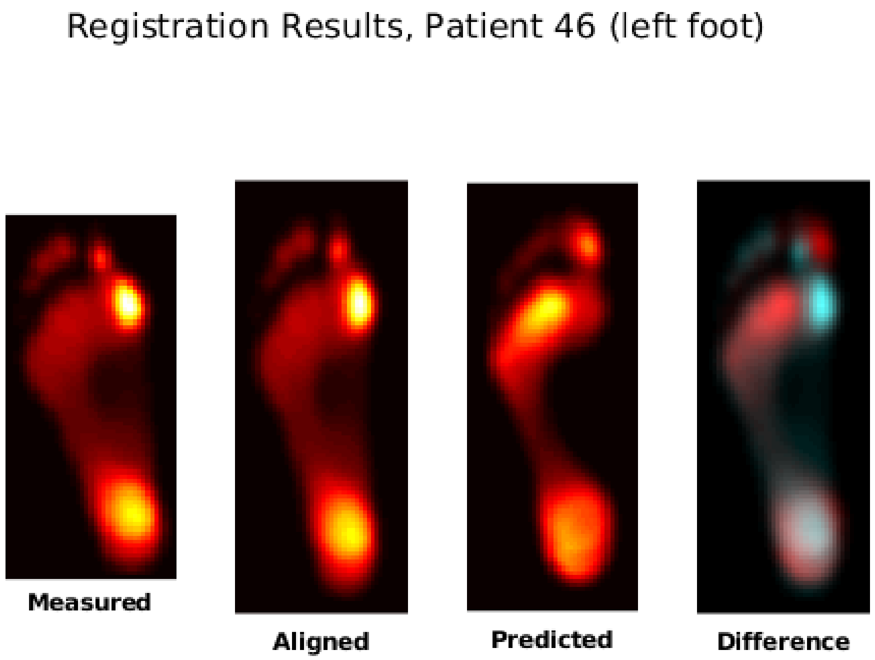
Registration Results, Patient 46 (left foot)
Measured
Aligned
Predicted
Difference

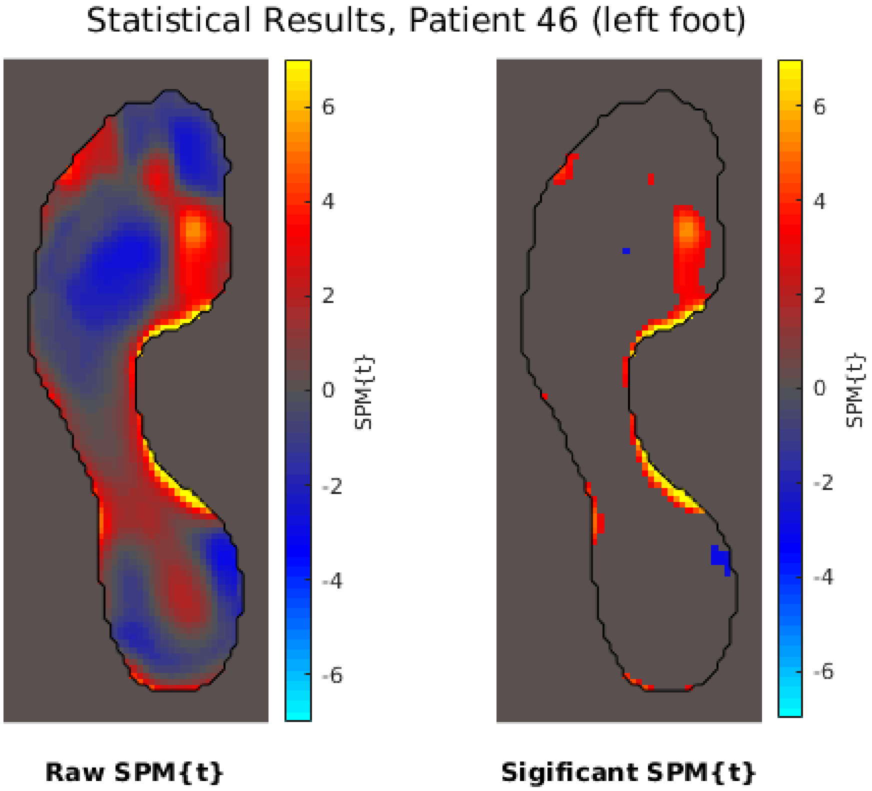
Statistical Results, Patient 46 (left foot)
6
4
2
0
-2
-4
-6
SPM{t}
Raw SPM{t}
Sigificant SPM{t}

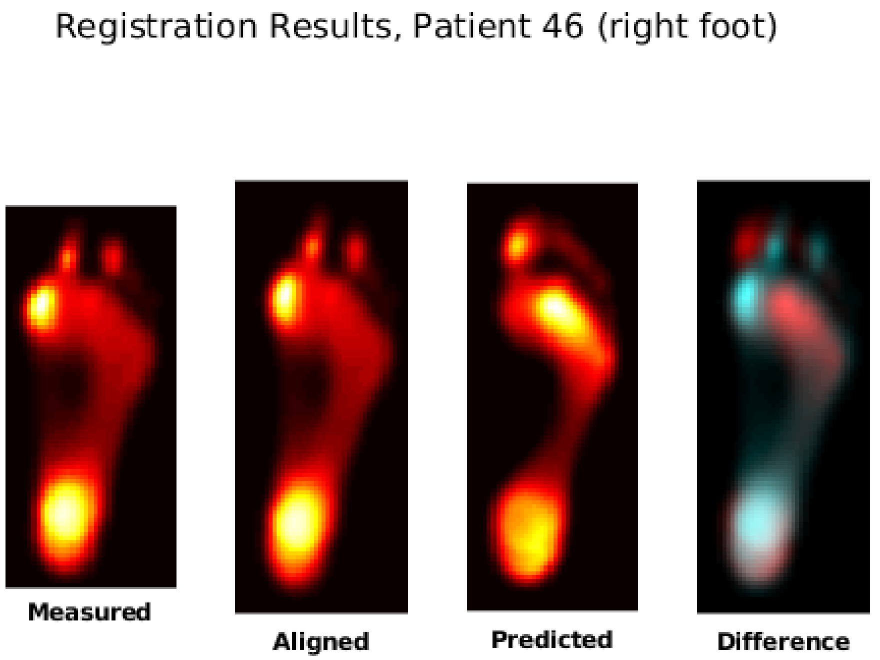
Registration Results, Patient 46 (right foot)
Measured
Aligned
Predicted
Difference

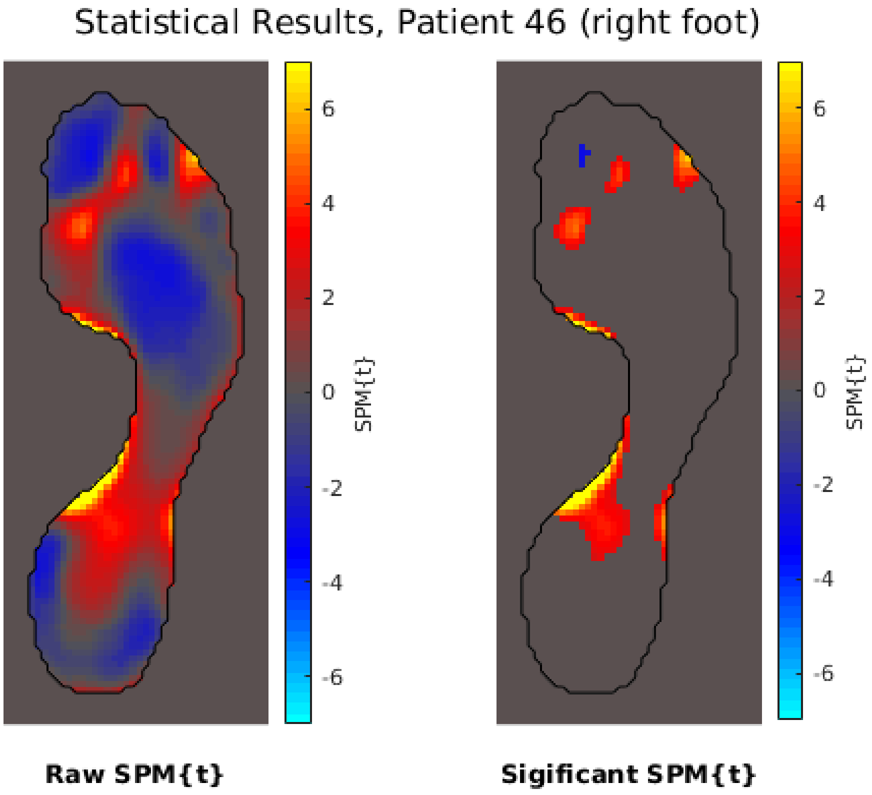
Statistical Results, Patient 46 (right foot)
6
4
2
0
-2
-4
-6
SPM{t}
Raw SPM{t}
Sigificant SPM{t}

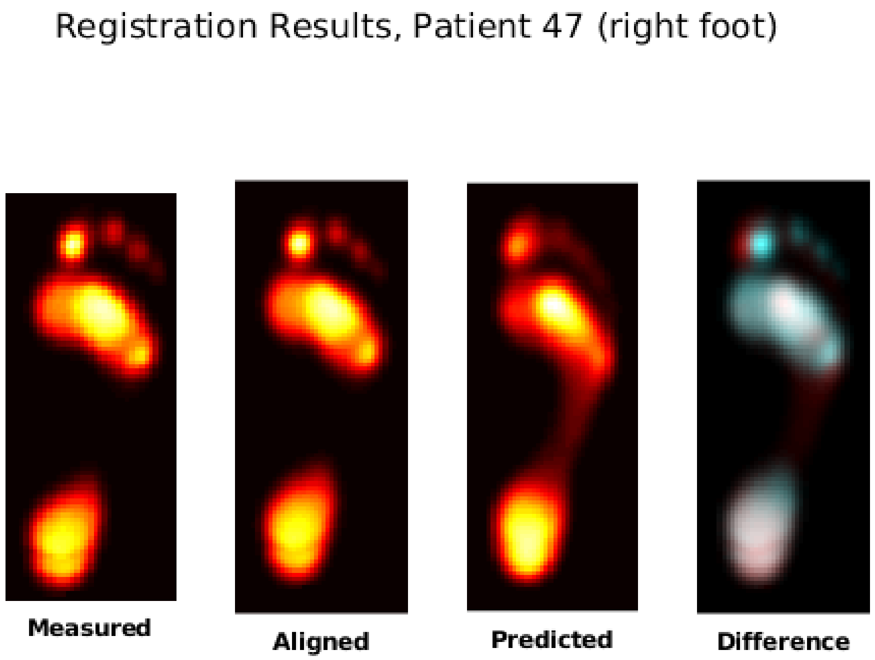
Registration Results, Patient 47 (right foot)
Measured
Aligned
Predicted
Difference

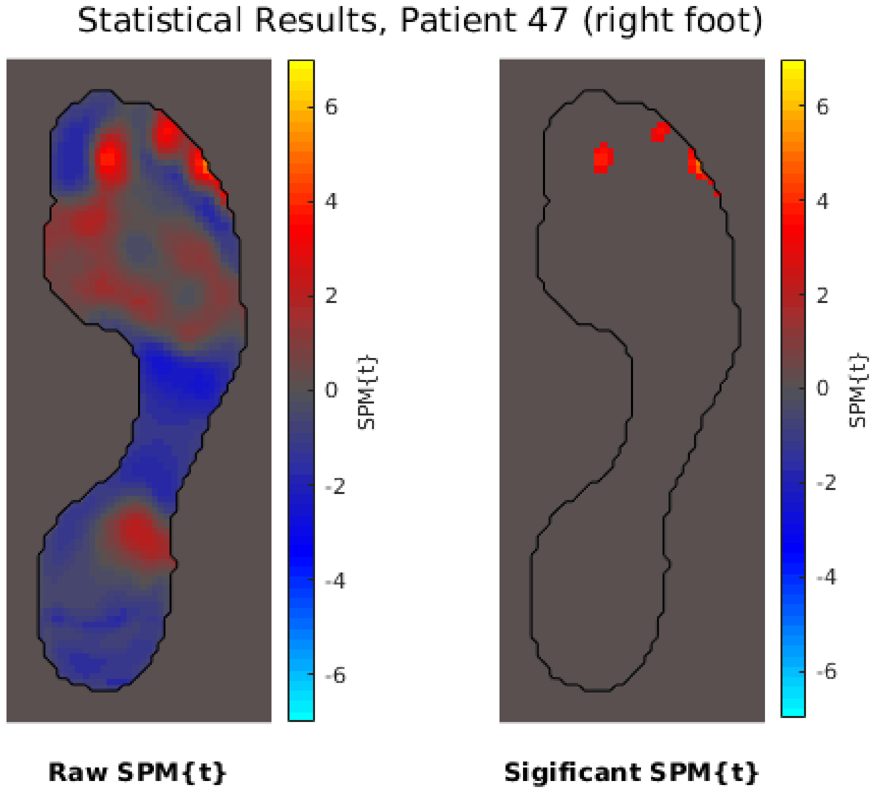
Statistical Results, Patient 47 (right foot)
6
4
2
0
-2
-4
-6
SPM{t}
Raw SPM{t}
Sigificant SPM{t}

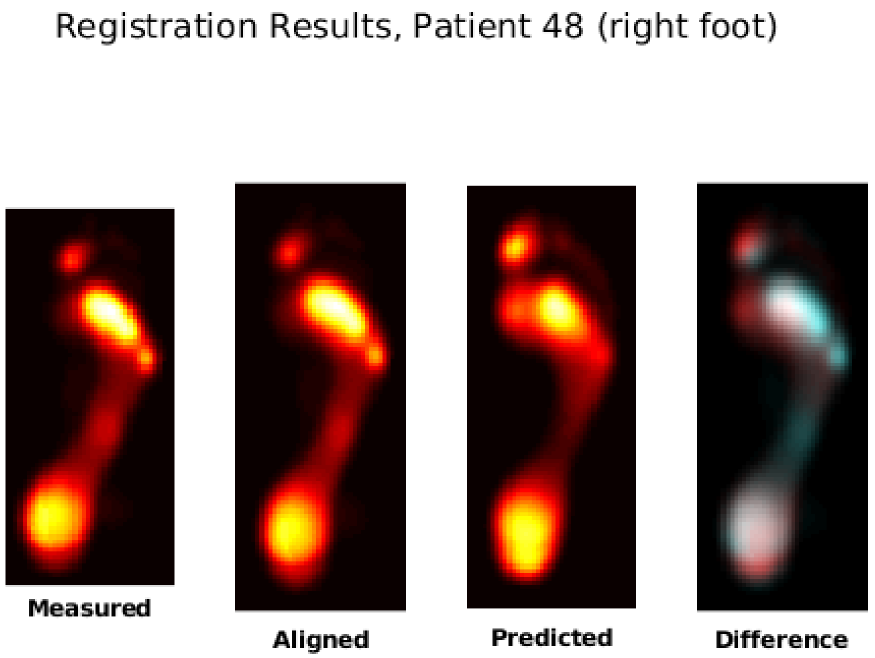
Registration Results, Patient 48 (right foot)
Measured
Aligned
Predicted
Difference

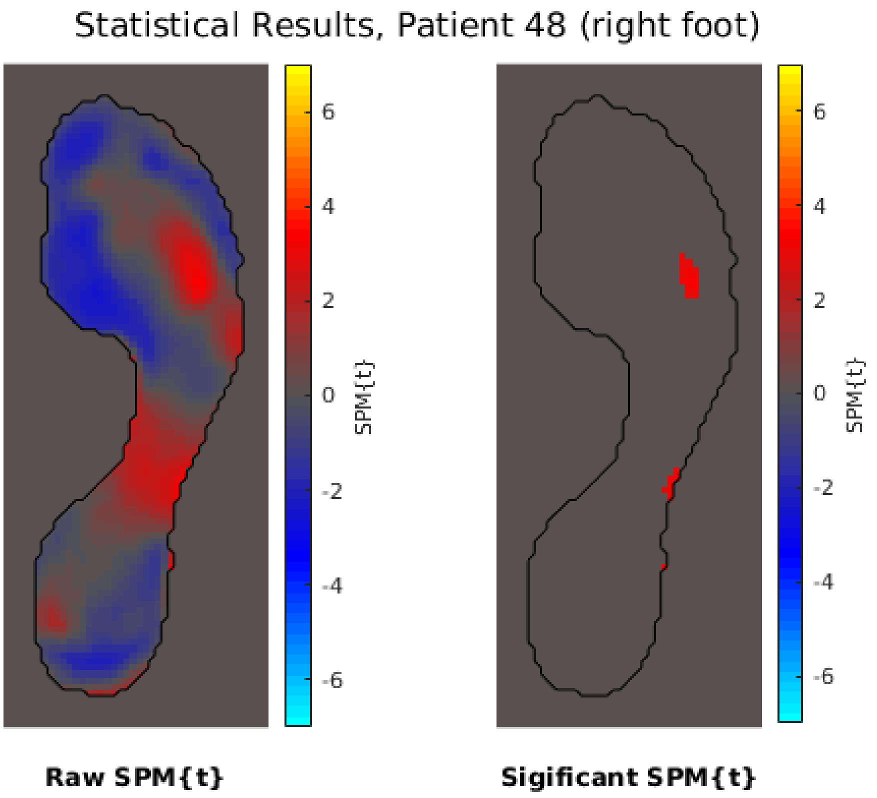
Statistical Results, Patient 48 (right foot)
6
4
2
0
-2
-4
-6
SPM{t}
Raw SPM{t}
Sigificant SPM{t}

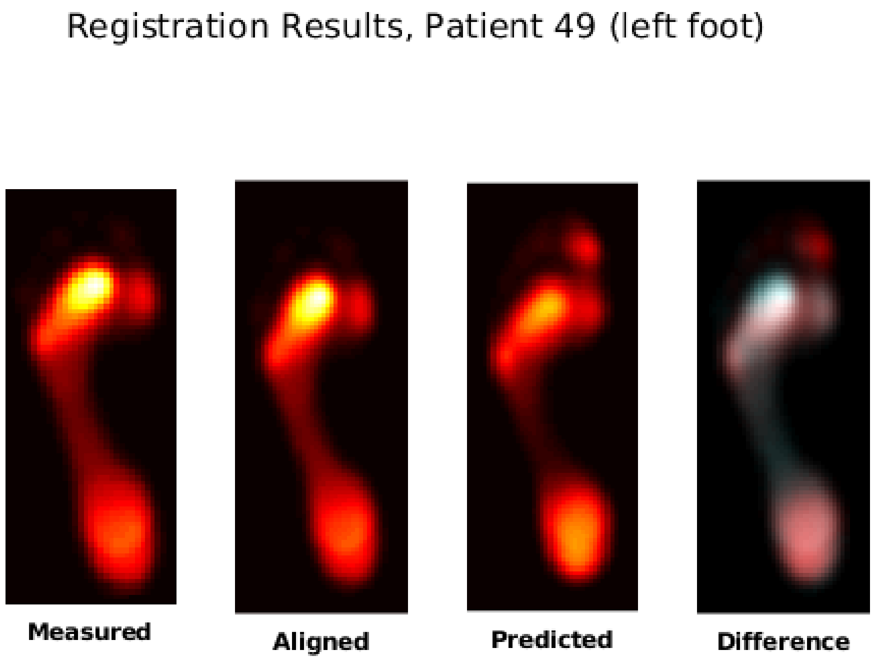
Registration Results, Patient 49 (left foot)
Measured
Aligned
Predicted
Difference

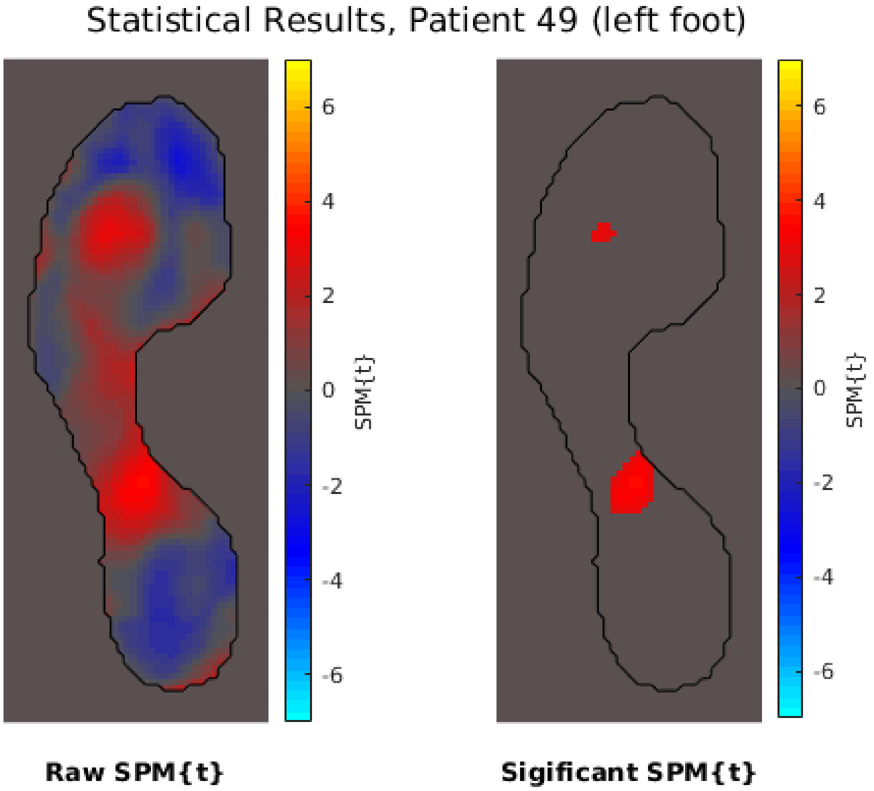
Statistical Results, Patient 49 (left foot)
6
4
2
0
-2
-4
-6
SPM{t}
Raw SPM{t}
Sigificant SPM{t}

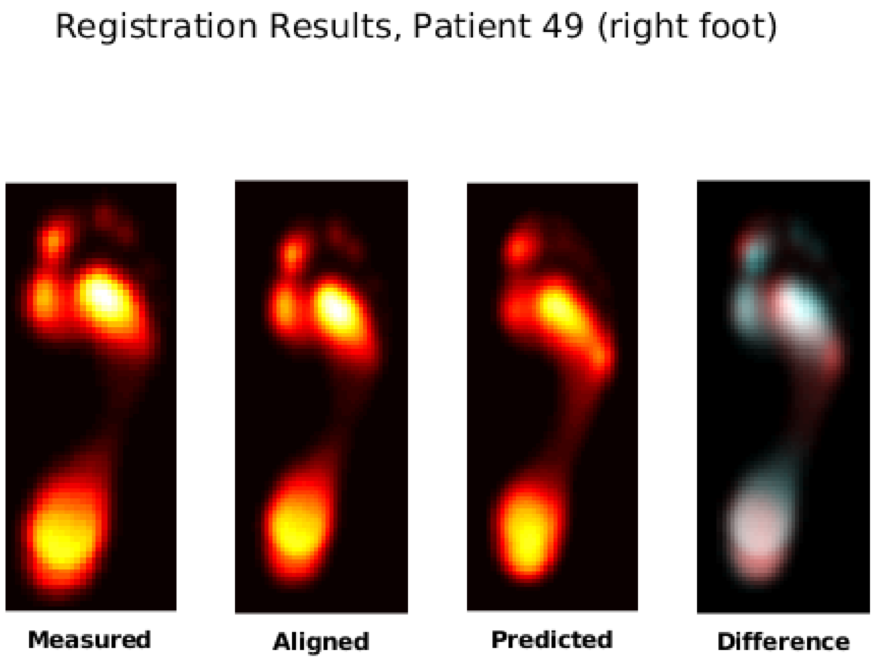
Registration Results, Patient 49 (right foot)
Measured
Aligned
Predicted
Difference

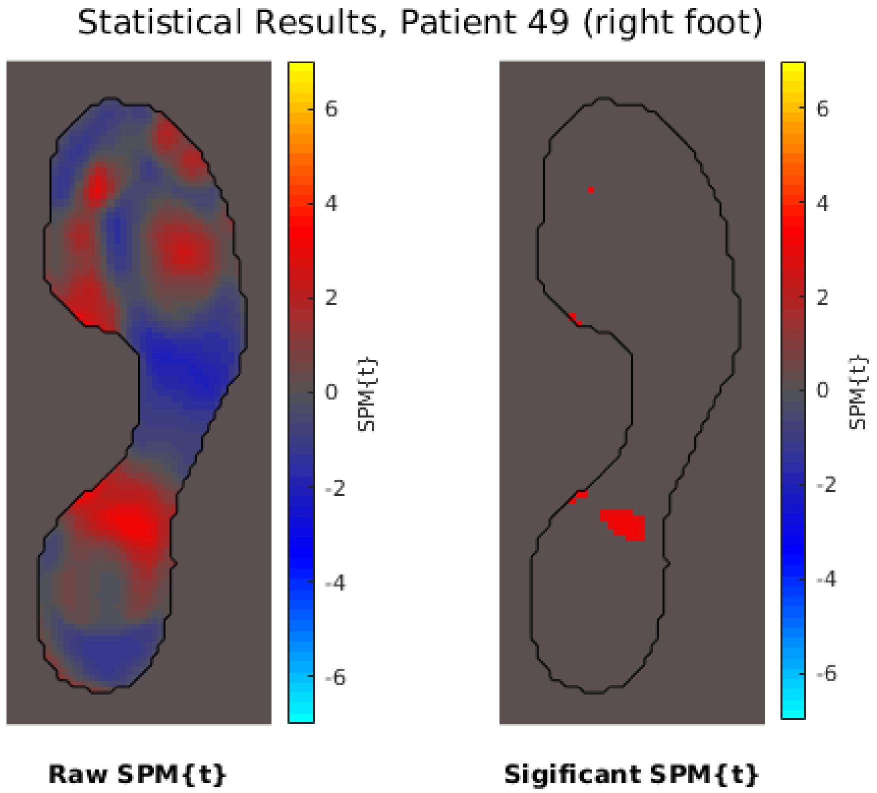
Statistical Results, Patient 49 (right foot)
6
4
2
0
-2
-4
-6
SPM{t}
Raw SPM{t}
Sigificant SPM{t}

Registration Results, Patient 50 (left foot)

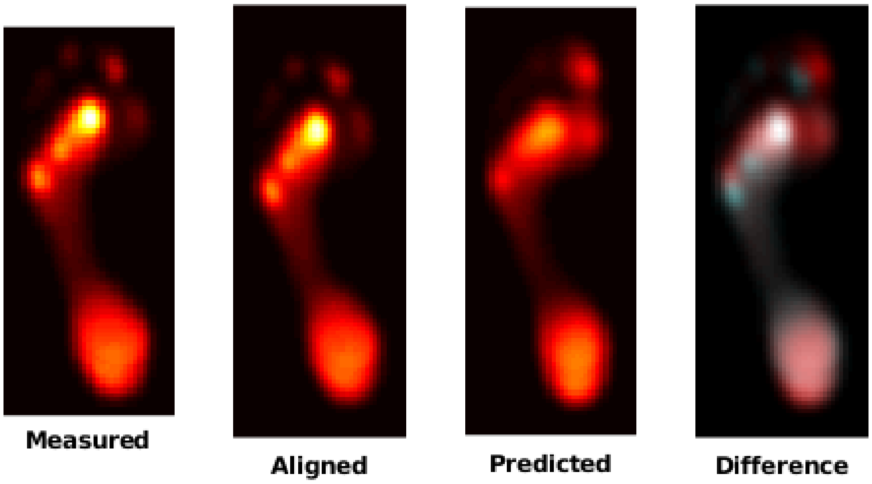

Statistical Results, Patient 50 (left foot)

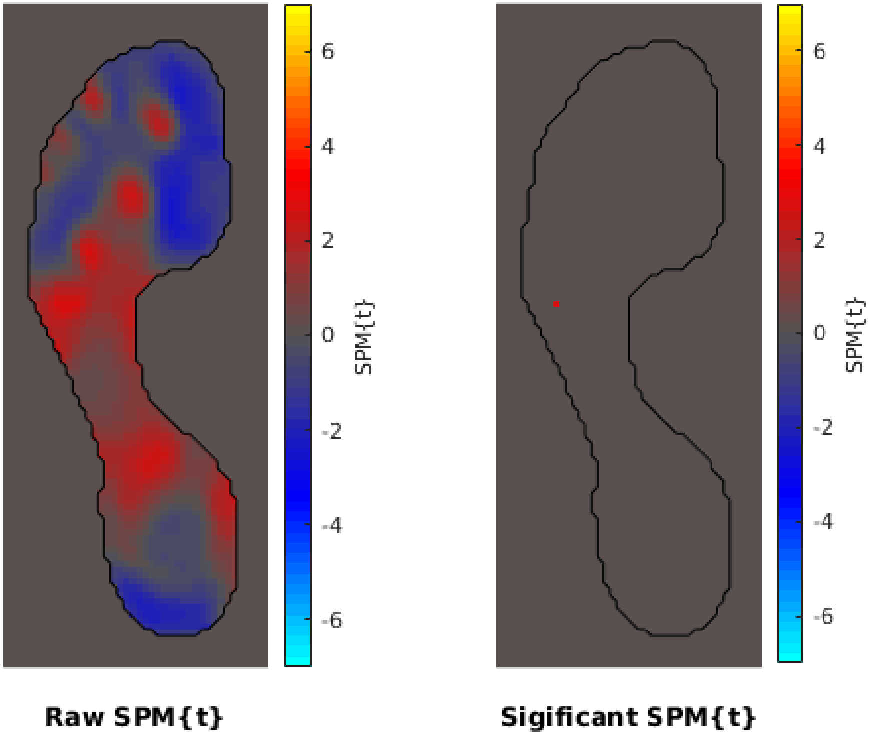

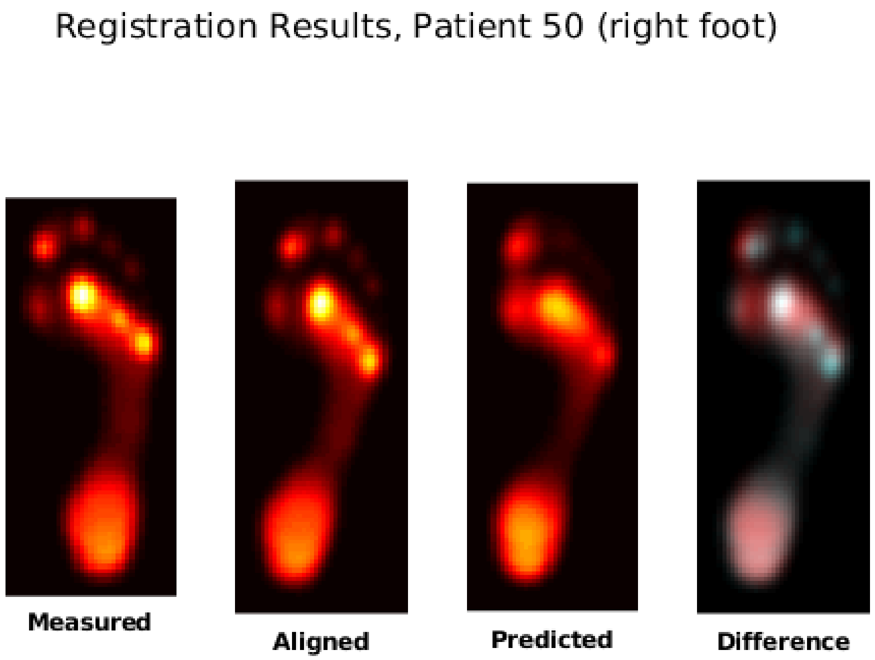
Registration Results, Patient 50 (right foot)
Measured
Aligned
Predicted
Difference

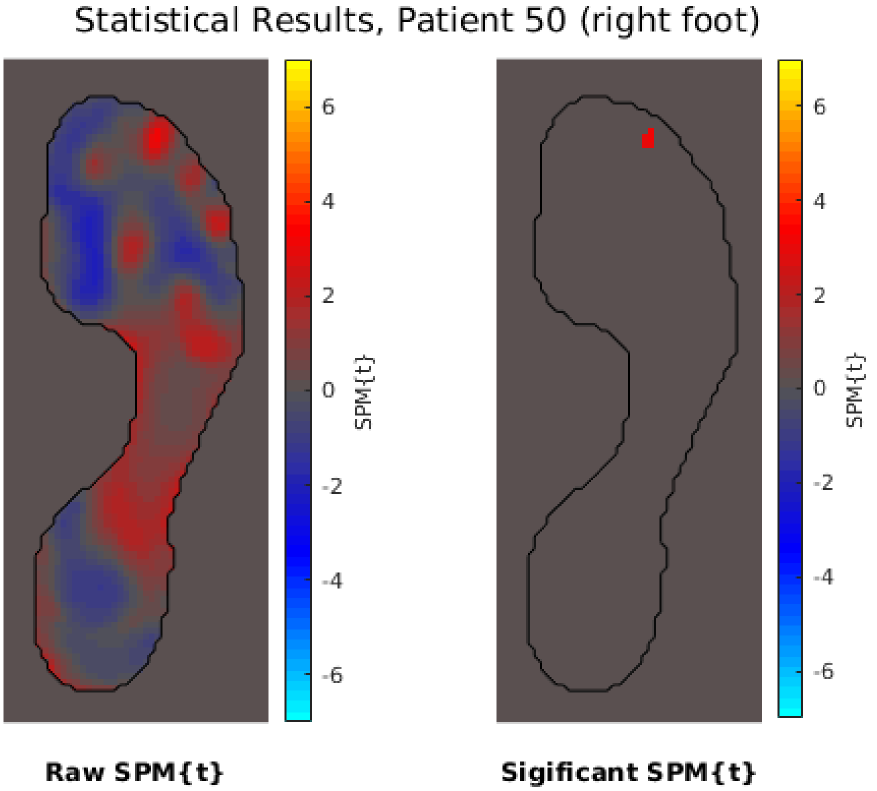
Statistical Results, Patient 50 (right foot)
6
4
2
0
-2
-4
-6
SPM{t}
Raw SPM{t}
Sigificant SPM{t}

Published with MATLAB® R2018b
