# Supplementary material for: PAPPI: Personalized analysis of plantar pressure images using statistical modelling and parametric mapping
Source: PLoS One. 2020 Feb 27;15(2):e0229685. doi: 10.1371/journal.pone.0229685 (PMC7046232; doi:10.1371/journal.pone.0229685)
Supplement: S2 File — The source code for PAPPI is provided in MATLAB format along with a readme file describing how it can be used. (ZIP) [file pone.0229685.s002.zip › PAPPI/README.pdf]

PAPPI: Personalized Analysis of Plantar Pressure Images
MATLAB Code, Version 1.0

1. Introduction

PAPPI is a MATLAB tool for the statistical analysis of plantar pressure images. It works by first building statistical models of the plantar pressures, pixel-by-pixel, from the measurements of healthy individuals. These models provide a healthy baseline to which a patient can be compared. PAPPI then compares a single person's plantar pressures to this baseline pixel-by-pixel. In both the modelling and the comparison, the demographic factors of age, sex, weight, height and shoe size, are taken into consideration in order to personalize the analysis to a particular individual. PAPPI is unique in its ability to provide this personalization while also reporting results pixel-by-pixel.

PAPPI is further defined in the following publication:

> * Booth, B.G., Hoefnagels, E., Huysmans, T., Sijbers, J., Keijsers, N.L.W. "PAPPI: Personalized analysis of plantar pressure images using statistical modelling and parametric mapping", PloS One (under review).

If you use this software in a publication, please cite this article.

2. Copyright and Licenses

All code for this project is subject to the following copyright and license:

Copyright (C) 2020 Brian G. Booth <brian.booth@uantwerpen.be>
imec-Vision Lab
Department of Physics
University of Antwerp
2610, Antwerp, Belgium

This code is published under the creative commons license Attribution-Noncommercial 3.0.
More information on this license is available at:

http://creativecommons.org/licenses/by-nc/3.0/deed.en_US

3. Installation and Setup

Within this folder, you will find six (6) MATLAB functions, one for each of the main tasks of PAPPI:

- *make_template.m:* This function builds the anatomically-unbiased peak pressure template from a set of peak pressure images.
- *align_images.m:* This function is used to bring one peak pressure image into alignment with another. It is used in two places in PAPPI: (a) to bring all healthy peak pressure images into alignment with the template, and (b) to bring a person's peak pressure image into alignment with the "baseline" peak pressure image estimated using the statistical models.
- *build_models.m:* This function builds the pixel-by-pixel statistical from a set of aligned peak pressure images and the corresponding demographics.
- *predict_peak_pressures.m:* This function estimates the baseline peak pressure image for a person with the given demographic characteristics.

- *compare_and_test.m:* This function compares a person's peak pressure image to their estimated baseline measurement using single-sample t-tests. This function assumes that the measured peak pressures are already aligned to the baseline image using *align_images.m.*
- *load_cad_walk_data.m:* This function provides the ability to read in all the plantar pressure measurements, and demographic information, from the CAD WALK plantar pressure database used in the PAPPI manuscript. If you are using different plantar pressure data, you will have to make your own function to perform this task for your dataset.

You should be able to use these functions directly without having to set anything up. For more information on each of these functions, type "help <function_name>" in MATLAB
(e.g. "help make_template").

4. Testing PAPPI

In the *examples* folder, you will find a MATLAB script (*test_PAPPI.m*) that will perform the experiment performed in the PAPPI article. To run this script, you will first have to download and unzip the plantar pressure measurements from the CAD WALK plantar pressure database. These plantar pressure measurements can be found at:

- Healthy Controls: https://doi.org/10.5281/zenodo.1265419
- Hallux Valgus: https://doi.org/10.5281/zenodo.1441308

In order to run the example script, please download and unzip these datasets into the *data* folder. Once those steps are complete, open MATLAB and run the *test_PAPPI* script found in the *examples* folder.

5. Contact Information

If you have any questions, concerns, or feedback with respect to this software, please send me an email at brian.booth@uantwerpen.be.
