# Supplementary material for: PAPPI: Personalized analysis of plantar pressure images using statistical modelling and parametric mapping
Source: PLoS One. 2020 Feb 27;15(2):e0229685. doi: 10.1371/journal.pone.0229685 (PMC7046232; doi:10.1371/journal.pone.0229685)
Supplement: Attachment — Submitted filename: Response_to_Reviewers.pdf [file pone.0229685.s003.pdf]

Response to Reviews

Paper Title: PAPPI: Personalized analysis of plantar pressure images using statistical modelling and parametric mapping

Paper ID: PONE-D-19-27376

We thank the reviewers and the senior editor for communicating their feedback on our manuscript. We have revised the manuscript by addressing the concerns raised as best as we could. In situations where we could not fully address a reviewer's concern with additions to the manuscript, we provide a detailed explanation as to why we are limited in our ability to do so.

In this response letter, we explain in detail how we addressed each comment. The reviewers' and editor's comments are copied and enumerated (e.g. R2.3 refers to the 3rd comment of reviewer 2) with our responses (in blue) interleaved. Changes to the manuscript are highlighted herein in purple. Unless stated otherwise, references in this response letter (to sections, figures, or bibliography entries) adopt the numbers as they appear in the revised manuscript. We have also highlighted the changes in our revised manuscript in purple.

The revisions suggested by the reviewers have further strengthened our manuscript and we look forward to your favorable consideration.

Sincerely,

Brian G. Booth, Eva Hoefnagels, Toon Huysmans, Jan Sijbers, and Noël L.W. Keijsers (the authors)

Comments from the Editor:

E1: Also, the zenodo data link does not specifically to a data file, but to a search page of different files. Please link directly to the complete dataset, ideally using a DOI.

This comment comes as a surprise to us. The provided Zenodo DOIs should link directly to the download pages for the datasets we used, and it is our understanding that this is in line with what you are requesting. For example, the DOI for the healthy controls dataset (http://doi.org/10.5281/zenodo.1265420) should link to the following page:

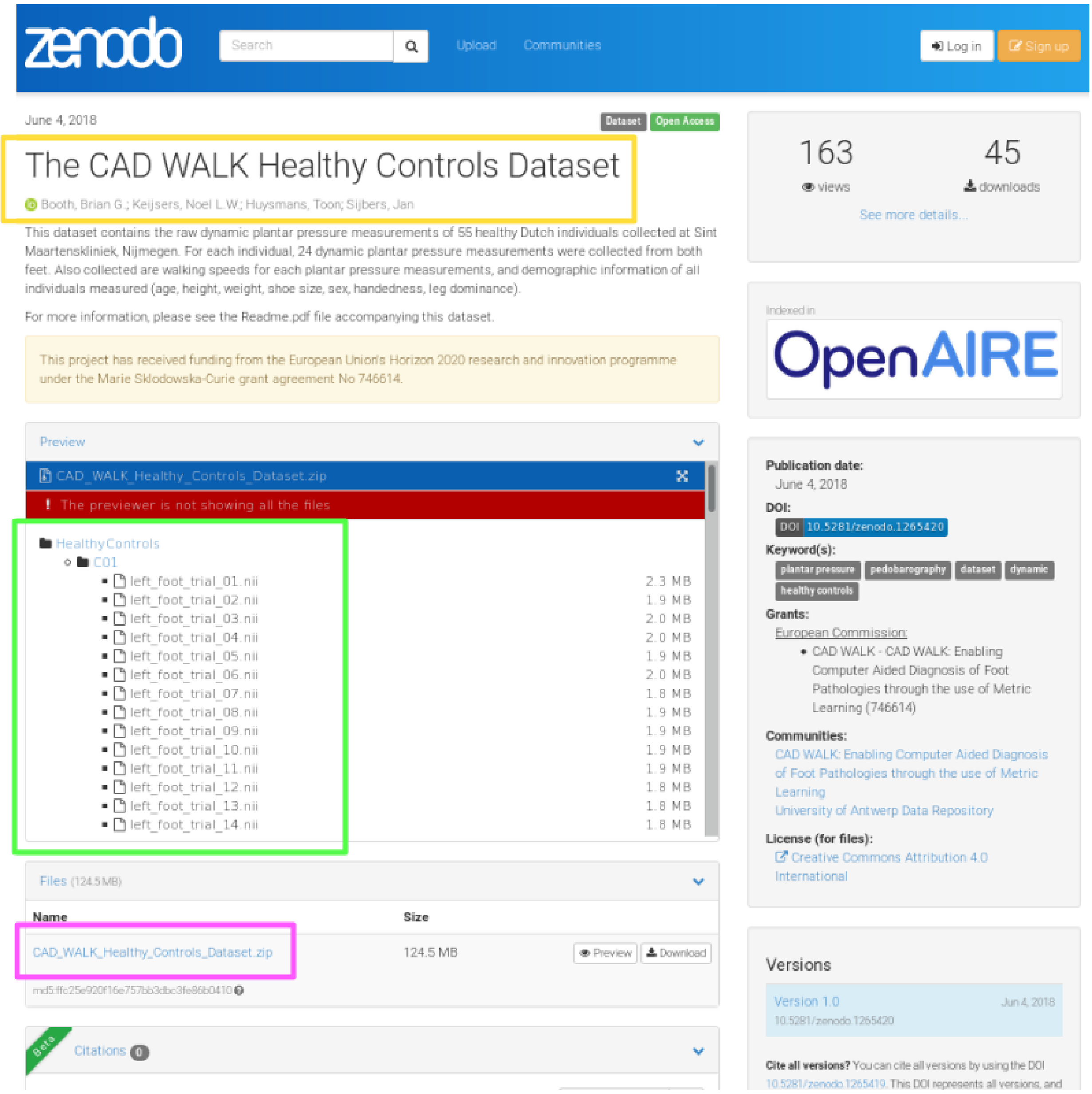

As you can see, this download page has the name of the dataset highlighted in yellow, the files of the dataset highlighted in green, and the download link highlighted in pink. If this is not what you are seeing, please do let us know what you see instead so that we can track down the problem.

E2: Please also be sure to correct for any other statistical analyses performed on the open dataset in other publications, or talk about such multiple comparisons issues briefly ...

The editor raises an important point. To the best of our knowledge, no other study has used the open-access datasets used in this study. We now make a note of this in the Data Collection section of the paper:

To our knowledge, no previous results have been reported on these datasets, so no multiple comparison issues exist between our study and previous works.

E3: Please ensure that your manuscript meets PLOS ONE's style requirements, including those for file naming.

We have double-checked our use of the PLOS ONE LaTeX template for the manuscript formatting as well as the file naming for our submitted documents. We believe that we do now meet the PLOS ONE style requirements. If we did miss something, please do let us know.

Comments from Reviewer #1:

R1.1: The paper presents a very innovative approach to assessing plantar pressure patterns on an individual level in order to better assess pathologies such as hallux valgus.

The paper is generally well written and represents an excellent contribution to the field. A few sections and the flowchart figures could be clearer. In the current form, it would be difficult for another researcher to reproduce this mythology in their lab, or for someone who is not very well versed in plantar pressure analyses to clearly understand the paper. Specific sections needing more clarity are outlined below in the specific comments.

We thank the reviewer for their positive opinion of our work. We agree with the reviewer that some parts of the paper could be made clearer. We indeed originally wrote the paper with a well-versed technical audience in mind, which may have been a mistake. To address this concern, apart from the requested changes to improve the paper’s clarity, we also made various minor changes to make the paper more accessible to a non-technical audience. These numerous changes are marked in purple in the revised manuscript.

Additionally, we have added the MATLAB code for PAPPI as a supplementary material file. In this way, other researchers should be able to reproduce PAPPI, even if some of the technical details are not clear. We have updated the Discussion section to note that this MATLAB code is provided:

We have also shared the MATLAB code for PAPPI as supplementary material (S14 File) in order to allow other groups to use and extend PAPPI as they see fit.

S14 File. MATLAB Code for PAPPI. The source code for PAPPI is provided in MATLAB format along with a readme file describing how it can be used.

R1.2: One other general comment relates to describing the accuracy of the statistical model in more detail. You describe PAPPI as, “The idea behind PAPPI is to statistically model, in a layered way, the healthy population plantar pressures and the demographic factors that can influence them.” PAPPI is explained as being built off of 55 healthy controls. The model seems to then be directly used to test hallux valgus patients as a proof of concept of how the model can be used to identify/assess pathology. There is potentially a step missing that would involve showing that the model accurately predicts a healthy control. Was the model performance validated in any way? Since the model uses a 95% confidence interval, we would expect 95% of healthy subjects tested to show no statistical differences from their estimated pressure to their actual pressure. The paper references 23% of hallux valgus patients showing no difference from their estimated “healthy” plantar pressure. “Note that this number is much lower than the 95% of healthy cases we would expect to be free of abnormalities based on the definition of our statistical model.” Was this statement - the accuracy of the model - validated? Without information on the accuracy of the estimated plantar pressure there’s the potential to draw some incorrect conclusions based on it. Maybe this is another paper, and not necessarily needed for the current paper, but I would guide caution in interpreting results too much until the accuracy is verified.

The reviewer raises an excellent point. Like he or she says, we used a 95% significance threshold in the model, so we would hypothesize that 95% of healthy individuals would show no plantar pressure abnormalities using PAPPI. However, we did not mention whether this hypothesis is validated on a separate dataset of healthy controls. In preparing this revision, we attempted to perform a validation test, but we ran into a couple of difficulties:

1. We could not find a second publicly-available dataset of plantar pressures from healthy controls. Given PLoS One’s strict data sharing rules, the use of another, private, dataset would make our manuscript ineligible for publication.
2. We looked at using cross validation in order to validate PAPPI (i.e. use 55-X individuals for the modelling, then use the X individuals left out the modelling to test the validity of the model. Repeat until all individuals are included in the testing), but we noticed that the cross validation results were both lower than the expected 95% and sensitive to the number of individuals included in the model. This makes it difficult to interpret whether (a) our modelling technique is valid, but we need more data, or (b) our modelling technique does not satisfy the above hypothesis. We suspect that it is the former for two reasons. First, the accuracy of the model improved as more individuals were included in it. Second, in statistical modelling, we have the one-in-ten rule: one predictive variable (e.g. parameter in the statistical model) can be studied for every ten events (e.g. individuals in the model) [C1]. In our case, we have 7 parameters in our model (the 5 coefficients of the linear regression model, the bias parameter in the linear regression model, and the standard deviation of the residuals), which means that we would require 70 individuals to be included in the model to satisfy this rule and, in turn, perform a proper validation study. We are below this threshold.

Because of these difficulties, we decided that a proper validation study was a large enough topic to warrant a follow-up paper. Nevertheless, we have made two changes in our manuscript to address this concern. First, we removed the offending statement highlighted by the reviewer. Second, we have included the following paragraph in the Discussion section noting the lack of a validation study and emphasizing that these results should only serve as a proof-of-concept:

Despite the strengths of PAPPI, we would caution against over-interpretation of the results presented here. One key reason for exercising caution is the limited number of individuals (55) included in the statistical model. When building statistical models, it is typically advised that the model satisfy the one-in-ten rule: that one predictive variable is studied using a minimum of ten events [52]. In this work, our pixel-by-pixel statistical models contain 7 predictive variables: the five linear regression coefficients for the demographics factors, the bias variable in the linear regression, and the standard deviation of the residuals. Using the one-in-ten rule, at least 70 individuals would be desirable for building the statistical model and ensuring reasonable validity. We are currently unable to hit this threshold due to a lack of available data. As a result, it may be the case that some of the abnormalities observed in the hallux valgus patients may be due to the fact that the 55 individuals in our statistical model provide an incomplete view of a true healthy population. There is some evidence that this might be the case since the abnormalities seen in some foot regions did not correlate with either the foot function scores or the radiographic measurements. Nevertheless, what we have shown is a proof-of-concept for the methodology behind PAPPI. A full validation of PAPPI, including an evaluation of the one-in-ten rule for this framework, is planned as future work.

[52] Harrell FE, Lee KL, Mark DB, Multivariable Prognostic Models: Issues in Developing Models, Evaluating Assumptions and Adequacy, and Measuring and Reducing Errors. Statistics in Medicine, 1996;15:361-387.

R1.3: P4, L100. Any concern with the healthy controls and hallux valgus patients being collected at different sampling frequencies? (500 Hz vs. 200 Hz)

To our knowledge, this should not be a concern. The pressure plate agrees with international standards, so it should measure plantar pressures accurately at both of these sampling frequencies. Also, we see no reason why the sampling frequency would affect an individual's gait. We now note this in the Data Collection section of our manuscript:

Given the international standards for pressure-sensing plates [2], the difference in sampling frequency is not expected to influence the accuracy of the measurements.

[2] Giacomozzi C, Keijsers N, Pataky T, Rosenbaum D. International scientific consensus on medical plantar pressure measurements devices: technical requirements and performance. Annali dell'Istituto Superiore di Santa. 2012;48(3):259–271.

R1.4: P5. Figure 1. The flow chart is not clear to me. What is meant by "a linear model of the peak pressures at each pixel", and "Normal distributions of the model residuals"? I have a similar issue for Figure 2 – would be great to be able to understand a big picture of what you did from looking at the diagram.

We agree with the reviewer that the flow charts could be clearer. The previous manuscript used flow charts to show the order in which algorithms were applied and not to show the big picture. To address this concern we have redrawn Figures 1 and 2 (now Figures 2 and 3, respectively) to focus more on why each step is being done instead of what steps are being

done. We have also updated the text in the introduction and methods sections to present the high level description of PAPPI in a similar manner.

In the revised manuscript, Figure 2 now looks as follows:

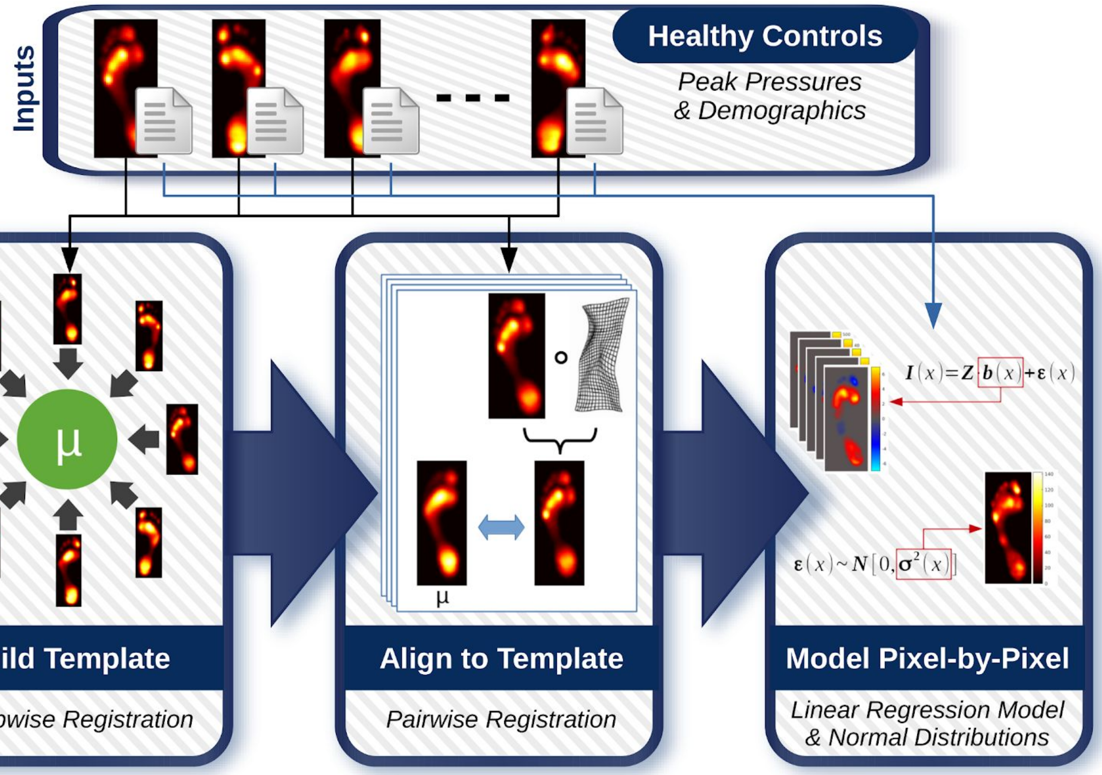

Fig 2. Flow chart of Statistical Modelling Workflow. The proposed PAPPI technique begins by creating an anatomically-unbiased template to which all healthy peak pressure images are aligned. Once aligned, statistical models are built pixel-by-pixel in order to provide localized statistical analysis. Specifically, the images and their corresponding demographic factors are used to build (a) linear regression models of the peak pressures and (b) Normal distributions of the model residuals.

As before, the flow chart shows three tasks, but now each task is identified by what it is meant to accomplish instead of the name of the algorithm used in the task. The algorithm names have been relegated to below the task names. Additionally, a more detailed visual description has been added to each task. With respect to the comments about the meaning of linear models and residuals, we were referring to (a) the use of multiple linear regression to model the relationship between peak pressures and demographic factors, (b) the use of normal distributions to model the portion of the peak pressures that is not explained by the multiple linear regression model, and (c) that we compute these models pixel-by-pixel. We hope that this new figure makes these points clearer.

We took a similar approach when revising Figure 3 which now looks like this:

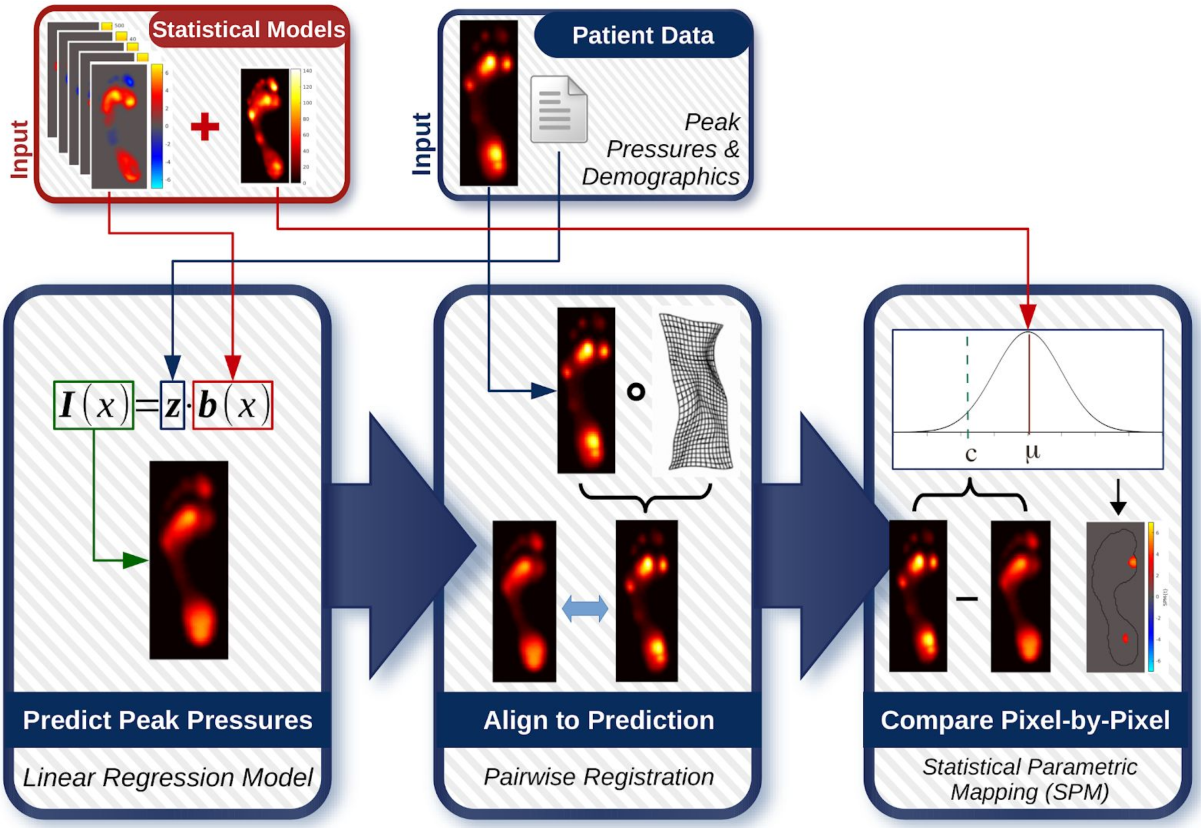

Fig 3. Flow chart of Statistical Testing Workflow. Given a peak pressure image and demographic characteristics from a new patient, a healthy peak pressure image for the patient is estimated using the statistical model. The patient's measured pressures are then aligned to the estimated image, and a residual image is created by subtracting the estimated pressures from the measured ones. Finally, statistical parametric mapping is used with single-sample t-tests in order to identify patient residuals that are outliers from the statistical model's Normal distributions over the residuals.

In addition to these new figures, we have updated the text to align with the presentation format used in the figures. In the introduction, we first update our description of Statistical Parametric Mapping (SPM) to provide its "big picture" as this will serve as the starting point for the big picture of our algorithm:

In particular, statistical parametric mapping (SPM) techniques have recently gained popularity in the performance of these group studies, whether they be for region-of-interest studies [8], centre of pressure trajectories [9], pressure pattern images [10], or plantar pressure videos [11]. SPM works by bringing all plantar pressure measurements into anatomical (and possibly also temporal) alignment, then performing statistical tests at each sampled point (e.g. each pixel in an image or each time point in a sequence). By performing statistics in this manner, SPM localizes and highlights regions of plantar pressures that show statistically significant group differences, thereby simplifying the interpretation of a study's results.

[8] Pataky TC, Vanrenterghem J, Robinson MA. Zero- vs. one-dimensional, parametric vs. non-parametric, and confidence interval vs. hypothesis testing procedures in one-dimensional biomechanical trajectory analysis. Journal of Biomechanics. 2015;48:1277–1285.

[9] Pataky TC, Robinson MA, Vanrenterghem J, Savage R, Bates KT, Crompton RH. Vector field statistics for objective center-of-pressure trajectory analysis during gait, with evidence of scalar sensitivity to small coordinate system rotations. Gait & Posture. 2014;40(1):255–258.

[10] Pataky TC, Goulermas JY. Pedobarographic statistical parametric mapping (pSPM): A pixel-level approach to foot pressure image analysis. Journal of Biomechanics. 2008;41:2136–2143.

[11] Booth BG, Keijsers NLW, Sijbers J, Huysmans T. STAPP: spatiotemporal analysis of plantar pressure measurements using statistical parametric mapping. Gait & Posture. 2018;63:268–275.

Next, we update our description of PAPPI in the introduction to match both the Template-Align-Model narrative in Figure 2 as well as the Predict-Align-Compare narrative in Figure 3:

Despite all the advances in the analysis of plantar pressure measurements, there remains a need for an analysis technique that provides both a personalized analysis of a person’s plantar pressures while also localizing abnormal pressure measurements to precise locations on the foot. The objective of this paper is to fill that gap with PAPPI: the Personalized Analysis of Plantar Pressure Images. Fig 1 shows how PAPPI relates to other plantar pressure analysis techniques. At a high level, PAPPI combines the localization benefits of SPM with the personalization benefits of classification algorithms. Like SPM, PAPPI is based on the idea of bringing plantar pressure measurements into anatomical alignment, then performing statistics at each pixel. Unlike SPM, PAPPI employs a statistical outlier detection algorithm to classify plantar pressure abnormalities pixel-by-pixel [31, 32]. This outlier detection involves the pixel-by-pixel modelling of plantar pressures from a healthy population as well as the relationship between those pressures and demographic factors such as age, weight, and gender. This model then serves as a healthy baseline to which an individual’s plantar pressures are compared, pixel-by-pixel, using single-sample t-tests. Plantar pressures that do not agree with the model are then classified as abnormalities and are highlighted for display.

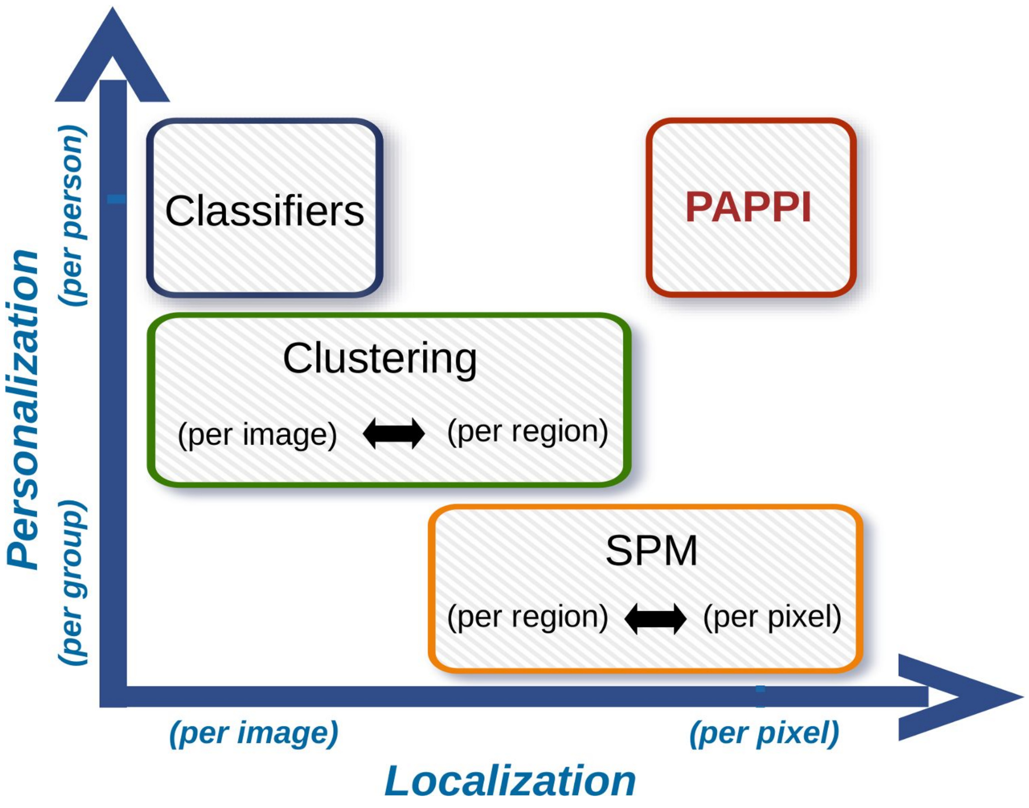

Fig 1. The positioning of PAPPI with respect to competing plantar pressure analysis techniques. The proposed PAPPI technique is unique in providing a highly personalized result (i.e. one result per foot) while also localizing plantar pressure abnormalities to a high degree (i.e. pixel-by-pixel abnormality identification). See text for further details.

With PAPPI, we introduce two methodological contributions. First, we introduce pixel-by-pixel outlier detection to the analysis of plantar pressure measurements. Second, we incorporate the impact of multiple demographic factors into the statistical outlier detection, thereby allowing PAPPI to fine tune its outlier detection to specific individuals. With these contributions, we aim to provide the intuitive summaries of abnormal plantar pressures that SPM is known for while also personalizing the SPM procedure in a way that accounts for the natural variability in plantar pressure measurements.

[31] Booth BG, Miller SP, Brown CJ, Poskitt KJ, Chau V, Grunau RE, et al. STEAM - Statistical Template Estimation for Abnormality Mapping: A personalized DTI analysis technique with applications to the screening of preterm infants. NeuroImage. 2016;125:705–723.

[32] Stankovic K, Danckaers F, Booth BG, Burg F, Duerinck S, Sijbers J, et al. Foot Abnormality Mapping using Statistical Shape Modelling. In: Proceedings of International Conference and Exhibition on 3D Body Scanning Technologies (3DBST); 2016. p. 70–79.

Additionally, we have updated the text at the beginning of the Statistical Modelling section to provide more of a big picture description of what is being done and why:

Fig 2 shows the workflow of the proposed statistical modelling task in PAPPI. The workflow contains three main components. First, an anatomically-unbiased peak pressure template is created to which all measurements will be aligned for the subsequent analysis. Second, all peak pressure measurements are aligned to said template so that we have a pixel-by-pixel

anatomical correspondence between all individuals' peak pressures. Finally, statistical models are built at each pixel to model both the relationship between the peak pressures and demographic factors as well as the fraction of the peak pressures that are not explained by the demographics. We introduce each of these steps below.

Finally, we also updated the big picture description at the beginning of the Statistical Testing section:

The personalized evaluation of a patient's peak pressure image follows the workflow shown in Fig 3 and is similar to outlier detection techniques seen elsewhere [31, 32]. The patient's demographic factors (age, sex, weight, height, shoe size) are inputted into our linear regression model to predict their peak pressure image. The patient's real peak pressures, acquired by measurement, are then aligned to the predicted image in order to obtain a pixel-by-pixel anatomical correspondence between the two peak pressure images. Once aligned, the residuals between the measured and predicted peak pressures are computed and compared to the Normal distributions in our statistical model. Any outlier pressures are then highlighted using SPM.

[31] Booth BG, Miller SP, Brown CJ, Poskitt KJ, Chau V, Grunau RE, et al. STEAM - Statistical Template Estimation for Abnormality Mapping: A personalized DTI analysis technique with applications to the screening of preterm infants. NeuroImage. 2016;125:705–723.

[32] Stankovic K, Danckaers F, Booth BG, Burg F, Duerinck S, Sijbers J, et al. Foot Abnormality Mapping using Statistical Shape Modelling. In: Proceedings of International Conference and Exhibition on 3D Body Scanning Technologies (3DBST); 2016. p. 70–79.

We hope that these changes provide the reviewer with the context required to understand the proposed workflow better. If the paper could benefit from further clarifications, please do let us know.

R1.5: P5. L146. This paragraph is not clear. You mention that you consider the left and right foot as being independent, but then in the last sentence of the paragraph (line 150) you mention that you flip the right foot to treat each image as containing peak pressures from the left? Please re-write in a clearer way.

We thank the reviewer for pointing out the confusion here. We believe the source of this confusion is that a bit of context is missing between the two statements the reviewer pointed out. We have updated this paragraph to include this context as follows:

Finally, we follow the convention of Pataky et al. by assuming that there is no common asymmetry pattern between a person's left and right plantar pressures, thereby allowing us to regard the plantar pressures from an individual's left and right feet as being essentially independent samples [42]. Using this independence assumption, we can group the left and right feet into a single statistical model. In this work, we chose to build a single "left" foot model. To do so, peak pressure images from right feet are flipped along the medial-lateral axis in order to simplify the subsequent model building and analysis steps.

[42] Pataky TC, Bosch K, Mu T, Keijsers NLW, Segers V, Rosenbaum D, et al. An anatomically unbiased foot template for inter-subject plantar pressure evaluation. Gait & Posture. 2011;33:418–422.

R1.6: P9. L276. Can you describe how each region of the foot was defined?

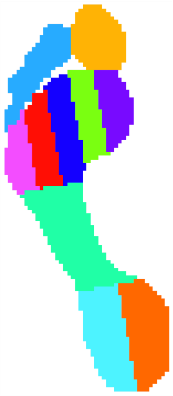

Certainly. We defined regions in two steps. First we began with the Novel 10 region foot map shown on the left [7]. However, we also wanted to have enough results in each region in order to perform a statistical analysis. In order to accomplish this, we merged some regions: the medial and lateral heel, metatarsals 2-5, and the hallux and small toes. This left us with the five regions mentioned in the paper: toes, metatarsal 1, metatarsals 2-5, midfoot, and heel. These mergings provided us with at least 12 feet showing peak pressure abnormalities in each region. This number of feet gave us a statistical power of 0.8 for detecting group differences of one standard deviation or more. We felt that this was as good a trade-off between region specificity and statistical power as we could get with these publicly-available datasets.

To clarify this in the paper, we have added the following text:

To summarize all of our PAPPI results, we present them according to which foot regions contained abnormal peak pressures: the heel, midfoot, metatarsal 1, metatarsals 2-5, and toes. We defined these regions using the Novel 10 region mask [7], with some regions merged (e.g. medial and lateral heel, metatarsals 2-5, hallux and other toes) in order to increase the statistical power in subsequent following experiments. Note that, in contrast to Table 1, we combined the hallux and lesser toes into a single group, as well as isolating metatarsal 1 from the rest of the forefoot, as these groupings aligned more closely with the results we observed.

[7] Keijsers NLW. Plantar Pressure Analysis. In: The Science of Footwear. CRC Press; 2012. p. 377–409.

R1.7: P11. L337. One aspect of the discussion that is missing is how the surface area that makes contact with the ground of each individual is considered. In the figures, it appears as though all plantar pressure patterns have a similar surface area (seems like a high-arch individual). Can the surface areas that make contact with the pressure pad be used in the assessment? For example, flat-footed individuals would have more surface area at the medial midfoot.

This is a very good point. In this work, we have used the surface area of the template for the statistical modelling and analysis. This template is the average of our healthy controls, so it will have the average surface area seen in the measurements from our healthy controls. We also note that these peak pressure images were computed from dynamic plantar pressure measurements collected from walking subjects. It has been reported that dynamic movement leads to less midfoot pressure [11] and a smaller midfoot surface area than static peak pressure measurements [C1]. Based on the way the template was made and this previous literature, we believe that our analysis is using the surface area of a normal-arched individual.

That being said, it would indeed be valuable for PAPPI to take arch height into account. In fact, we had multiple internal discussions about whether arch height should be included with

the demographic information as a covariate in our statistical model, perhaps through the use of the arch index [51]. This would have allowed the statistical model to adapt the midfoot surface area based on the individual. We eventually decided against doing this however as it would mean that the prediction of the patient's healthy baseline would depend on a measurement (i.e. arch index) that was computed from his or her unhealthy foot. We were not sure that such a dependence was appropriate as a person's arch height might be affected by their foot injury or pathology. As a result, we omitted arch height from the statistical modelling in this work. This may be something we look at again in our future work.

The reviewer's point is well taken and we have summarized the above response in the Discussion section of our revised manuscript:

It should also be noted that PAPPI makes two simplifying assumptions in the statistical modelling... Second, we assume that the residuals in our linear regression model follow a normal distribution... This normal distribution assumption will also affect the amount of the midfoot that gets evaluated. PAPPI currently uses the plantar surface area of the template as a boundary in order to increase the chances that this normal distribution assumption holds for all statistical analyses. Unfortunately, this boundary can omit some of the plantar surface area measured from individuals with pes planus. Nevertheless, PAPPI was able to observe the effects of pes planus through increased midfoot pressures (see Fig 7). Removing this normal distribution assumption, thereby allowing for modelling of variable surface areas, is also a potential area of future work.

[11] Booth BG, Keijsers NLW, Sijbers J, Huysmans T. STAPP: spatiotemporal analysis of plantar pressure measurements using statistical parametric mapping. Gait & Posture. 2018;63:268–275.

[C1] Kim T, Park J-C. Short-term effects of sports taping on navicular height, navicular drop and peak plantar pressure in healthy elite athletes. Medicine. 2017;96(46):e8714.

[51] Cavanagh PR, Rodgers MM. The Arch Index: A useful measure from footprints. Journal of Biomechanics. 1987;20(5):547–551.

R1.8: P11. L351. Typo: remove word "in".

We thank the reviewer for pointing out this typo. We have corrected the sentence and for clarity. It now reads as follows:

Ultimately, the variability in the results produced by PAPPI may disambiguate the results seen at the group level in previous studies, thereby highlighting the need and the value of this personalized analysis technique in evaluating plantar pressures.

Once again, we thank the reviewer for their thorough review and we hope that the changes we have made to the manuscript meet with his or her satisfaction.

Comments from Reviewer #2:

R2.1: In your manuscript, you introduce a general statistical method for quantitative analyze of the image. GLMs are robust tools that can handle high dimension linear relation and should be of interest for the bio imaging field.

We thank the reviewer for their positive response to our work.

R2.2: In this manuscript, authors propose a multi-step strategy where they consider some transformation on the data without explaining the main structure,

It is unclear to us how we should address this comment because we are unsure what the reviewer means by “main structure”. Is the reviewer referring to the structure of the transformations applied to the images, the structure of the images themselves, the anatomical structure of the foot that the peak pressures were taken from, or something else? Perhaps this comment comes from the same lack of manuscript clarity that was pointed out in comment R1.4. In that case, we refer the reviewer to our response to that comment to see if that is indeed the case. If not, then we would welcome more specific feedback on how we can address this point.

Additionally, we would also like to note that the algorithms we use to transform the peak pressure images are the same algorithms that have been used for over a decade in plantar pressure statistical parametric mapping studies [10,11,40]. We also qualitatively evaluate the accuracy of these transformations in Figure 4 and supplementary file S13. As a result, we believe that the transformations we are doing are appropriate for our statistical analysis framework.

[10] Pataky TC, Goulermas JY. Pedobarographic statistical parametric mapping (pSPM): A pixel-level approach to foot pressure image analysis. Journal of Biomechanics. 2008;41:2136–2143.

[11] Booth BG, Keijsers NLW, Sijbers J, Huysmans T. STAPP: spatiotemporal analysis of plantar pressure measurements using statistical parametric mapping. Gait & Posture. 2018;63:268–275.

[40] Pataky TC, Bosch K, Mu T, Keijsers NLW, Segers V, Rosenbaum D, et al. An anatomically unbiased foot template for inter-subject plantar pressure evaluation. Gait & Posture. 2011;33:418–422.

R2.3: ...in addition a Normal distribution as a basic assumption

The reviewer is correct that PAPPI relies on the assumption that the fraction of the peak pressures unexplained by demographics follow a Normal distribution. We understand that this may be a concern. For that reason, we confirmed the suitability of this assumption using Kolmogorov-Smirnov tests as shown in Figure 12. Nevertheless, it may be the case that this assumption fails for certain cohorts, and so we now make a note of that in the Discussion section:

It should also be noted that PAPPI makes two simplifying assumptions in the statistical modelling... Second, we assume that the residuals in our linear regression model follow a normal distribution. While this was generally the case in our study (see Fig 12), the validity of this assumption is not always guaranteed. We would recommend that this assumption will be checked whenever PAPPI is used.

R2.4: ...and estimate the coefficients b(x) of GLM without mention the estimation method.

The reviewer is correct that we forgot to mention how we computed the coefficients of the GLM. We did so using ordinary least squares. We now make a note of this in the Model Building section of the manuscript:

At each pixel x, we employ ordinary least squares to fit a linear model

$$y(x) = Zb(x) + \varepsilon(x), \qquad (7)$$

where $y(x) = [I'_1, \cdots, I'_N]^T$ are the peak pressures at pixel **x**, $Z = [z_1, \cdots, z_N]^T$ is a matrix containing the corresponding participant's demographic factors, and **b**(x) are the model parameters that indicate the influence of each factor on the peak pressures.

R2.5: While GLM and statistical modeling and parameter mapping have been, extensively used in various fields, it would be better- based on my knowledge- to consider the nonlinear view which the main part of the image and movement to make a better predict model.

We agree with the reviewer that a non-linear modelling approach may yield even better results. In fact, that is an idea we are currently exploring with the CAD WALK project (http://cadwalk.eu). As part of that project, we would like to evaluate the amount of improvement we will see when moving from a linear model to a non-linear one. This evaluation requires results using a linear model, results that do not currently appear in the literature. This submitted manuscript provides those results and we intend to use PAPPI as the baseline to which our CAD WALK contributions can be compared. We now make a note of this in the discussion section of our manuscript:

It should also be noted that PAPPI makes two simplifying assumptions in the statistical modelling. First, we assume that the relationship between peak pressures and demographic factors is a linear one. While there is some evidence to support this claim [18, 19], it does not rule out the possibility that a non-linear regression model could improve upon these results. Consequently, the use of non-linear models is something we are currently exploring in our CAD WALK project (http://cadwalk.eu).

[18] Keijsers NLW, Stolwijk NM, Louwerens JWK. The effect of various subject characteristics on plantar pressure pattern. Journal of Foot and Ankle Research. 2014;7(1):A40.

[19] Telfer S, Bigham JJ. The influence of population characteristics and measurement system on barefoot plantar pressures: A systematic review and meta-regression analysis. Gait & Posture. 2019;67:269–276.

R2.6: The manuscript is interesting however, it needs more clarity in mathematical derivations and notations,

As part of this revision, we performed a thorough review of the Methods section and did our best to clarify the content and the notations used in each equation. These changes are highlighted in purple in the revised manuscript. In general, we followed the notation style of

Vercauteren [43] for the image registration equations and the notation style of Pataky and Goulermas [10] for the statistics. If any mathematical issues persist, please do let us know specifically where they are. We will be happy to correct any remaining errors.

[10] Pataky TC, Goulermas JY. Pedobarographic statistical parametric mapping (pSPM): A pixel-level approach to foot pressure image analysis. Journal of Biomechanics. 2008;41:2136–2143.

[43] Vercauteren T, Pennec X, Perchant A, Ayache N. Diffeomorphic demons: Efficient non-parametric image registration. NeuroImage. 2009;45(1):S61–S72.

R2.7: and it should be, compared to quite robust statistical methods to make the novelty clearer.

We agree with the reviewer that the novelty of PAPPI should be clarified by comparing it to similar works. To address this point, we have updated the Introduction section to include a conceptual comparison of the related works presented there:

Despite all the advances in the analysis of plantar pressure measurements, there remains a need for an analysis technique that provides both a personalized analysis of a person's plantar pressures while also localizing abnormal pressure measurements to precise locations on the foot. The objective of this paper is to fill that gap with PAPPI: the Personalized Analysis of Plantar Pressure Images. Fig 1 shows how PAPPI relates to other plantar pressure analysis techniques. At a high level, PAPPI combines the localization benefits of SPM with the personalization benefits of classification algorithms. Like SPM, PAPPI is based on the idea of bringing plantar pressure measurements into anatomical alignment, then performing statistics at each pixel. Unlike SPM, PAPPI employs a statistical outlier detection algorithm to classify plantar pressure abnormalities pixel-by-pixel [31, 32]. This outlier detection involves the pixel-by-pixel modelling of plantar pressures from a healthy population as well as the relationship between those pressures and demographic factors such as age, weight, and gender. This model then serves as a healthy baseline to which an individual's plantar pressures are compared, pixel-by-pixel, using single-sample t-tests. Plantar pressures that do not agree with the model are then classified as abnormalities and are highlighted for display.

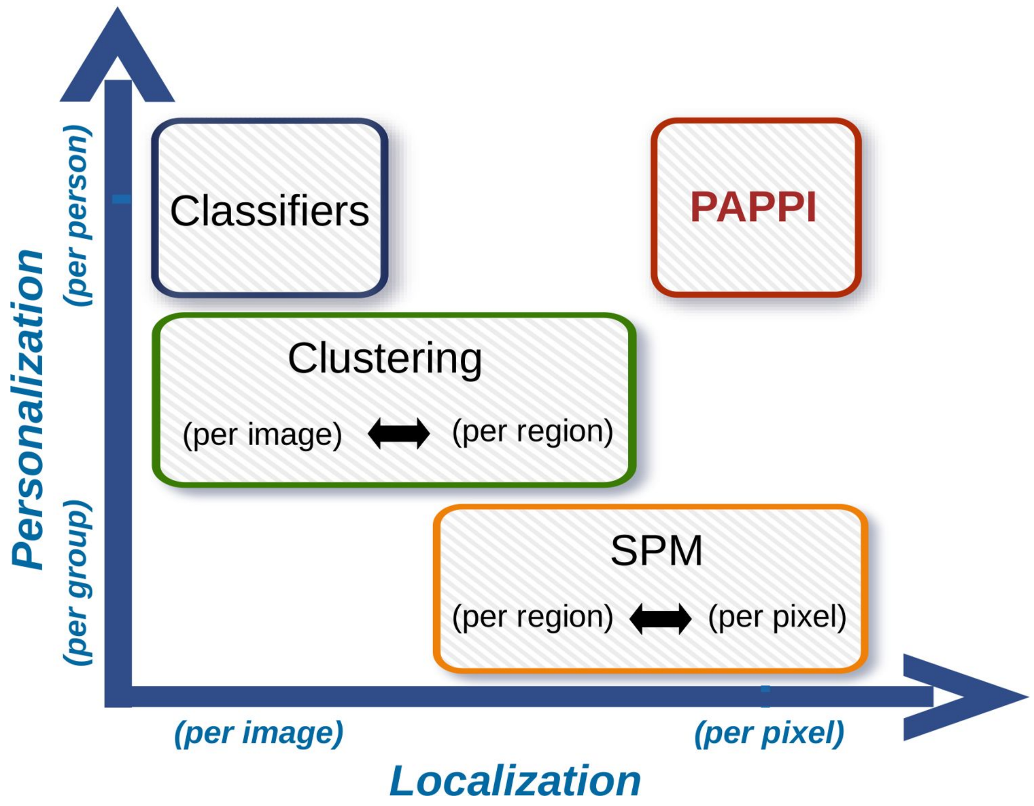

Fig 1. The positioning of PAPPI with respect to competing plantar pressure analysis techniques. The proposed PAPPI technique is unique in providing a highly personalized result (i.e. one result per foot) while also localizing plantar pressure abnormalities to a high degree (i.e. pixel-by-pixel abnormality identification). See text for further details.

With PAPPI, we introduce two methodological contributions. First, we introduce pixel-by-pixel outlier detection to the analysis of plantar pressure measurements. Second, we incorporate the impact of multiple demographic factors into the statistical outlier detection, thereby allowing PAPPI to fine tune its outlier detection to specific individuals. With these contributions, we aim to provide the intuitive summaries of abnormal plantar pressures that SPM is known for, while also personalizing the SPM procedure in a way that accounts for the natural variability in plantar pressure measurements.

[31] Booth BG, Miller SP, Brown CJ, Poskitt KJ, Chau V, Grunau RE, et al. STEAM - Statistical Template Estimation for Abnormality Mapping: A personalized DTI analysis technique with applications to the screening of preterm infants. NeuroImage. 2016;125:705–723.

[32] Stankovic K, Danckaers F, Booth BG, Burg F, Duerinck S, Sijbers J, et al. Foot Abnormality Mapping using Statistical Shape Modelling. In: Proceedings of International Conference and Exhibition on 3D Body Scanning Technologies (3DBST); 2016. p. 70–79.

R2.8: English language of manuscript also must be improved

The revised manuscript has been thoroughly proofread by a native English speaker and their corrections are highlighted in purple in the manuscript. Any issues related to the use of the English language should now be corrected. If that is not the case, we would be grateful if the remaining errors can be pointed out specifically as was done with comment R1.8, for example. We will be happy to correct any further errors in English usage that are made aware to us.

Finally, we thank the reviewer for their comments. We hope that the changes we have made to the manuscript meet with his or her satisfaction. If that is not the case, we would welcome further feedback on how the manuscript could be improved.
